# Beneficial effect of the short-chain fatty acid propionate on vascular calcification through intestinal microbiota remodelling

**DOI:** 10.1186/s40168-022-01390-0

**Published:** 2022-11-16

**Authors:** Jianlong Yan, Yanbin Pan, Wenming Shao, Caiping Wang, Rongning Wang, Yaqiong He, Min Zhang, Yongshun Wang, Tangzhiming Li, Zhefeng Wang, Wenxing Liu, Zhenmin Wang, Xin Sun, Shaohong Dong

**Affiliations:** 1 Department of Cardiology, Shenzhen Cardiovascular Minimally Invasive Medical Engineering Technology Research and Development Center, Shenzhen People’s Hospital (The Second Clinical Medical College, Jinan University; The First Affiliated Hospital, Southern University of Science and Technology), Shenzhen, 518020 Guangdong China; 2 Department of health management center, Shenzhen People’s Hospital (The Second Clinical Medical College, Jinan University; The First Affiliated Hospital, Southern University of Science and Technology), Shenzhen, 518020 Guangdong China; 3 Department of Emergency, The First Affiliated Hospital of Jinan University, Guangzhou, 510630 Guangdong China; 4 Department of Spine Surgery, the Second Clinical Medical College, Jinan University (Shenzhen People’s Hospital), Shenzhen Key Laboratory of Musculoskeletal Tissue Reconstruction and Function Restoration, Shenzhen, 518020 China

**Keywords:** Vascular calcification, Intestinal microbiota, Short-chain fatty acids, Propionate, *Akkermansia muciniphila*

## Abstract

**Background:**

Vascular calcification is a major cause of the high morbidity and mortality of cardiovascular diseases and is closely associated with the intestinal microbiota. Short-chain fatty acids (SCFAs) are derived from the intestinal microbiota and can also regulate intestinal microbiota homeostasis. However, it remains unclear whether exogenous supplementation with propionate, a SCFA, can ameliorate vascular calcification by regulating the intestinal microbiota. This study was conducted to explore the roles of propionate and the intestinal microbiota in the process of vascular calcification.

**Methods:**

In total, 92 patients were enrolled consecutively as the observational cohort to analyse the relationship between SCFAs and vascular calcification in both blood and faecal samples. A rat model of vascular calcification was induced by vitamin D3 and nicotine (VDN) to validate the effect of propionate. Differences in the intestinal microbiota were analysed by 16S ribosomal RNA gene sequencing. Faecal microbiota transplantation and *Akkermansia muciniphila* transplantation experiments were performed to evaluate the functions of the intestinal microbiota.

**Results:**

The results of the observational cohort study revealed that the levels of SCFAs (particularly propionate) in both blood and faecal samples independently correlated negatively with calcification scores (*P* < 0.01). To verify the activities of propionate, it was provided to VDN-treated rats, and oral or rectal propionate delivery reshaped the intestinal microbiota, resulted in elevated SCFA production, improved intestinal barrier function and alleviated inflammation, ultimately ameliorating vascular calcification. Furthermore, we demonstrated that transplantation of the propionate-modulated intestinal microbiota induced beneficial outcomes similar to those with oral or rectal propionate administration. Interestingly, linear discriminant analysis (LDA) effect size (LEfSe) revealed that oral or rectal propionate administration and propionate-modulated intestinal microbiota transplantation both enriched primarily *Akkermansia*. Subsequently, we demonstrated that *Akkermansia* supplementation could ameliorate VDN-induced vascular calcification in rats.

**Conclusions:**

Propionate can significantly ameliorate vascular calcification in VDN-treated rats, and this effect is mediated by intestinal microbiota remodelling. The findings in our study indicate that the intestinal tract-vessel axis is a promising target for alleviating vascular calcification.

Video Abstract

**Supplementary Information:**

The online version contains supplementary material available at 10.1186/s40168-022-01390-0.

## Introduction

Vascular calcification is a common pathological phenotype characterised by ectopic hydroxyapatite mineral deposition in the vascular wall, and its pathogenesis has been closely associated with inflammation [1]. Among the general population aged 45–75 years old, the prevalence of thoracic aortic calcification and coronary artery calcification has reached 63.1 and 46.7%, respectively [2]. Vascular calcification has a strong correlation with ageing, chronic kidney disease, diabetes, hypertension and smoking [3, 4]. It can induce vascular compliance decrease, arterial wall thickening, luminal stenosis, plaque instability and plaque rupture [5], which may result in a series of cardiovascular diseases as well as adverse cardiovascular and cerebrovascular events [6, 7]. Hence, vascular calcification is deemed a major cause of the high morbidity and mortality of cardiovascular diseases [4, 8]. With the progression of vascular calcification being irreversible in nature and the absence of available effective therapeutic treatments, its prevention and treatment are of vital clinical significance.

Short-chain fatty acids (SCFAs), primarily including acetic acids, propionic acids and butyric acids, are metabolites produced by dietary fibre fermentation via specific anaerobic bacteria in the colon [9, 10]. In recent years, SCFAs have been reported to be able to improve cardio-metabolic disease-associated risk factors, which have attracted increasing awareness [11]. Accordingly, treatment with SCFAs can control weight gain by regulating caloric intake and energy consumption [12], improve blood glucose homeostasis and insulin resistance [13, 14], lower plasma cholesterol levels [15] and promote fatty acid oxidation [16]. Moreover, the improvement of these recognised risk factors can benefit the control of the occurrence and progression of cardiovascular diseases. In addition, SCFAs can have a profound beneficial impact on the cardiovascular system owing to their anti-inflammation and immunity homeostasis regulatory functions. SCFAs can reduce blood pressure by improving vascular compliance [17–19]. Additionally, recent studies have found that supplementation with lactobacillus probiotics regulates the immune response and then promotes repair after myocardial infarction. Further studies have shown that after probiotic supplementation, the total SCFA content remained constant but the metabolite composition varied in serum and faecal samples. Propionate levels were significantly increased, while butyrate levels were markedly decreased. The number of myeloid cells in the myocardium increased significantly after propionate supplementation. All these findings suggested that the intestinal microbiota produced specific SCFAs that regulated the immune system and then the post-myocardial infarction inflammatory microenvironment [20]. In addition, Bartolomaeus et al. found that propionate reduced the systemic inflammatory response, atherosclerosis and myocardial remodelling caused by hypertension through balancing Treg cell responses [21]. Therefore, it is presumed that the SCFA propionate can relieve vascular calcification by exerting an anti-inflammatory function.

SCFAs are derived from the intestinal microbiota and can also regulate intestinal microbiota homeostasis [22]. The intestinal microbiota is an essential structural component of the intestinal barrier. A healthy intestinal microbiota is beneficial for sustaining intact intestinal barrier function, and the intact intestinal barrier is vitally related to the health status of the host [23]. Intestinal microecological imbalance may impair the integrity of the intestinal barrier. Consequently, lipopolysaccharide (LPS) in the cell wall of gram-negative bacteria and harmful metabolites such as trimethylamine (TMA) can infiltrate the systemic circulation [24] and induce chronic inflammatory responses in vivo, which may consequently result in vascular endothelial dysfunction [25], atherosclerosis [26], vascular calcification [27], heart failure [28] and hypertension [29].

Considering the close association of SCFAs, the intestinal microbiota and inflammation interacting with vascular calcification, our study proposed a hypothesis that the SCFA propionate could alleviate vascular calcification through the improvement of intestinal barrier function and weakening of inflammatory responses by means of intestinal microbiota remodelling.

## Results

### Correlation analysis of the levels of SCFAs (acetate, propionate and butyrate) with calcification scores

**Fig. 1:**
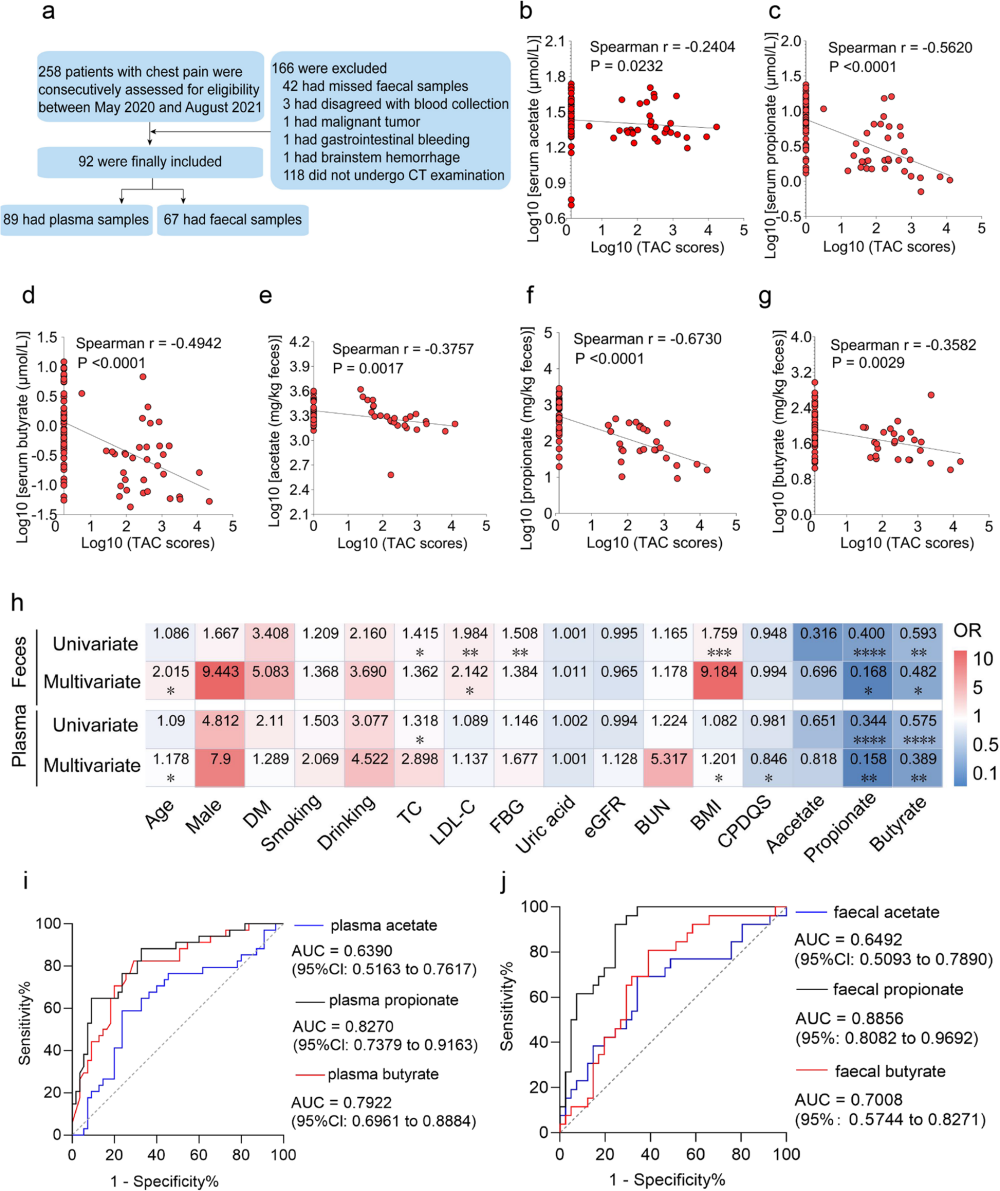
Correlation analysis of the levels of SCFAs (acetate, propionate and butyrate) with calcification scores. **a** A flow diagram of the enrolled research subjects. **b–d** Spearman’s correlation analyses of acetate, propionate and butyrate levels in plasma with calcification scores, respectively. **e–g** Spearman’s correlation analyses of acetate, propionate and butyrate levels in faeces with calcification scores, respectively. **h** Univariate and multivariate regression models were applied to analyse the correlations of acetate, propionate and butyrate levels in plasma and faeces with calcification scores. Blue: odds ratio (OR) <1.00 and negative correlation; Red: OR > 1.00 and positive correlation; *P < 0.05, **P < 0.01, ***P < 0.001, ****P < 0.0001. **i, j** Predictive ability of acetate, propionate and butyrate levels in plasma and faeces for vascular calcification, respectively

A total of 92 patients were enrolled in this study, yielding 89 blood samples and 67 faecal samples (Fig. 1a). The basic demographics and clinical characteristics of the participants that provided plasma and faecal samples are shown in Additional file 1: Supplementary Table 1 and Additional file 2: Supplementary Table 2, respectively. Spearman’s correlation analysis was performed, and acetate, propionate and butyrate levels in both plasma and faecal samples were negatively correlated with calcification scores (Fig. 1b–g). After further adjustment for confounding factors, multivariate logistic regression analysis revealed that the levels of SCFAs in plasma and faeces, especially propionate and butyrate, had independent negative correlations with the calcification score (Fig. 1h). This result suggests that the SCFAs (i.e. propionate and butyrate) are independent protective factors that suppress vascular calcification. Subsequently, receiver operating characteristic (ROC) curves were plotted to calculate the area under the curve (AUC) to assess the predictive role of SCFAs for vascular calcification. It was discovered that the largest AUC was generated by propionate in plasma and faecal samples, followed by butyrate, and acetate had the lowest AUC. In particular, the AUC of propionate in plasma and faeces was calculated to be 0.8270 (95% CI 0.7379 to 0.9163) and 0.8856 (95% CI 0.8082 to 0.9692), respectively (Fig. 1i, j). Additionally, we also analysed the correlation between SCFAs and other clinical indicators and found that SCFAs in plasma and faecal samples were negatively correlated with total cholesterol (TC), low-density lipoprotein-cholesterol (LDL-C), fasting blood glucose (FBG) and body-mass index (BMI) to varying degrees and positively correlated with the China Prime Diet Quality Score (CPDQS) (Additional file 3: Supplementary Table 3 and Additional file 4: Supplementary Table 4). These results suggest that diet has a certain effect on SCFA levels.

### Amelioration of VND-induced rat vascular calcification and reduction in inflammatory responses by oral and rectal propionate administration

**Fig. 2:**
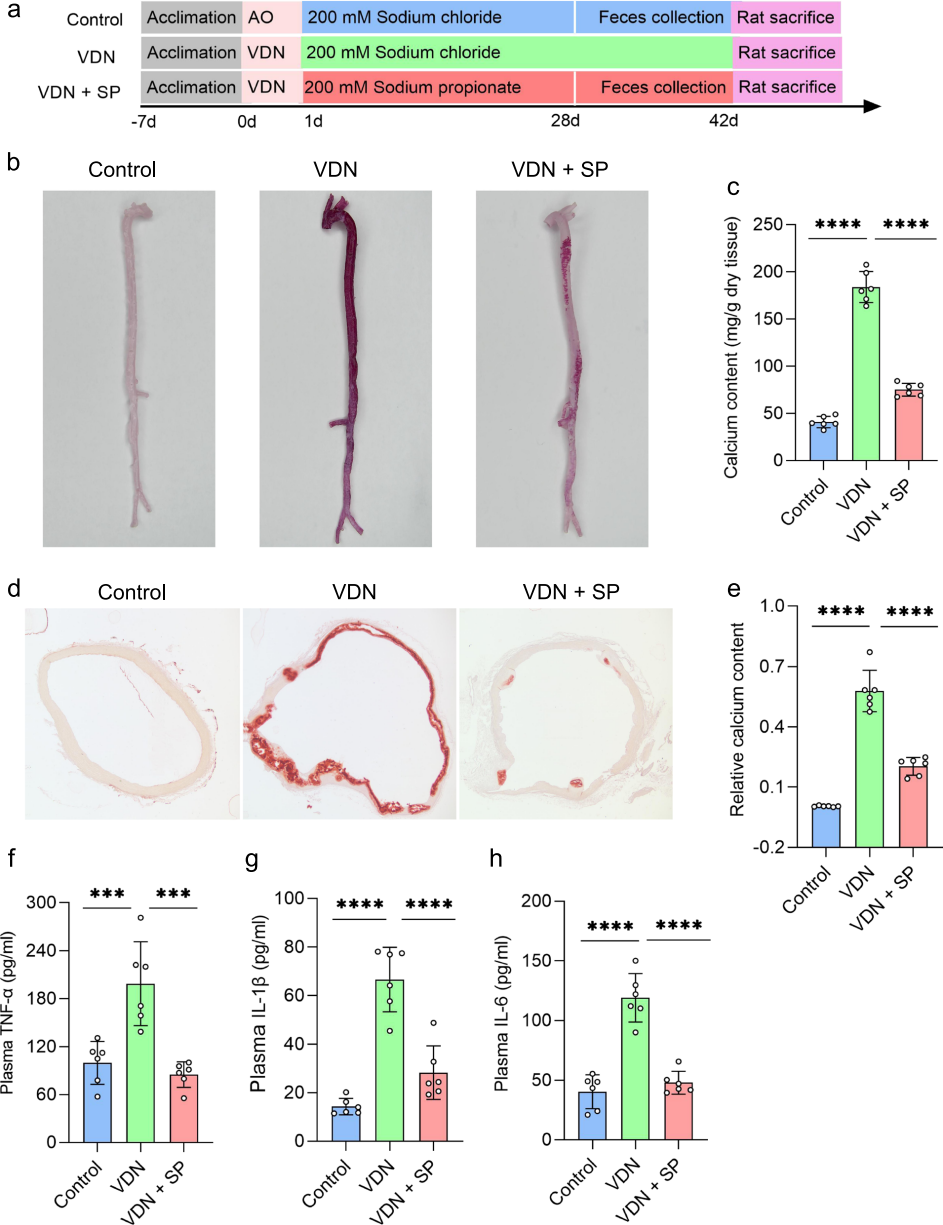
Improvements in VND-induced rat vascular calcification and reduction in inflammation by oral propionate administration. **a** A flow diagram of the oral propionate administration experiment. For 6 consecutive weeks, rats in the control and VDN groups were provided free access to sodium chloride solution (200 mM), while rats from the VDN + SP group freely consumed sodium propionate solution (200 mM). **b** Macroscopic observation of arterial vascular calcification based on alizarin red staining. **c** Quantitative evaluation of calcium content in whole aortas. **d** Observation of vascular calcification of the ascending aorta through alizarin red staining of tissue sections (original magnification ×40). **e** Relative quantification of calcium content for the vessel sections of the ascending aorta. **f–h** Concentrations of the proinflammatory cytokines TNF-α, IL-1β and IL-6 in plasma, respectively. AO: pure alcohol and peanut oil; VDN: vitamin D3 and nicotine; SP: sodium propionate. Data are presented as the mean ± standard deviation (SD). Statistical significance was determined using one-way ANOVA (Tukey post hoc test). ***P<0.001, ****P<0.0001

To clarify the effect of sodium propionate (SP) on vascular calcification, vascular calcification was induced in rats, followed by free drinking of SP (200 mM) (Fig. 2a) or rectal delivery of 1 g/kg body weight SP (Additional file 5: Supplementary Figure 1a). The experiment lasted for 6 weeks. There was no significant difference in serum creatinine levels among the three groups (Additional file 6: Supplementary Figure 2b), excluding the influence of vitamin D3 on renal functions of rats. Compared with that in the VDN group rats, calcium salt deposition obviously dropped in the blood vessels of rats from the VDN + SP group (Fig. 2b). Additionally, quantification of aortic calcium content showed that calcium content was significantly decreased by 59.2% in the VDN + SP group compared with that in the VDN group (Fig. 2c). Similarly, alizarin red staining of ascending aorta sections showed an apparently suppressed deposition of calcium salt in blood vessels of rats from the VDN + SP group (Fig. 2d). Furthermore, relative quantification of calcium salt also revealed that the calcium content in tissue sections in the VDN + SP group decreased by 64.8% (Fig. 2e) when compared with that in the VDN group. Plasma proinflammatory cytokine levels were measured to further evaluate the impacts of SP on systemic inflammatory responses. Compared with the VDN group, the VDN + SP group demonstrated significant decreases in TNF-α (Fig. 2f), IL-1β (Fig. 2g) and IL-6 (Fig. 2h) concentrations in plasma. Moreover, macrophage infiltration in the vessel wall was attenuated, and the expression of TNF-α was reduced (Additional file 7: Supplementary Figure 3a-c). Likewise, rectal administration of propionate improved the above indicators (Additional file 5: Supplementary Figure 1b-h and Additional file 8: Supplementary Figure 4a-c). Conclusively, all these results indicate that propionate not only ameliorates VDN-induced vascular calcification but also reduces inflammatory responses.

### Oral and rectal propionate administration regulated the composition and SCFA production of the gut microbiota

**Fig. 3:**
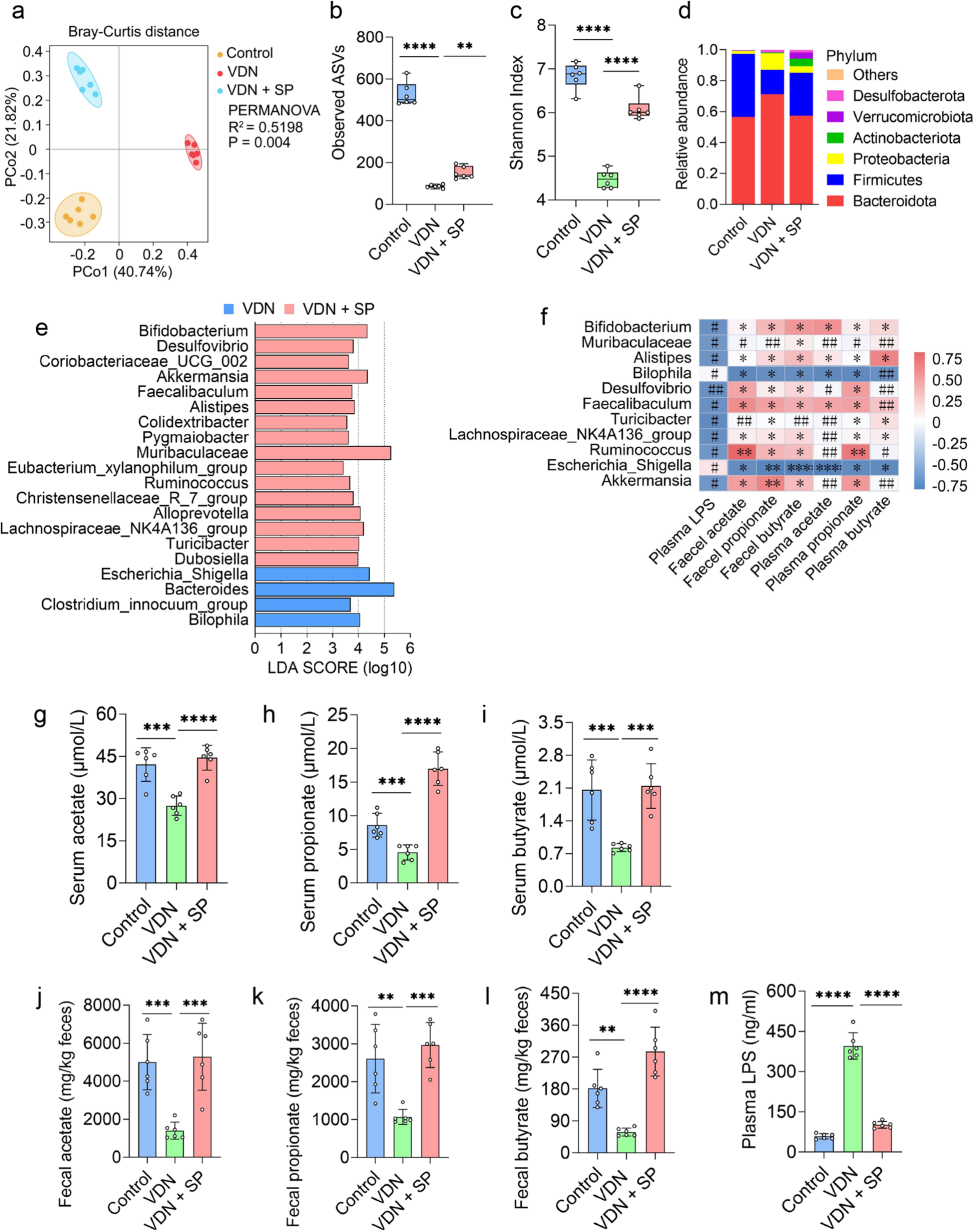
Amelioration of intestinal microbiota imbalance in rats by oral propionate administration. **a** Principal coordinate analysis (PCoA) diagram showing the β-diversity of intestinal microbiota among the three groups. **b**,** c** α-diversity of the intestinal microbiota. **d** Relative abundance of intestinal microbiota constituents at the phylum level. **e** Analysis of the differences in the intestinal microbiota by LEfSe. **f** Spearman’s correlation analysis of the relationship of the intestinal microbiota with LPS and SCFAs. Negative and positive correlations are denoted in blue and red, respectively. **g–i** Acetate, propionate and butyrate levels in plasma, respectively. **j–l** Acetate, propionate and butyrate concentrations in faeces, respectively. **m** Plasma LPS levels. Data are presented as the mean ± standard deviation (SD). Statistical significance was determined using one-way ANOVA or the Kruskal–Wallis test (Tukey post hoc test). NS for *P* > 0.05, #*P* < 0.25, ##*P* < 0.1, *P< 0.05, **P< 0.01, ***P< 0.001, ****P< 0.0001

SCFAs are major media for interactions between the intestinal microbiota and the host. SCFAs are derived from the intestinal microbiota and can also regulate intestinal microbiota homeostasis [22]. However, further confirmation is required to determine whether exogenous propionate supplementation can also regulate intestinal microbiota homeostasis. To explore this issue, 16S ribosomal RNA (16S rRNA) gene sequencing was performed to analyse the effect of oral and rectal propionate administration on the composition and abundance of the intestinal microbiota. As anticipated, principal coordinate analysis (PCoA) at the bacterial phylum level indicated that there were structural differences in the intestinal microbiota among the three groups (*R*^2^ = 0.5198; *P* = 0.004) (Fig. 3a and Additional file 9: Supplementary Figure 5a). Further analysis of the differences in each bacterial phylum structure among the three groups was performed (Additional file 10: Supplementary Table 5 and Additional file 11: Supplementary Table 6). The α-diversity of the intestinal microbiota in VDN-treated rats significantly declined (Fig. 3b, c). In the VDN group, *Bacteroidetes*, *Firmicutes* and *Proteobacteria* were predominant in the gut microbiota at the bacterial phylum level (Fig. 3d and Additional file 9: Supplementary Figure 5d), and at the bacterial genus level, *Bacteroides* (Additional file 6: Supplementary Figure 2a) and *Escherichia_Shigella* (Additional file 12: Supplementary Figure 6a) were dominant. Abundance analysis at the phylum and genus levels showed that the *Firmicutes/Bacteroidetes* ratio and the *Firmicutes*, *Muribaculaceae* and *Alloprevotella* abundance (Additional file 6: Supplementary Figure 2c-f) in the VDN group were significantly decreased compared to those in the control group; the *Bacteroidota*, *Proteobacteria*, *Bacteroides*, *Bilophila* and *Escherichia_Shigella* abundance (Additional file 6: Supplementary Figure 2g-k) was significantly increased. Apparently, oral propionate administration reversed the above trends (Fig. 3b, c and Additional file 6: Supplementary Figure 2c-k), and *Actinobacteria, Akkermansia, Dubosiella, Verrucomicrobia, Desulfovibrio, Bifidobacterium* and *Turicibacter* (Additional file 6: Supplementary Figure 2l-r) were significantly enriched. Similarly, rectal propionate administration also improved the α-diversity (Additional file 9: Supplementary Figure 5b-c) and intestinal microbiota abundance (Additional file 12: Supplementary Figure 6b-j). LEfSe showed that *Akkermansia* and *Muribaculaceae* were coenriched in the VDN + SP group (Fig. 3e) and VDN + rectal-SP group (Additional file 9: Supplementary Figure 5e) (LDA > 2).

Furthermore, Spearman correlation analysis indicated that *Muribaculaceae* and *Akkermansia* were positively correlated with SCFAs in plasma and faeces to different degrees and exhibited a negative correlation with plasma LPS. Moreover, *Escherichia_Shigella*, *Bilophila* and *Parasutterella* had a negative correlation with SCFAs in plasma and faeces to a varied extent and had a positive correlation with plasma LPS (Fig. 3f and Additional file 9: Supplementary Figure 5f).

To further investigate the impacts of oral and rectal propionate administration on SCFA and LPS levels, we measured plasma LPS content, as well as the concentrations of acetate, propionate and butyrate, in plasma and faeces. The concentrations of acetate, propionate and butyrate (Fig. 3g–l and Additional file 9: Supplementary Figure 5g-l) were reduced to different extents in the VDN group, and LPS (Fig. 3m and Additional file 9: Supplementary Figure 5m) was significantly enriched. However, oral and rectal propionate administration enormously reduced plasma LPS levels and increased acetate, propionate and butyrate production in plasma and faeces (Fig. 3g–m and Additional file 9: Supplementary Figure 5g-m).

Overall, oral and rectal propionate administration ameliorated VDN-induced vascular calcification in rats and promoted SCFA production, suggesting that the gut microbiota might play a vital role in alleviating VDN-induced vascular calcification in rats.

### Alleviation of intestinal mucosal barrier impairment in rats by oral and rectal propionate administration

**Fig. 4:**
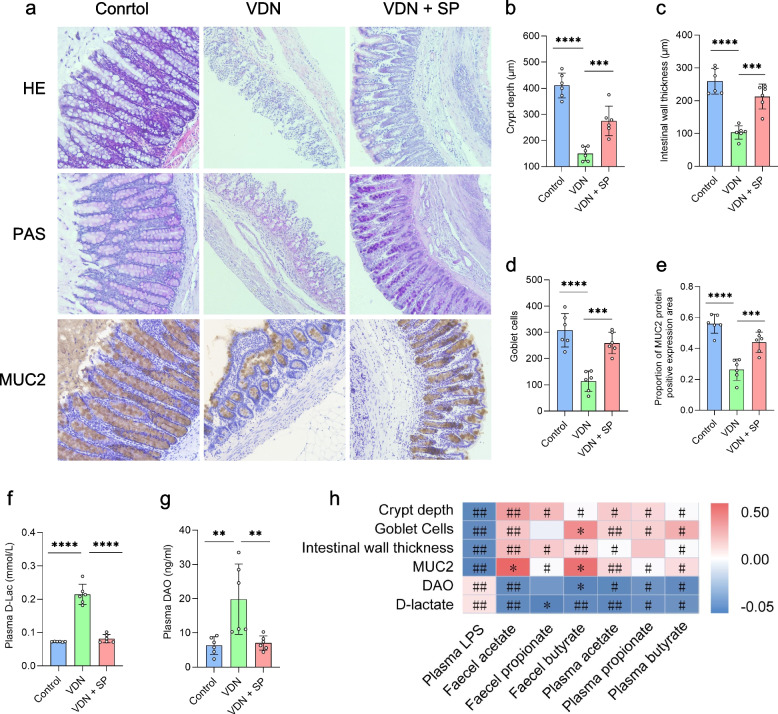
Alleviation of intestinal mucosal barrier impairment in rats by oral propionate administration. **a** Typical haematoxylin-eosin (HE) staining, periodic acid-Schiff (PAS) staining and MUC2 immunohistochemical staining for intestinal tissues (original magnification ×200). **b–e** Parameters of crypt depth, intestinal wall thickness, goblet cell count and MUC2 expression levels, respectively. **f**,** g** Plasma D-lactate and diamine oxidase contents, respectively. **h** Spearman’s correlation analysis of the relationship of SCFAs and LPS with intestinal barrier-related parameters (e.g. crypt depth, intestinal wall thickness, MUC2 level and goblet cell count). Red and blue denote positive and negative correlations, respectively. Data are presented as the mean±standard deviation (SD). Statistical significance was determined using one-way ANOVA (Tukey post hoc test). #*P* < 0.25, ##*P* < 0.1, *P< 0.05, **P< 0.01, ***P< 0.001, ****P< 0.0001

A healthy intestinal microbiota provides a structural basis for sustaining intestinal barrier function. An intact intestinal barrier not only prevents harmful substances in the enteric cavity from infiltrating into the body but also serves as a critical foundation for maintaining in vivo homeostasis. In our experiment, we analysed colon histology to explore the influence of oral and rectal propionate administration on the intestinal barrier integrity of VDN-treated rats. In comparison to the control group, the VDN group showed critically damaged colonic crypts in rats (Fig. 4a, b), thinning of the intestinal wall (Fig. 4a, c) and a dramatic reduction in the number of goblet cells (Fig. 4a, d). Mucus protein 2 (MUC2) is secreted by goblet cells and is the most abundantly expressed mucin in the mucus layer. Immunohistochemical analysis revealed that the quantity of MUC2-positive goblet cells was substantially reduced in the VDN group in comparison to that in the control group (Fig. 4a, e). A significant accumulation of D-lactate and diamine oxidase, biomarkers of intestinal leakage (Fig. 4f, g), was observed, indicating a tremendous enhancement in intestinal permeability in rats from the VDN 1 group. After treatment with SP, there was a clear improvement in the structure of the intestinal barrier, such as crypt depth (Fig. 4a, b), intestinal wall thickness (Fig. 4a, c), the number of goblet cells (Fig. 4a, d) and MUC2 accumulation (Fig. 4a, e). Simultaneously, the permeability of the intestinal tract increased as well (Fig. 4f, g). Correlation analysis revealed that intestinal barrier-associated parameters (e.g. crypt depth, intestinal wall thickness, goblet cell count and MUC2 accumulation) were negatively correlated with LPS content, positively correlated with SCFA levels, and negatively correlated with D-lactate and diamine oxidase levels (Fig. 4h). Similarly, intestinal mucosal parameters and permeability parameters in rats with VDN-induced vascular calcification were improved by rectal propionate administration (Additional file 13: Supplementary Figure 7a-h). In brief, these results indicate that oral and rectal propionate administration may play a role in protecting the intestinal mucosal barrier.

In conclusion, all results described above signify that oral and rectal propionate administration can alleviate VDN-induced vascular calcification in rats, which may occur via the remodelling of the intestinal microbiota that increases SCFA production, improves intestinal barrier function and alleviates inflammation.

### Suppression of VDN-induced vascular calcification and inflammatory responses in rats by the propionate-modulated intestinal microbiota

**Fig. 5:**
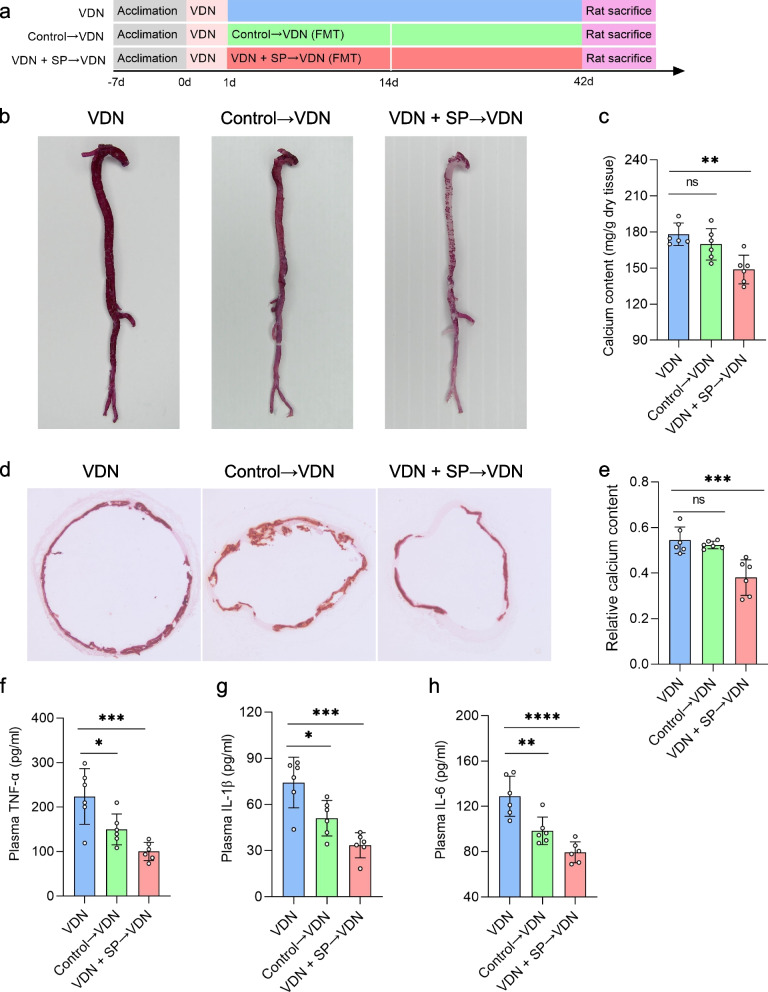
Alleviation of VDN-induced vascular calcification in rats by the propionate-modulated intestinal microbiota. **a** A flow diagram of the faecal microbiota transplantation experiment. Rats in the control and VDN + SP groups were raised for 28 days. Following this, faecal samples were collected from these donors on a daily basis. Based on procedures described in the methodology section, a bacterial solution was prepared and then transplanted continually into the recipient rats for 14 days. The recipients were separately assigned to the control→VDN and VDN + SP→VDN groups. **b** Macroscopic observation of arterial vascular calcification through alizarin red staining. **c** Quantitative assessment of calcium content in macroscopic blood vessels. **d** Observation of vascular calcification of the ascending aorta by alizarin red staining of tissue sections (original magnification ×40). **e** Relative quantification of calcium content for the sections of ascending aortas. **f–h** Concentrations of the proinflammatory cytokines TNF-α, IL-1β and IL-6 in plasma, respectively. VDN: Vitamin D3 and nicotine; FMT: faecal microbiota transplantation. Data are presented as the mean ± standard deviation (SD). Statistical significance was determined using one-way ANOVA (Tukey post hoc test). #*P* < 0.25, ##*P* < 0.1, *P< 0.05, **P< 0.01, ***P< 0.001, ****P< 0.0001

We next validated the effect of the propionate-modulated microbiota on vascular calcification in rats by transplanting the faecal microbiota of rats from the control and VDN + SP groups into VDN-treated rats (Fig. 5a). Six weeks later, calcium salt deposition in the aortic arteries of rats from the VDN + SP→VDN group was evidently lower than that in the aortic arteries of rats from the VDN group (Fig. 5b). In addition, quantification of calcium content revealed that calcium content in the VDN + SP→VDN group was decreased by 16.5% (Fig. 5c) compared with that in the VDN group. Similarly, alizarin red staining of ascending aorta sections showed a dramatically inhibited deposition of calcium salt in aortic arteries of rats in the VDN + SP→VDN group (Fig. 5d). Furthermore, in the VDN + SP→VDN group, the calcium content was decreased by 30.1% (Fig. 5e) compared with that in the VDN group, and there was a significant reduction in the concentrations of TNF-α (Fig. 5f), IL-1β (Fig. 5g) and IL-6 (Fig. 5h) in plasma. Moreover, immunofluorescence analysis demonstrated attenuated macrophage infiltration in the vessel wall and diminished expression of TNF-α (Additional file 14: Supplementary Figure 8a-c). Compared with those in the VDN group, TNF-α (Fig. 5f), IL-1β (Fig. 5g) and IL-6 (Fig. 5h) concentrations in plasma were reduced to varying degrees in the control→VDN group; however, there was no significant impact on the suppression of vascular calcification and inflammation in the vascular wall (Fig. 5b–e and Additional file 14: Supplementary Figure 8a-c). Collectively, these results suggest that the propionate-modulated intestinal microbiota could relieve VDN-induced vascular calcification in rats and reduce inflammation in vivo.

### Intestinal microecological imbalance improvement in VDN-treated rats by the propionate-modulated intestinal microbiota

**Fig. 6:**
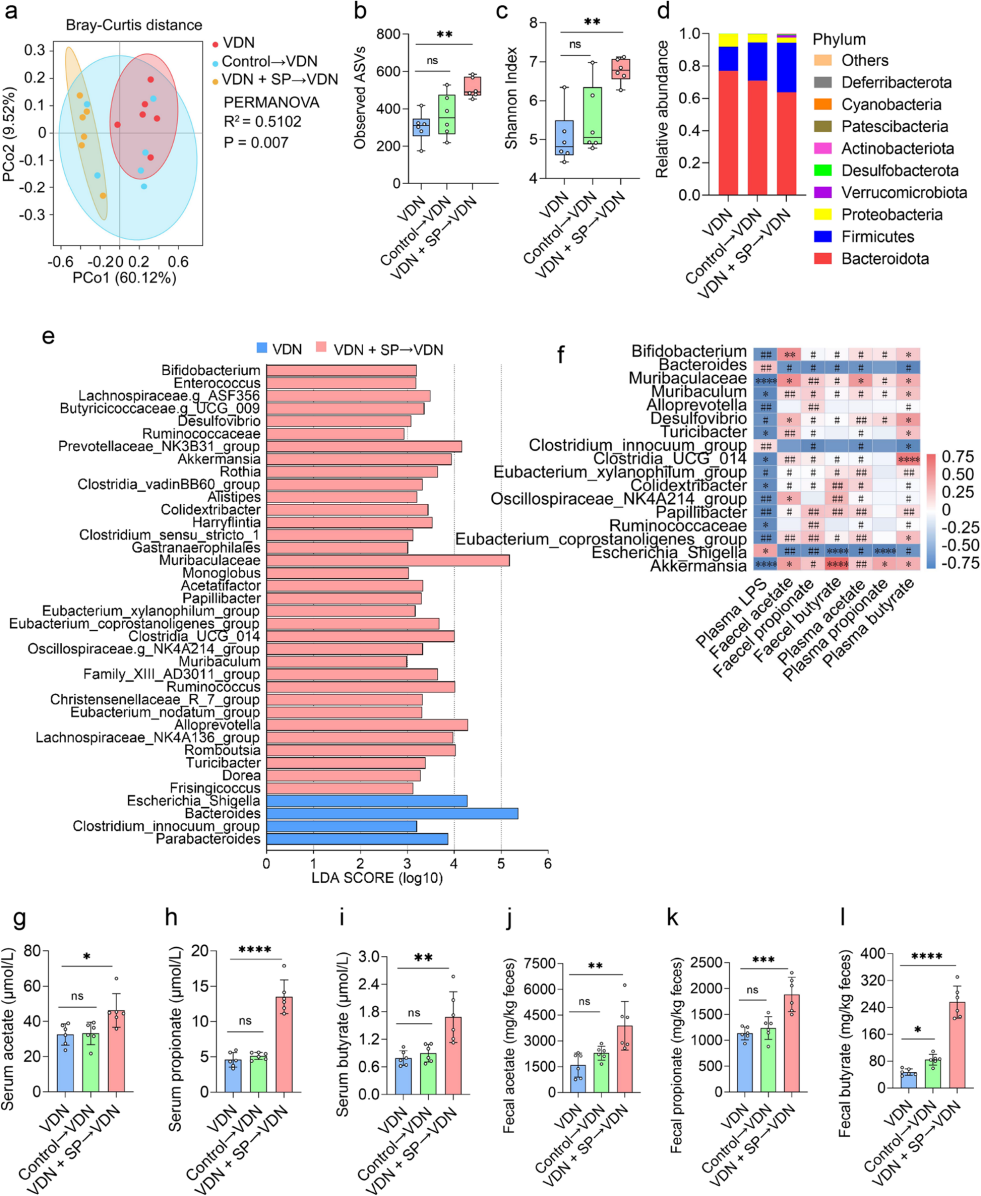
Intestinal microecological imbalance improvement in VDN-treated rats by the propionate-modulated intestinal microbiota. **a** PCoA diagram showing the β-diversity of the intestinal microbiota among the three groups. **b**, **c** α-diversity of the intestinal microbiota. **d** Relative abundance of intestinal microbiota constituents at the phylum level. **e** Analysis of the differences in the intestinal microbiota by LEfSe. **f** Spearman’s correlation analysis of the relationship of the intestinal microbiota with LPS and SCFAs. Negative and positive correlations are denoted in blue and red, respectively. **g–i** Acetate, propionate and butyrate levels in plasma, respectively. **j–l** Acetate, propionate and butyrate concentrations in faeces, respectively. Data are presented as the mean ± standard deviation (SD). Statistical significance was determined using one-way ANOVA (Tukey post hoc test). #*P* < 0.25, ##*P* < 0.1, *P< 0.05, **P< 0.01, ***P< 0.001, ****P< 0.0001

On the 6th week after faecal microbiota transplantation, 16S rRNA gene sequencing was carried out to analyse the composition of the intestinal microbiota. As revealed by PCoA at the bacterial phylum level, there was a difference in the structures of the intestinal microbiota among the three groups (*R*^2^ = 0.5102; *P* = 0.007) (Fig. 6a). While PERMANOVA revealed that no obvious structural change in the intestinal microbiota between the control→VDN group and the VDN group (*R*^2^ = 0.2285, *P* = 0.118), there was an obvious alteration in the structures of the intestinal microbiota between the VDN + SP→VDN group and the VDN group (*R*^2^ = 0.6965, *P* = 0.004) (Additional file 15: Supplementary Table 7). Moreover, we further analysed the differences in the bacterial phylum structure among the three groups (Additional file 15: Supplementary Table 7). In addition, compared with the VDN group, the control→VDN group did not exhibit a significantly difference in intestinal microbiota α-diversity (Fig. 6b, c) or abundance at the phylum and genus levels (Additional file 16: Supplementary Figure 9c-v). The main intestinal microbiota composition at both the genus and phylum levels was similar among the groups (Fig. 6d and Additional file 16: Supplementary Figure 9a). Upon FMT intervention, the VDN + SP→VDN group exhibited substantially improved intestinal microbiota α-diversity (Fig. 6b, c). Moreover, at the phylum and genus levels, the relative abundance was tremendously altered, including increases in the ratio of *Firmicutes/Bacteroidetes* abundance as well as the abundances of *Firmicutes*, *Verrucomicrobiota*, *Akkermansia*, *Bifidobacterium*, etc. (Additional file 16: Supplementary Figure 9c-q) and decreases in the abundances of *Bacteroidetes*, *Proteobacteria*, *Escherichia_Shigella*, etc. (Additional file 16: Supplementary Figure 9r-v). Additionally, LEfSe showed that 34 bacterial genera, including *Akkermansia*, were enriched in the VDN + SP→VDN group, while four taxa were enriched in the VDN group (LDA > 2) (Fig. 6e).

Furthermore, Spearman correlation analysis indicated that the abundances of *Bifidobacterium*, *Muribaculaceae*, *Akkermansia*, etc., were positively correlated with the levels of SCFAs in both plasma and faeces and negatively correlated with plasma LPS content. *Bacteroidetes*, *Clostridium_innocuum_group* and *Escherichia_Shigella* had a negative correlation with SCFAs to different degrees in plasma and faeces and a positive correlation with plasma LPS (Fig. 6f).

Furthermore, we further measured LPS content in plasma as well as acetate, propionate and butyrate concentrations in both plasma and faeces. The control→VDN group showed no apparent decrease in LPS content (Additional file 16: Supplementary Figure 9b) and no evident increase in acetate, propionate or butyrate concentration (excluding butyrate in faeces) (Fig. 6g–l). While the VDN + SP→VDN group showed a significant reduction in LPS content (Additional file 16: Supplementary Figure 9b), the accumulation of acetate, propionate and butyrate increased (Fig. 6g–l).

Overall, these results demonstrated that the propionate-modulated intestinal microbiota regulated the gut microbiota composition and its metabolism, leading to the potential to mitigate VDN-induced dysbiosis.

### Alleviation of intestinal mucosal barrier impairment in VDN-treated rats by the propionate-modulated intestinal microbiota

**Fig. 7:**
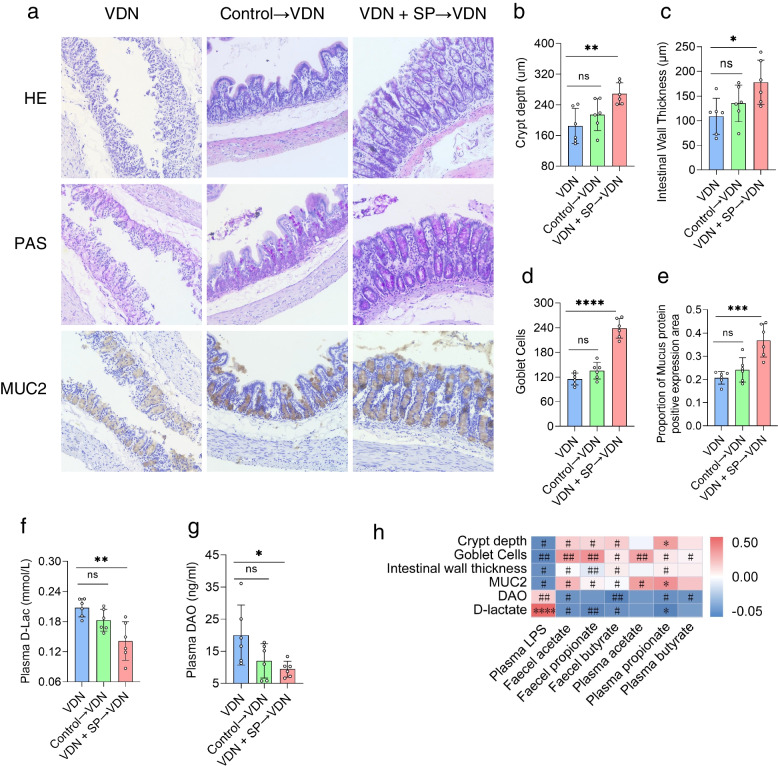
Alleviation of intestinal mucosal barrier impairment in VDN-treated rats by the propionate-modulated intestinal microbiota. **a** Typical haematoxylin-eosin (HE) staining, periodic acid-Schiff (PAS) staining and MUC2 immunohistochemical staining for intestinal tissues (original magnification ×200). **b–e** Parameters of crypt depth, intestinal wall thickness, goblet cell count and MUC2 expression levels, respectively. **f**, **g** Plasma D-lactate and diamine oxidase contents, respectively. **h** Spearman’s correlation analysis of the relationship of SCFAs and LPS with intestinal barrier-related parameters (e.g. crypt depth, intestinal wall thickness, MUC2 level and goblet cell count). Red and blue denote positive and negative correlations, respectively. Data are presented as the mean±standard deviation (SD). Statistical significance was determined using one-way ANOVA (Tukey post hoc test). #*P* < 0.25, ##*P* < 0.1, *P< 0.05, **P< 0.01, ***P< 0.001, ****P< 0.0001

As described above, the histological characteristics of rat colons were analysed to investigate the impact of the propionate-modulated intestinal microbiota on the integrity of the intestinal barrier in VDN-treated rats. Compared with that in rats from the VDN group, the integrity of intestinal epithelium was improved in rats from the VDN + SP→VDN group, including increased crypt depth (Fig. 7a, b), intestinal wall thickening (Fig. 7a, c), an increased goblet cell count (Fig. 7a, d), elevated MUC2 expression (Fig. 7a, e) and significantly decreased D-lactate and diamine oxidase content (Fig. 7f, g). For rats in the control→VDN group, there were no evident changes in intestinal barrier parameters (Fig. 7a–e) or permeability parameters (Fig. 7f, g). According to correlation analysis, intestinal barrier parameters (crypt depth, intestinal wall thickness, goblet cell count and MUC2 accumulation) were negatively correlated with LPS content but were positively correlated with SCFA levels. Moreover, D-lactate and diamine oxidase formed negative correlations with SCFAs (Fig. 7h). In summary, these results suggest that the propionate-modulated intestinal microbiota exerts a protective function in the intestinal mucosal barrier.

Overall, the propionate-modulated intestinal microbiota can alleviate calcium salt deposition in the vascular wall depending on intestinal microbiota remodelling, which can increase the diversity of the intestinal microbiota, promote the production of SCFAs, relieve the impairment of the intestinal barrier and mitigate inflammatory responses.

### Alleviation of VDN-induced vascular calcification in rats by Akkermansia

**Fig. 8:**
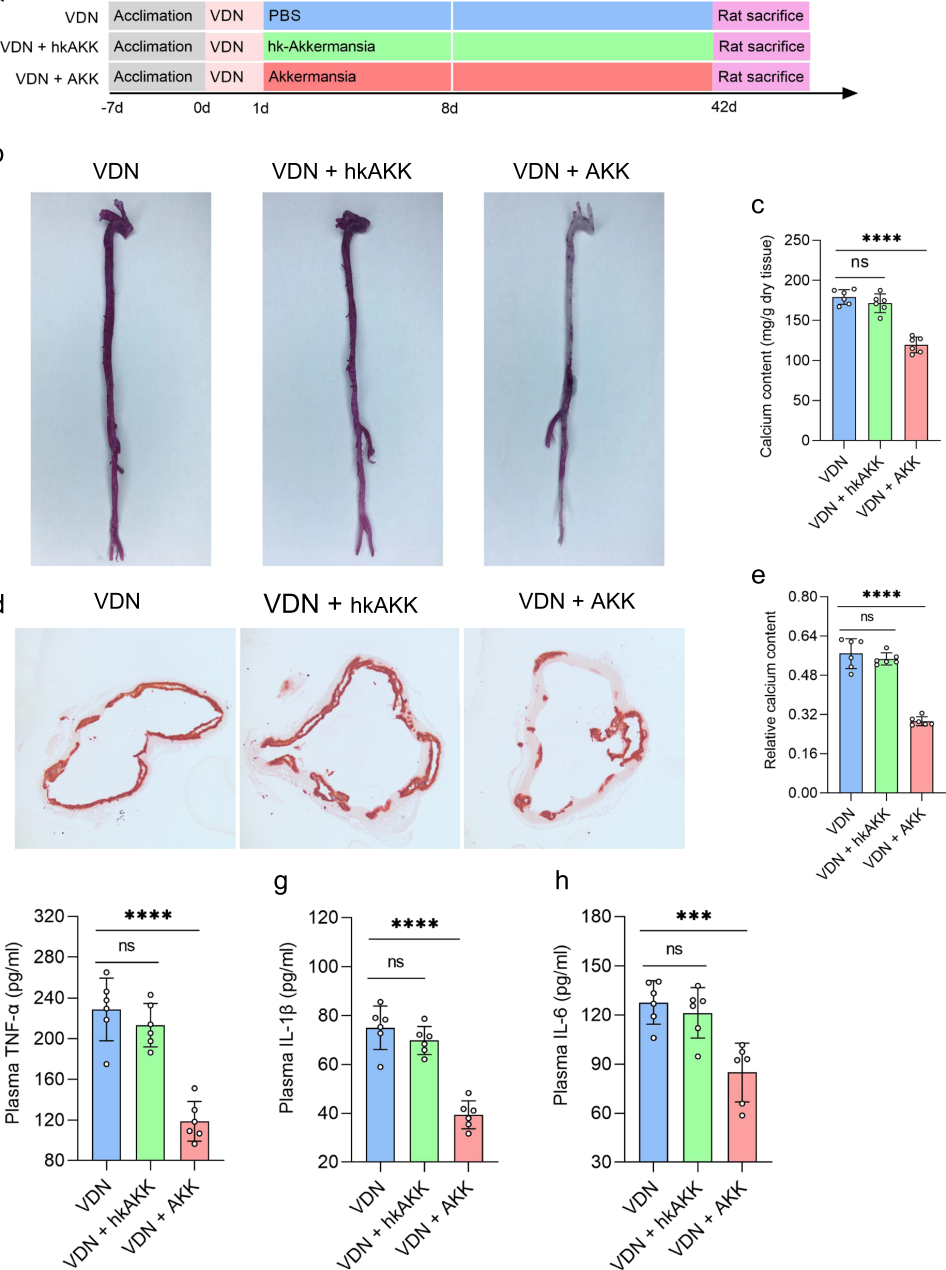
Alleviation of VDN-induced vascular calcification in rats by *Akkermansia.* **a** Flow diagram of the *Akkermansia* transplantation experiment. Rats from the VDN group were orally administered PBS solution, rats from the VDN + hk-AKK group were supplied with heat-killed *Akkermansia* via gavage, rats from the VDN + AKK group were intragastrically provided with active *Akkermansia*, and the treatment lasted for 8 consecutive days. **b** Macroscopic observation of aortic vascular calcification by alizarin red staining. **c** Quantitative evaluation of calcium content in macroscopic blood vessels. **d** Observation of ascending aorta vascular calcification based on alizarin red staining of tissue sections (original magnification ×40). **e** Calcium content in the vessel sections of the ascending aorta determined through relative quantification. **f–h** Plasma concentrations of the proinflammatory cytokines TNF-α, IL-1β and IL-6, respectively. hk-AKK: heat-killed *Akkermansia*; AKK: *Akkermansia*. Data are presented as the mean ± standard deviation (SD). Statistical significance was determined using one-way ANOVA (Tukey post hoc test). NS for *P* > 0.05, *P<0.05, **P<0.01, ***P<0.001, ****P<0.0001

Based on the above experiments, we found that *Muribaculaceae* and *Akkermansia* were coenriched in the VDN + SP group, the VDN + rectal-SP group and the VDN + SP VDN group (Additional file 17: Supplementary Figure 10a). Between them, *Akkermansia* was the largest contributor (Additional file 17: Supplementary Figure 10b). *Akkermansia* can reduce the level of metabolic endotoxins, alleviate inflammation and improve intestinal barrier function and has been recognised as a next-generation ‘probiotic’ candidate [30]. Subsequently, to observe whether vascular calcification can be relieved by *Akkermansia*, vascular calcification was induced in rats, followed by supplementation with *Akkermansia* (Fig. 8a). Interestingly, supplementation with *Akkermansia* mitigated vascular calcification (Fig. 8b–e) and significantly decreased the plasma concentrations of TNF-α (Fig. 8f), IL-1β (Fig. 8g) and IL-6 (Fig. 8h). Moreover, immunofluorescence analysis demonstrated attenuated macrophage infiltration in the vessel wall and diminished expression of TNF-α (Additional file 18: Supplementary Figure 11a-c). However, no similar effects were observed in rats administered heat-killed *Akkermansia* (Fig. 8b–h and Additional file 18: Supplementary Figure 11a-c).

### Improvement of intestinal microbiota imbalance in rats by Akkermansia

**Fig. 9:**
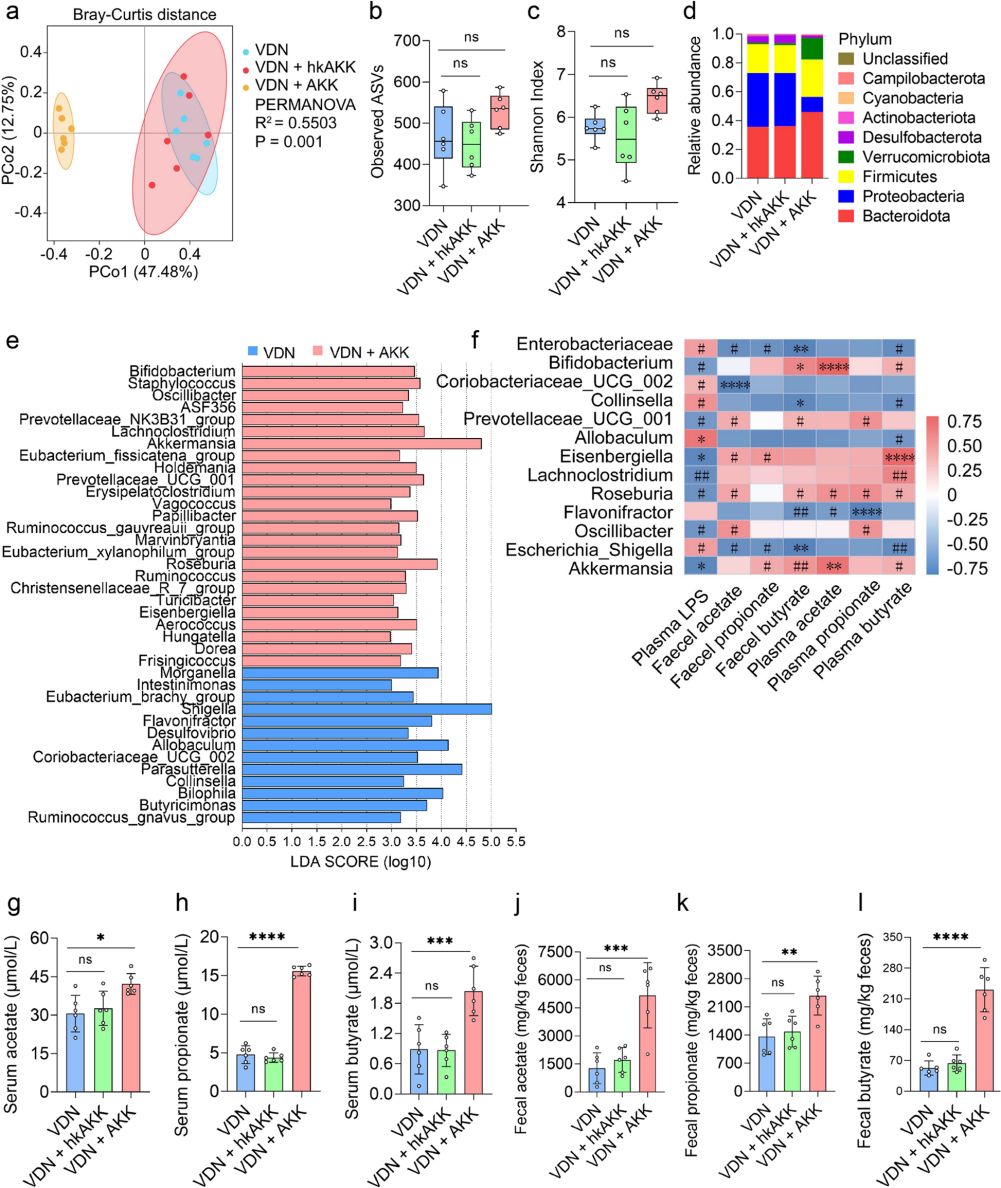
Amelioration of intestinal microbiota imbalance in rats by *Akkermansia.* **a** PCoA diagram showing the β-diversity of the intestinal microbiota among the three groups. **b**, **c** α-diversity of the intestinal microbiota. **d** Relative abundance of intestinal microbiota constituents at the phylum level. **e** Analysis of the differences in the intestinal microbiota by LEfSe. **f** Spearman’s correlation analysis of the relationship of the intestinal microbiota with LPS and SCFAs. Negative and positive correlations are denoted in blue and red, respectively. **g–i** Acetate, propionate and butyrate levels in plasma, respectively. **j–l** Acetate, propionate and butyrate concentrations in faeces, respectively. Data are presented as the mean ± standard deviation (SD). Statistical significance was determined using one-way ANOVA (Tukey post hoc test). #*P* < 0.25, ##*P* < 0.1, *P< 0.05, **P< 0.01, ***P< 0.001, ****P< 0.0001

Then, 16S rRNA gene sequencing was conducted to further explore the impact of *Akkermansia* on the composition of the intestinal microbiota in VDN-treated rats. Structural differences in the intestinal microbiota in the three groups were revealed by PCoA at the bacterial phylum level (*R*^2^ = 0.5503, *P* = 0.001) (Fig. 9a), despite nonsignificant changes in α-diversity (Fig. 9b, c). Moreover, there was also an apparent difference in microbial community structures at the phylum level (*Actinobacteriota*, *Desulfobacterota*, *Proteobacteria* and *Verrucomicrobiota*) (Additional file 19: Supplementary Table 8). Additionally, the changes in the gut microbiota composition of rats receiving *Akkermansia* (AKK) intervention at the phylum and genus levels (especially *Proteobacteria* and *Escherichia_Shigella*) were consistent with the above three trials (Fig. 9d and Additional file 17: Supplementary Figure 10c). According to LEfSe, the VDN + AKK group displayed enrichment of 25 bacterial genera, including *Akkermansia*, while 13 bacterial genera were enriched in the VDN group (Fig. 9e). Subsequently, abundance analysis at the phylum and genus levels showed that *Desulfobacterota*, *Proteobacteria*, *Escherichia_Shigella*, *Morganella*, etc. (Additional file 17: Supplementary Figure 10e-j), were significantly depleted, and *Akkermansia*, *Bifidobacterium*, *Lachnoclostridium*, etc. (Additional file 17: Supplementary Figure 10k-r), in the VDN + AKK group were significantly enriched compared to those in the VDN group, while there was no significant difference in the abundances of those taxa in the VDN + hk-AKK group (Additional file 17: Supplementary Figure 10e-r). Furthermore, Spearman correlation analysis demonstrated that *Bifidobacterium*, *Eisenbergiella*, *Akkermansia*, etc., were variably positively correlated with SCFAs in both plasma and faeces and negatively correlated with plasma LPS. *Enterobacteriaceae*, *Flavonifractor* and *Escherichia_Shigella* had a variably negative correlation with SCFAs in plasma and faeces and a positive correlation with plasma LPS (Fig. 9f).

LPS content in plasma and acetate, propionate and butyrate contents in both plasma and faeces were measured subsequently to explore the impacts of *Akkermansia* on both SCFA and LPS levels. LPS levels in plasma (Additional file 17: Supplementary Figure 10d) declined, whereas acetate, propionate and butyrate contents in both plasma and faeces (Fig. 9g–l) increased in the AKK group. However, no such evident change was found in the hk-AKK group (Fig. 9g–l and Additional file 17: Supplementary Figure 10d).

### Alleviation of intestinal mucosal barrier impairment in VDN-treated rats by Akkermansia

**Fig. 10:**
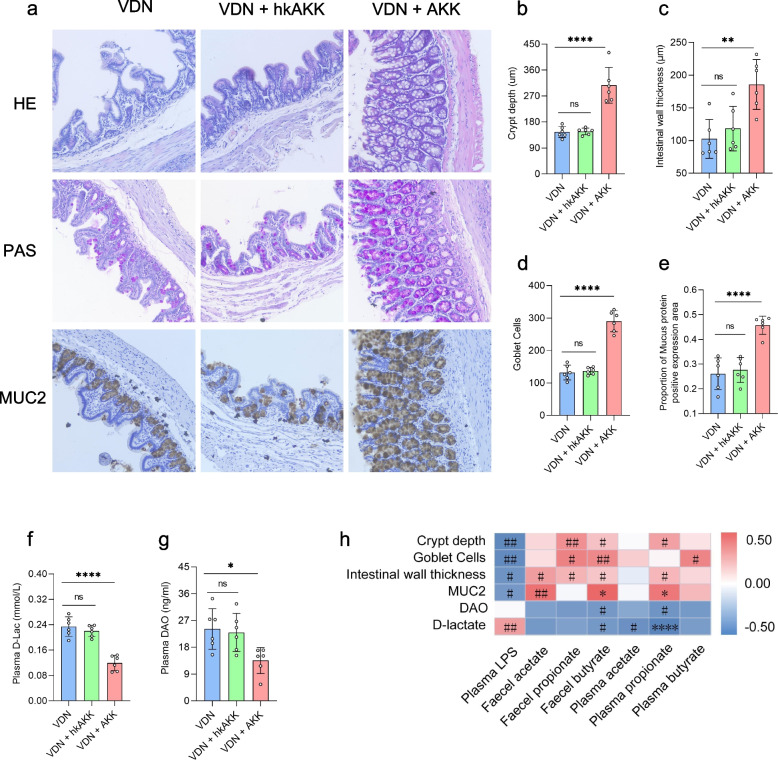
Alleviation of intestinal mucosal barrier impairment in VDN-treated rats by *Akkermansia.* Typical haematoxylin-eosin (HE) staining, periodic acid-Schiff (PAS) staining and MUC2 immunohistochemical staining for intestinal tissues (original magnification ×200). **b–e** Parameters of crypt depth, intestinal wall thickness, goblet cell count and MUC2 expression levels, respectively. **f**, **g** Plasma D-lactate and diamine oxidase contents, respectively. **h** Spearman’s correlation analysis of the relationship of SCFAs and LPS with intestinal barrier-related parameters (e.g. crypt depth, intestinal wall thickness, MUC2 level and goblet cell count). Red and blue denote positive and negative correlations, respectively. Data are presented as the mean±standard deviation (SD). Statistical significance was determined using one-way ANOVA (Tukey post hoc test). NS for *P* > 0.05, #*P* < 0.25, ##*P* < 0.1, *P< 0.05, **P< 0.01, ***P< 0.001, ****P< 0.0001

As before, colon histology was analysed to investigate the effect of *Akkermansia* on the integrity of the intestinal barrier in VDN-treated rats. In comparison with that of the VDN group, there was an improvement in the intestinal mucosal integrity of rats in the VDN + AKK group, which manifested as increased crypt depth (Fig. 10a, b), intestinal wall thickening (Fig. 10a, c), an elevated goblet cell count (Fig. 10a, d) and increased MUC2 expression (Fig. 10a, e). Moreover, D-lactate and diamine oxidase levels were significantly decreased (Fig. 10f, g). In the VDN + hk-AKK group, there was no evident change in intestinal barrier parameters or permeability parameters of VDN-treated rats (Fig. 10a–g). As demonstrated by correlation analysis, intestinal barrier-associated parameters (crypt depth, intestinal wall thickness, goblet cell count and MUC2 level) were negatively correlated with LPS but positively correlated with SCFAs. D-Lactate and diamine oxidase had negative correlations with SCFAs (Fig. 10 h).

In summary, these results indicate that *Akkermansia* has a protective effect on the intestinal mucosal barrier.

In summary, by modulating the intestinal microbiota, *Akkermansia* can enhance intestinal microbiota diversity, promote SCFA production, mitigate intestinal barrier impairment, reduce inflammation and eventually alleviate VDN-induced vascular calcification.

## Discussion

The intestinal microbiota or its components and metabolites can directly or indirectly participate in the regulation of local and systemic pathophysiological processes of the host, hence greatly impacting the cardiovascular health of the host. Products of dietary fibre fermentation by the intestinal microbiota [9, 10], SCFAs can ameliorate risks for cardiovascular diseases [11] and can inhibit the progression of these diseases [17, 21]. In our experiment, we constructed a VDN-induced vascular calcification model in rats to gain insights into the interactions of propionate, the intestinal microbiota and vascular calcification. Here, it was found that propionate could reduce vascular calcification in VDN-treated rats, which was mediated by intestinal microbiota remodelling, to promote SCFA production, improve intestinal barrier function and reduce inflammation. Interestingly, supplementation with *Akkermansia* can also alleviate vascular calcification, producing an action consistent with that of propionate.

This observational cohort study indicates that SCFAs, especially propionate, suppress vascular calcification. In addition, we found that SCFAs in plasma and faeces were inversely associated with TC, LDL, FBG and BMI. Recent studies have found that mice exposed to propionate for a long time developed insulin resistance, and similar results have also been observed in placebo-controlled trials in humans [31]. In addition, Sanna et al. found that an increased risk of type 2 diabetes was closely associated with elevated propionic acid levels in faeces [32]. In contrast, some studies have shown that propionic acid improves islet β cell function, regulates insulin secretion [33], reduces digestibility by inhibiting amylase activity [34] and delays gastric emptying and glucose absorption from the diet [35, 36], all of which maintain blood glucose balance. Furthermore, a meta-analysis showed that acute or chronic propionate interventions had no significant effect on fasting glycaemia or fasting insulin, postprandial glucose or postprandial insulin levels [37]. In addition, propionate also has a crucial effect on body weight. Tirosh et al. found that humans and mice gained weight when they were exposed to propionate for a long time [31]. However, many studies have also shown that orally delivered SP increases resting energy expenditure and lipid oxidation [38, 39].

Regarding the different effects of propionate on blood glucose content and body weight, we think that propionate acts on different metabolic processes. Propionate in the intestine functions more locally, but in the circulatory system, propionate works on complex systemic metabolism. Obesity is the strongest risk factor for diabetes [40]. Exposure to propionate and deletion of the FABP4 gene prevent weight gain and the development of anti-insulin antibodies [31]. Startlingly, studies have revealed that increasing propionate content in stool predicts an increased risk of type 2 diabetes, which is related to genetics [32]. All these results suggest that genetics increase the susceptibility to diabetes and obesity [41, 42]. Many studies suggest that obesity and diabetes correlate with the intestinal microbiota [43, 44]. Diets change the intestinal microbiota, which responds differently to diverse dietary structures [43, 45]. Furthermore, various gut microbial enterotype varieties have various effects on obesity and diabetes [44]. Future studies will focus deeply and broadly on the intestinal microbiota and disease analysis.

Several previous investigations demonstrated abnormal microbial community structures in rats with vascular calcification and chronic kidney disease [27, 46]. Here, in our study, we detected the gut microbiota composition of VDN-treated rats by 16S RNA sequencing analysis. We found that the α-diversity of the gut microbiota decreased, and the β-diversity results also indicated that the microbiota composition of VDN-treated rats was significantly changed. The ratio of *Firmicutes* to *Bacteroidetes* is often regarded as a measure of gut microbial health. In the VDN-induced vascular calcification rat model, the ratio of *Firmicutes* to *Bacteroidetes* was significantly shifted, indicating that the intestinal microbiota was in a disordered state. Additionally, at various taxon levels, pathogenic gut microbiota constituents, such as *Proteobacteria*, *Escherichia* and *Shigella*, were significantly enriched. *Proteobacteria* are a major source of gut-derived endotoxins (LPS), which can disrupt and penetrate the intestinal barrier and enter the systemic circulation [47, 48]. Vascular calcification is closely associated with chronic nonspecific inflammation. LPS activates toll-like receptor 4 (TLR4) in macrophages and promotes the production of IL-6, IL-1β and TNF-α [49]. Among them, TNF-α upregulates MSX2 in VSMCs through the NF-KB pathway [50]; IL-6 regulates BMP-2-Wnt/β-catenin signalling [51]. Additionally, the activated inflammasome converts inactive pre-IL-1β into IL-1β, which is active and increases vascular calcification [27].

Diet is a key factor that influences the compositions and diversity of the intestinal microbiota [52]. It is well known that SCFAs originate from the metabolism of the gut microbiota and can regulate the homeostasis of the gut microbiota. In our research, oral and rectal propionate administration indeed altered the α-diversity and β-diversity of the intestinal microbiota in rats with VDN-induced vascular calcification, increased the abundance of SCFA-producing microbiota constituents and lowered the abundance of LPS-producing bacteria. Increased SCFA levels in the circulatory system and intestine participate in the pathophysiological host process by binding SCFA receptors [53]. SCFA receptors include FFA2, FFA3 and GPR109A [53]. FFA2 and FFA3 are expressed in VSMCs, endothelial cells and immune cells [54, 55]. LPS upregulates the expression of FFA2, FFA3 and GPR109A by stimulating monocytes and macrophages [56]. Acetate, propionate, and butyrate activate related receptors and reduce the levels of inflammatory mediators such as TNF-α, IL-4, IL-5, IL-17a and IL-6 [57–60]. Additionally, butyrate and propionate inhibit NF-κB activity and HDACs in monocytes and macrophages and present anti-inflammatory effects [61]. SCFAs also inhibit the migration and recruitment of immune cells [62]. Hendrik et al. found that SP improved cardiac immune cell infiltration, decreased inflammation and then relieved atherosclerosis, myocardial fibrosis and hypertrophy [21]. In our study, we also found that propionate reduced macrophage infiltration in vascular tissue. In addition, SCFAs provide beneficial bacteria with an appropriate living environment by reducing pH values in the intestinal tract and competitively inhibit malignant bacteria, thus sustaining the intestinal microecological balance [63, 64]. Furthermore, it has been reported in previous research that rats at an early stage of chronic kidney disease supplemented with dietary fibre, a precursor substance of SCFAs, can suppress the progression of vascular calcification. The authors suggested that the protective effects on blood vessels are attributed to SCFA reduction of uraemia toxin levels derived from intestinal bacteria [46]. In addition, the gut microbiota provides an important structural basis for the gut microbial barrier, and a healthy gut microbiota promotes the maintenance of gut barrier function. In the VDN + SP group, propionate regulated the homeostasis of the intestinal microbiota, improved intestinal mucosal permeability, decreased LPS infiltration into the blood circulation and finally lessened the interaction between LPS and TLR4, decreasing the secretion of inflammatory factors downstream of the signalling pathway.

Subsequently, to further examine the above hypothesis, which states that the propionate-modulated intestinal microbiota was the target of propionate in reducing vascular calcification, we carried out a faecal microbiota transplantation (FMT) experiment. It is interesting to note that the propionate-modulated intestinal microbiota successfully attenuated vascular calcification and inflammatory responses, with similar results for oral and rectal propionate administration. Further analysis of the gut microbiota showed that the α-diversity was significantly increased in the VDN + SP→VDN group; in addition, the β-diversity analysis showed that the microbiota structure of the VDN + SP→VDN group was significantly different from that of the VDN group. Comparison at the taxon level showed that the LPS-producing microbiota was significantly depleted in the VDN+SP→VDN group, and the SCFA-producing microbiota was significantly enriched, suggesting that the VDN+SP→VDN group had restoration of the healthy microbiota in VDN-induced vascular calcified rats. Notably, in our study, we detected increased *Bacteroidetes* abundance in the VDN-treated rats in the first experiment. However, *Bacteroidetes* were significantly depleted after transplantation with propionate-modulated microbiota treatment. *Bacteroidetes* play a dual role as beneficial and opportunistic pathogenic bacteria. In some studies, *Bacteroidetes* is believed to be associated with intestinal inflammation [65]; however, in other literature, *Bacteroidetes* is related to the amelioration of endotoxaemia and suppression of inflammation [66]. In our study, whether the increase in *Bacteroidetes* abundance was a driving factor of vascular calcification aggravation or a result of the body’s remedial compensatory mechanism still needs further experimental verification. Collectively, our data support the conclusion that the propionate-modulated intestinal microbiota can ameliorate VDN-induced vascular calcification in rats by re-establishing a normal microbial community.

In the present study, *Akkermansia* was enriched in the VDN + SP, VDN + rectal-SP and VDN + SP→VDN groups, and it also made the greatest contribution. As a next-generation ‘probiotic’ candidate [30], *Akkermansia* has the potential to ameliorate lipid and glucose metabolic disorders, reduce inflammation and improve intestinal barrier function [67]. Therefore, *Akkermansia* regulation is deemed a target to treat the following diseases: Hutchinson-Gilford syndrome [68], amyotrophic lateral sclerosis [69], coronary atherosclerosis [70], obesity [71], insulin resistance [71] and alcoholic liver disease [72]. In our study, *Akkermansia* transplantation was performed to confirm whether *Akkermansia* was able to ameliorate vascular calcification. Interestingly, *Akkermansia* supplementation was suggested to relieve vascular calcification. Analysis of gut microbiota diversity showed that orally delivered *Akkermansia* changed the composition and structure of the microbiota. *Akkermansia* is a bacterium that degrades mucins, producing acetate, propionate and small nitrogen-containing molecules [73]. Non-mucin-degrading bacteria utilise carbon and nitrogen sources provided by *Akkermansia*, promoting their own growth and colonisation. It has been reported that cocultured *Akkermansia* and non-mucin-degrading butyrate-producing bacteria, such as *Faecalibacterium prausnitzii*, *Anaerostipes caccae*, *Roseburia* and *Eubacterium hallii*, can mutually promote growth and butyrate production [74, 75]. In the present study, it was also found that orally delivered *Akkermansia* significantly increased the abundance of SCFA-producing bacteria, promoted plasma and faecal SCFA levels, reduced the abundance of LPS-producing bacteria and reduced the source of plasma LPS. Research has shown that *Akkermansia* can prevent high-fat diet-induced dysbiosis by depleting obesity-related pathogenic bacteria and enriching health-related gut microbiota constituents [76]. Furthermore, oral administration of live and pasteurised *Akkermansia* and its extracellular vesicles could normalise the composition of the gut microbiota, ameliorate intestinal permeability, regulate inflammatory responses, and subsequently prevent liver injury in high-fat diet (HFD)- and carbon tetrachloride (CCl4)-treated mice [77]. However, some studies have shown that *Akkermansia* does not improve the composition of the gut microbiota [78, 79].

Mucus covers the outer intestinal epithelial cell layer and plays a physical protective role in blocking the penetration of the microbiota and harmful compounds [80]. In addition to mucin degradation, *Akkermansia* has been found to stimulate mucin production. In animal models, *Akkermansia* restores the thickness of the intestinal mucus layer [78], increases the number of goblet cells [72] and enhances the expression of tight junction proteins (Occludin and ZO-1) in the intestinal mucosa [70]. Our study showed that supplementation with *Akkermansia* also significantly improved the intestinal mucosal barrier and permeability and reduced LPS infiltration into the systemic circulation. In previous studies, inactivated *Akkermansia* was found to improve intestinal barrier functions owing to an interaction between toll-like receptor 2 (TLR2) and Amuc_1100, a specific protein in the outer membrane of *Akkermansia* [71]. Nevertheless, no similar protective effects were observed from heat-killed *Akkermansia* in our study, which conforms to previous research findings [78]. A possible reason for the results in our study is that *Akkermansia* promotes the growth of beneficial microbiota producing SCFAs, improves the intestinal barrier, prevents LPS from penetrating the blood circulation to a certain extent and reduces inflammation. Inactivated *Akkermansia* fails to exert its bioactivity functions. Furthermore, how *Akkermansia* contributes to mucus thickness remains unclear. One reason may be that *Akkermansia* stimulates the turnover rate of mucus renewal by making SCFAs from degraded mucins, a preferred energy source for the host epithelium that synthesises and secretes mucins [81].

There is evidence for the anti-inflammatory properties of *Akkermansia* in different mouse models, including atherosclerosis [70], liver injury [82] and metabolic disease [83]. Akkermansi*a* is involved in the regulation of inflammation from multiple aspects, including vesicles secreted by *Akkermansia*, which can reduce the expression of TLR4, reduce the level of plasma LPS-binding protein (LBP) and inactivate LPS/LBP downstream signalling [83]; moreover, SCFAs can inhibit histone deacetylases, ultimately regulating the NF-κB pathway and reducing the secretion of proinflammatory factors. Furthermore, *Akkermansia* modulates immune homeostasis and suppresses proinflammatory immune cell activity. In the present study, oral administration of *Akkermansia* also reduced plasma LPS and proinflammatory factor levels and inhibited vascular macrophage infiltration and proinflammatory factor expression. In conclusion, in a rat model of VDN-induced vascular calcification, oral administration of *Akkermansia* could support intestinal microbial homeostasis, promote SCFA production, protect the intestinal mucosal barrier, improve mucosal barrier permeability, modulate the inflammatory response and subsequently prevent vascular calcification.

In this study, we identified some SCFA-producing bacteria, such as *Akkermansia* and *Bifidobacterium*, but we only selected *Akkermansia* as a research object. However, we are convinced that other SCFA-producing bacteria also have similar effects and even synergistic effects among bacterial genera [84, 85]. Similarly, based on the AUC results, we selected propionate as the research object. In fact, accumulating evidence indicates that acetate and butyrate also have anti-inflammatory and immune functions and play an important role in cardiovascular disease [11]. Therefore, we believe that acetate and butyrate can relieve vascular calcification, which is consistent with the observed activities of propionate. In addition, their potential and mechanism(s) by which the SCFA propionate and *Akkermansia* maintain intestinal microbiota homeostasis and improve vascular calcification merit continued investigation.

## Conclusions

**Fig. 11:**
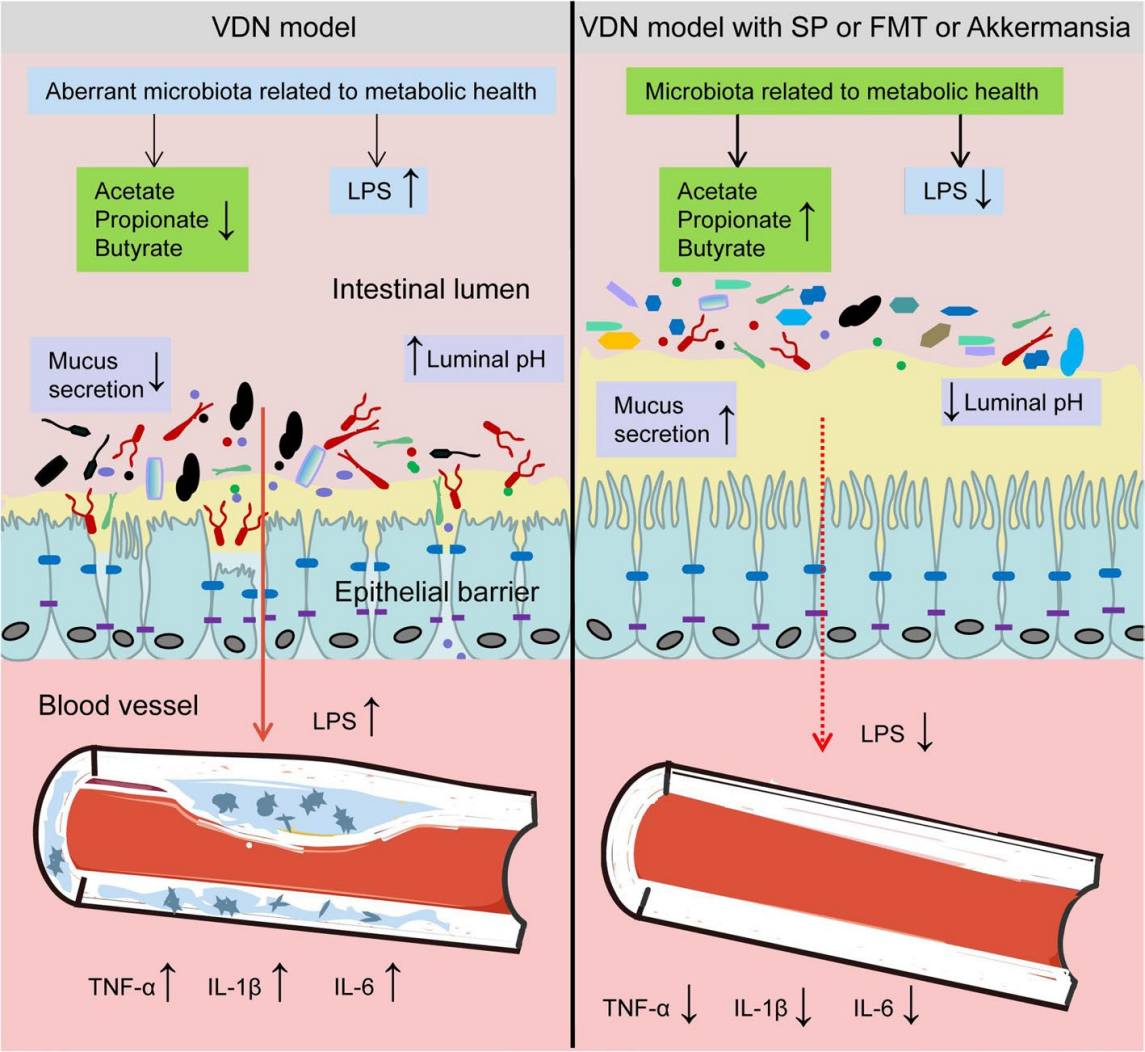
A schematic diagram of the mechanism of action. Rats with VDN-induced vascular calcification had intestinal microbiota homeostasis imbalance, decreased SCFA production, increased LPS content, mucosal barrier integrity impairment and gut ‘leakage’, which can trigger systemic inflammatory responses, finally aggravating vascular calcification. Supplementation with the SCFA propionate, faecal microbiota transplantation or *Akkermansia* transplantation can maintain the balance of the intestinal microbiota, promote the production of SCFAs and reduce LPS levels. In this way, propionate could protect the integrity of the mucosal barrier, prevent gut ‘leakage’ and inhibit inflammatory responses, resulting in the alleviation of vascular calcification

In summary, our research shows that the SCFA propionate can mitigate VDN-induced vascular calcification. Moreover, its effects on the intestinal microbiota composition and metabolites imply that diet plays a crucial part in shaping the intestinal microbiota. Propionate can promote the enrichment of microbiota constituents that contribute to SCFA production and maintenance of a healthy intestinal barrier, reduce inflammation and eventually relieve vascular calcification. Figure 11 summarises the mechanism of action of propionate. Our research findings provide new insights into propionate-mediated vascular calcification relief and provide a theoretical basis for targeting the improvement of the intestinal microbiota.

## Materials and methods

### Clinical observational cohort study and analysis

A total of 258 patients with chest pain hospitalised in the Cardiovascular Medicine Department of Shenzhen People’s Hospital (ClinicalTrials.gov ID: NCT04864457) from July 2020 to June 2021 were preliminarily and consecutively enrolled in this study. Vascular calcification scores of aortas were calculated for all enrolled patients, in addition to the collection of demographic, medical history (e.g. hypertension, diabetes and coronary heart diseases) and biochemical data. In addition, faeces and blood samples were collected. (1) Inclusion criteria included patients with completed chest computed tomography (CT) examinations. (2) Exclusion criteria included patients administered antibiotics, probiotics, prebiotics, synbiotics or purgatives and laxatives within 4 weeks; patients with intestinal diseases, including diarrhoea, constipation and haemorrhage; patients who were unwilling to participate in this research; patients with missing data; and patients with tumours. All included research subjects provided written informed consent, and the research scheme was approved by the Ethics Committee of Shenzhen People’s Hospital.

Depending on Agatston scores [86], the sum of calcification scores of the ascending aorta, aortic arch and descending thoracic aorta (from the root of ascending aorta to the diaphragm) was calculated to represent the calcification score of the aorta [87]. Computed tomography (CT) values no less than 130 Hounsfield units (HU) in the lesion site and calcified area ≥1 mm^2^ were used as standards for judging calcification. When the peak CT value had a range of 130–199 HU, the coefficient of calcification was assigned as 1. In the case of a CT peak at 200–299 HU, the coefficient was 2. For a peak value of CT ranging from 300 to 399 HU, the coefficient was assigned as 3. If the CT value had a peak no less than 400 HU, the coefficient was 4. The calcification score was obtained by multiplying the calcified area with the corresponding fixed coefficient. Moreover, each CT image was independently analysed. The sum of the calcification scores of all CT images was the aortic calcification score of the corresponding patient.

### Questionnaire survey

The China Prime Diet Quality Score (CPDQS) questionnaire was sent to all participants to collect their dietary information 24 h a day for 3 days, calculate the average daily intake of various foods for each participant and calculate the CPDQS score and obtain each person’s CPDQS indicator score [88]. The details of the questionnaire are provided in the Supporting Information (Additional file 20: Supplementary Table 9).

### Laboratory measurements

Fasting blood samples drawn from the antecubital vein were collected from all participants in the morning and centrifuged at 1500 rpm at 4°C for 10 min, and the supernatant was collected for the determination of FBG, LDL-C, TC, BUN and uric acid levels. We used Roche Cobas8000 c702 (ROChe, Basel, Switzerland) and Beckman Coulter AU5831 (Beckman Coulter, Brea, USA) automatic biochemical analyser to measure the above indicators according to the instructions of the kit. In addition, we assessed the estimated glomerular filtration rate (eGFR) based on the Chinese Modification of Diet in Renal Diseases equation [89].

### Animal studies

#### Oral propionate administration

All experimental animals were used in agreement with requirements for experimental animal operation and animal welfare proposed by the Animal Management Committee of Shenzhen People’s Hospital. Male Sprague–Dawley rats (7 weeks old) were purchased from the Guangdong Medical Experimental Animal Center and then raised in a specific pathogen-free animal room in the Innovation Collaboration Center of this hospital. Animals were raised with free access to drinking water and food in an animal room (temperature 25 ± 1°C; relative humidity (RH) 50–70%) with 12-h alternation of light and dark. After adaptive feeding of animals for 1 week, the first round of experiments began. Rats were randomly divided into three groups, i.e. the control group (*n* = 6), VDN group (*n* = 6) and VDN + SP group (*n* = 6). By referring to the method discussed in the relevant literature, a rat model of aortic vascular calcification was constructed [90]. In detail, intramuscular injection of vitamin D3 (300,000 IU/kg, Sigma Aldrich) was implemented for rats in the VDN and VDN + SP groups, associated with simultaneous intragastric administration of nicotine (25 mg/kg, Merck Millipore) that was repeated once after 9 h. Rats in the control group were given an intramuscular injection of absolute ethyl alcohol of the corresponding amount and intragastric administration of peanut oil. In addition, rats from the VDN + SP group were provided free access to drinking water containing SP (200 mM, Sigma Aldrich); those in the control and VDN groups drank sodium chloride solution (200 mM, Sigma Aldrich) without limitation.

All intervention measures continued for 6 weeks. During the last 2 weeks of the experiment, faeces were collected from rats in the control (as donor rats), VDN and VDN + SP (as donor rats) groups on a daily basis for FMT and 16S rRNA gene sequencing analysis. The remaining faecal samples were stored at −80°C. Peripheral venous blood was drawn on the last day of this experiment and centrifuged for 10 min at 1500 rpm. Then, the supernatant was collected and stored at −80°C. Moreover, intestines and aortas were placed in 4% paraformaldehyde for fixation, and those left were stored at −80°C for further use.

#### Rectal propionate administration

The manner in which VDN induces vascular calcification in rats is consistent with the above. The method of rectal administration referred to the previous literature [31], with slight modifications. Briefly, a rubber infusion tube (2.0 mm in diameter and 12 cm in length) was used. After being lubricated with liquid paraffin, it was gently inserted into the anus for approximately 8 cm. SP (1 g/kg) or sodium chloride (1 g/kg) was injected into the colon of anaesthetised rats, and then the rats were lifted by the tail upside down for 3 min. After the experiment, relevant samples (e.g. faeces, serum, intestines and aortas) were collected in line with the methods described above.

#### Faecal microbiota transplantation

Prior to FMT, all rats were raised adaptively according to the above conditions for 1 week and then randomly divided into three groups: VDN (*n* = 6), control→VDN (recipient rats, *n* = 6) and VDN + SP→VDN (recipient rats, *n* = 6). Recipient rats receiving a faecal microbiota collected from the control and VDN + SP groups were named the control→VDN and VDN + SP→VDN groups, respectively. Faecal microbiota transplantation was performed in accordance with the literature [91, 92]. In detail, fresh faeces from donor rats were collected and immediately mixed with sterile phosphate-buffered saline (PBS) (1×) at a ratio of 1:10 (m:v). The mixture was vortexed vigorously for 40 s using a benchtop vortex and centrifuged at 1000×*g* (4°C) for 3 min. Afterwards, the suspension was collected and centrifuged at 6000×*g* (4°C) for 15 min, and the supernatant was discarded. The precipitate was then resuspended in sterile PBS (1×), and the bacterial suspension was delivered to recipient rats (5 mL/kg) via oral gavage within 10 min. The second day after modelling, intragastric administration was continually performed for 14 days. After the experiment, relevant samples (e.g. faeces, serum, intestines and aortas) were collected in line with the methods described above.

#### Culture and administration of Akkermansia muciniphila

*Akkermansia muciniphila* (ATCC BAA-835, Guangdong Microbial Culture Collection Center) was cultured anaerobically in brain-heart-infusion (BHI) broth (BD Bioscience, San Jose, CA) supplemented with 0.5% porcine mucin (Sigma–Aldrich) and 0.05% cysteine (Sigma–Aldrich) at 37°C for 2–3 days [70]. Before *Akkermansia* transplantation, all rats were fed adaptively according to the above conditions for 1 week. After that, the rats were randomly divided into three groups: VDN (*n* = 6), VDN+ hk-AKK (*n* = 6) and VDN + AKK (*n* = 6). The way that VDN was used to induce vascular calcification in rats was the same as the first modelling method, in which rats received vitamin D3 (300,000 IU/kg, Sigma Aldrich) by intramuscular injection and nicotine (25 mg/kg, Merck Millipore) by gavage at the same time and then nicotine by gavage again 9 h later. Starting the day after modelling, on a daily basis, rats in the VDN + AKK, VDN + hk-AKK and VDN groups received intragastric administration of 5×10^9^ cfu/200 μL active *Akkermansia*, heat-killed *Akkermansia* of an equal volume or isovolumetric sterile PBS (1×) each time for 8 consecutive days. In the VDN + hk-AKK group, *Akkermansia* were heat killed at 121°C under 225-kPa pressure for 30 min.

#### Determination of calcification

Staining of a complete artery blood vessel was performed as follows. Aortas of rats were immersed in 4% paraformaldehyde for 24 h of fixation and then subjected to 30-h staining with alkaline alizarin red (Beyotime, China) aqueous solution (alizarin red concentration 0.003%; KOH concentration 1%). Regarding tissue section staining, the tissues were placed in 2% alizarin red solution (pH = 4.2) for 5 min. The staining results were observed and photographed under an optical microscope (Nikon Eclipse 80i, Tokyo, Japan).

#### Intestinal histopathological evaluations

Tissues were fixed with 4% paraformaldehyde, dehydrated, cleared, embedded in paraffin, sliced (5 μm), dried and dewaxed in succession. Haematoxylin-eosin (HE) and glycogen periodic acid-Schiff (PAS) staining kits (Solarbio, Beijing, China) were used in the final step. An optical microscope (Nikon Eclipse 80i, Tokyo, Japan) was utilised to observe and photograph the staining results. Six microscopic fields of every section of the intestine were randomly selected.

#### MUC2 protein expression detection based on an immunohistochemical method

After fixation with 4% paraformaldehyde, colon tissues of rats were made into paraffin-embedded sections, which were dewaxed and rehydrated. Antigen repair was conducted in a 0.01 M citric acid buffer solution (pH 6.0). Endogenous peroxidase quenching was performed with 3% hydrogen peroxide. Furthermore, 5% goat serum was selected to block nonspecific antigens at room temperature for 60 min. An anti-Muc2 antibody (1:1000; Santa Cruz Biotechnology) was added in a dropwise manner and then incubated overnight at 4°C. The biotin-labelled secondary antibody (goat anti-rabbit IgG, 1:1500) was then added for subsequent incubation at 37°C for 60 min. After rinsing three times with PBS (5 min each time), a newly prepared working solution of DAB was added dropwise for visualisation. Subsequent steps included nuclear staining using haematoxylin, conventional dehydration and mounting with resin. Under a ×20 objective lens of a microscope, 6 fields of view were randomly selected and photographed. Image-Pro Plus software 6.0 was used for semiquantitative analysis of the target protein tested, and the intensity of protein expression was denoted by an area occupied by positive proteins.

#### Immunofluorescence staining

Vascular tissue was fixed with paraformaldehyde, embedded in paraffin, and sectioned at a thickness of 5 μm. Specimen sections were dewaxed and incubated with anti-CD68 (Abcam, Cambridge, MA, USA) and anti-TNF-α (Abcam, Cambridge, MA, USA) antibodies at 4°C overnight, washed with PBS and incubated with fluorescent dye-conjugated secondary antibodies (Abcam). Nuclei were counterstained with 4,6-diamidino-2-phenylindole (DAPI) for 10 min. Immunofluorescence images were captured using a fluorescence microscope (Dmi8+DFC 7000T, Leica, Wetzlar, Germany).

#### Biochemical parameters

Serum levels of diamine oxidase (DAO), interleukin-1β (IL-1β), interleukin-6 (IL-6) and tumour necrosis factor-α (TNF-α) were measured using enzyme-linked immunosorbent assay (ELISA) kits from Jianglai Biotech (Shanghai, China); D-lactate was measured using a D-lactate colorimetric assay kit (Elabscience, Wuhan, China); tissue levels of inorganic calcium and serum creatinine were measured using test kits (Nanjing Jiancheng Bioengineering Institute Co., Ltd. Nanjing, China). Serum LPS was measured using an ELISA kit (MREDA, Beijing, China). All the above biochemical parameters were determined according to the manufacturer’s instructions.

#### Microbiota DNA sequencing

Total DNA extraction was completed by means of the cetyltrimethylammonium bromide (CTAB) method [93]. DNA concentration and purity were detected via 1% agarose gels. PCR amplification was carried out for V3-V4 variable regions in 16S rRNA genes of bacteria based on the primers 341F (5′-CCTAYGGGRBGCASCAG-3′) and 806R (5′-GGACTACNNGGGTATCTAAT-3′). The sequencing data have been deposited in the Sequence Read Archive (SRA) of the National Center for Biotechnology Information (NCBI) (Bioproject: PRJNA837553), to be released upon publication. The corresponding amplification procedure was as follows: predenaturation at 98°C for 1 min→30 cycles (degeneration at 98°C for 10 s, followed by annealing at 50°C for 30 s and extension at 72°C for 30 s successively)→ extension at 72°C for 5 min (PCR amplifier: T100PCR, Bio-Rad, USA). Then, 2% agarose gel electrophoresis was performed to detect PCR products, and a Qiagen Gel Extraction Kit (Qiagen, Germany) was utilised for purification. Finally, a TruSeq® DNA PCR-Free Sample Preparation Kit (Illumina, USA) was applied to create a library, and sequencing was conducted on an Illumina NovaSeq 6000 PE250 (Novogene, Tianjin, China).

#### Bioinformatic analysis

The raw paired-end reads were first merged using FLASH v1.2.11 [94], and the raw sequences were then imported into QIMME 2 for the relevant analysis [95]. Briefly, the raw tags were demultiplexed using the plugin demux (https://github.com/qiime2/q2-demux) according to the unique barcode of each sample. Then, the plugin q2-DADA2 was applied for quality control and sequence denoising. The plugin q2-vsearch was used for chimeric sequence removal according to its de novo chimaera checking method. The sequencing volume of each sample in this research was > 50,000 reads. The detailed information for each dataset in this study can be found in Additional file 21: Supplementary Table 10. The obtained effective sequences were finally clustered into amplicon sequence variants (ASVs) based on 100% sequence similarity. The plugin classifier–sklearn (https://github.com/qiime2/q2-feature-classifier) was used for taxonomic annotation by aligning the ASVs to the SILVA v123 database. The relative abundance of taxonomic assignments at each level was calculated by collapsing the subsampled ASV table based on seven-level taxonomy strings obtained from SILVA v123. Two α indices, Shannon and observed ASVs, were calculated within each sample, among which the observed ASVs described the microbial richness alone (i.e. number of species), and the Shannon index described both species richness and evenness (i.e. the equality of distribution of the species’ frequencies). Principal coordinate analysis (PCoA) based on Bray–Curtis distances was conducted to visualise the sample differentiation or sample similarity. LEfSe was used to detect the differentially abundant features among groups.

#### Plasma sample preprocessing

1. Protein removal: Methyl alcohol was stored in a refrigerator at −20°C for 60 min in advance. Blood samples (100 μL) were collected and placed in an Eppendorf (EP) tube with a volume of 1.5 mL. Next, serum and methyl alcohol were uniformly mixed at a ratio of 1:4 (v:v), and their mixture was placed in a refrigerator at −20°C for 30 min, followed by centrifugation for 20 min at 4°C and 20,000 r/min for the collection of the supernatant.
2. Extraction: A total of 300 μL of ethyl acetate and 10 μL of formic acid (0.5%, v/v) were added to the supernatant collected above; after blending, the mixture was swirled for 30 s. Furthermore, the mixture was centrifuged at 4°C and 20,000 r/min for 3 min to collect the produced ethyl acetate layer. Then, the mixture was subjected to centrifugation 3 times under the same conditions, and the ethyl acetate layer produced was collected for subsequent derivatisation experiments [96, 97].

#### Faeces sample preprocessing

A total of 150 mg of faecal sample was taken and placed in an EP tube. Then, 1.0 mL ACN (Millipore, Billerica, MA, USA) was added to these samples, and the mixture was first subjected to swirling for 3 min and then centrifuged at 4°C and 20,000 r/min for 3 min. Then, the supernatant was collected for centrifugation under the same conditions 3 times. The supernatant finally obtained was used for later derivatisation experiments [98].

#### Derivatisation

First, 30 μL (20 μmol/mL) of TEA (Sigma Aldrich) and 15 μl (20 μmol/mL) of CMPI (Sigma Aldrich) were added and uniformly mixed through swirling before subsequent incubation at 40°C for 5 min. Another incubation was performed at 40°C for 60 min after the addition of 30 μL of (20 μmol/mL) DMED (Sigma Aldrich). After that, the mixture was blown dry with nitrogen gas, and 100 μL of ACN was added to the mixture [96–98].

#### Liquid chromatography–mass spectrometry (LC–MS) analysis

##### Liquid chromatography conditions

The chromatographic column was an ACQUITY UPLC HSS T3 column (100 Å, 1.8 μm, 2.1 mm × 100 mm) (Waters, Milford, MA, USA). Mobile phases A and B were water containing 0.1% formic acid and acetonitrile with 0.1% formic acid, respectively. The gradient elution consisted of 0~5 min, 2–10% B; 5~13 min, 10 ~ 100% B; 13~15 min, 100% B; 15~16 min, 100 ~ 2% B; and 16~20 min, 2% B. The flow rate was set at 0. 2 mL/min, the sample room temperature was set at 10°C, the column temperature was set at 25°C and an injection volume of 2 μL was applied.

##### Mass spectrometry conditions

A Q Exactive Focus system (Thermo Fisher Scientific, Santa Clara, CA**,** USA) was run in positive ion mode by means of electrospray. The following parameters were applied: ion detection, Orbitrap; ion source, HESI; heater temperature, 350°C; capillary temperature, 320°C; sheath gas flow, 40 arbitrary units; auxiliary gas flow, 10 arbitrary units; spray voltage, 3.8 kV; capillary voltage, 35 V; S-lens RF level, 50%; maximum injection time, 100 ms; scan range, m/z 70–1050; and resolution, 70,000 FWHM.

#### Methodological validation and analysis of SCFAs

1. Spiked recoveries and relative standard deviations

To verify the accuracy and repeatability of this method for SCFAs, we added 3 standard solutions at different concentrations to plasma and faecal samples. We pretreated these standard solutions with the same methods for treating the samples. Each sample with an added standard solution was tested 3 times, and the average spiked recoveries and relative standard deviation (RSD) were calculated. The details of the analysis results are provided in Additional file 22: Supplementary Table 11.

(2). SCFAs for external standard curves and control environmental acetate contamination

To convert SCFA peak area to the actual SCFA levels, external standard curves were performed (Additional file 23: Supplementary Figure 12a-f). In addition, to control environmental acetate contamination, we pretreated with an acetonitrile solution (blank control) and acetic acid standard solution (positive control) according to the methods for processing samples and then compared the peak time and relative abundance of the two groups (Additional file 23: Supplementary Figure 12g, h).

### Quantification and statistical analysis

#### Statistical analysis—human studies

First, measurement data conforming to a normal distribution were expressed as the mean ± standard deviation (mean ± SD) and analysed by using a *t* test. Measurement data with an abnormal distribution were represented by the median (M) and interquartile range (P25, P75), and the Kruskal–Wallis rank sum test was used for intergroup comparison. In addition, count data were expressed as frequencies and/or percentages, and the chi-square test was utilised for intergroup comparisons. Spearman’s rho nonparametric correlation analysis was performed to evaluate the correlations of SCFA levels in both faeces and blood with calcification scores. Univariate and multivariate regression analyses were carried out for a more profound analysis of the relationship of SCFA levels with calcification scores (Additional file 24: Supplementary Table 12 and Additional file 25: Supplementary Table 13). During multivariate regression analysis, more particularly, covariates were screened following the principle that the influence of covariates introduced in the basic model on the regression coefficient was >10% or that the *P* value of the concomitant variable to the regression coefficient was <0.1 [99, 100]. Moreover, depending on the results of multivariate regression analysis, receiver operating characteristic (ROC) curves were employed to analyse the predictive ability of SCFA levels for vascular calcification.

Multivariate regression analysis and covariate screening were performed using EmpowerStats statistical software (http://www.empowerstats.com, X&Y Solutions, Inc. Boston, MA) and R statistical software (http://www.R-project.org); GraphPad Prism version 8.0 software (GraphPad Software, San Diego, California, USA) was used to draw ROC curves and scatter diagrams. A two-tailed *P* < 0.05 indicated that the difference was statistically significant.

#### Statistical analysis—animal studies

Statistical analysis was performed with GraphPad Prism version 8.0 software (GraphPad Software, San Diego, California, USA). Data are expressed as the mean and standard deviation (mean ± SD). The normality of the distribution was determined using the Shapiro–Wilk test or Kolmogorov–Smirnov test. Significance among multiple groups was determined through ordinary one-way ANOVA or the Kruskal–Wallis test according to the sample distribution. The relationship of the microbiota with SCFA levels, intestinal barrier parameters and intestinal permeability parameters was analysed by Spearman’s correlation analysis. PCoA and PERMANOVA were performed using the OmicShare tools for data analysis (www.omicshare.com/tools). The adjusted *P* value was calculated with the Benjamini–Hochberg false discovery rate (FDR) method to correct the multiple comparisons and Spearman’s correlations [101, 102]. #*P* < 0.25, ##*P* < 0.1, *P< 0.05, **P< 0.01, ***P< 0.001, ****P< 0.0001.

## Supplementary Information

**Additional file 1: Supplementary Table 1.** Characteristics of the participants at baseline in plasma samples.

**Additional file 2: Supplementary Table 2.** Characteristics of the participants at baseline in faecal samples.

**Additional file 3: Supplementary Table 3.** Relationship between short-chain fatty acids in plasma samples and clinical indicators.

**Additional file 4: Supplementary Table 4.** Relationship between short-chain fatty acids in faecal samples and clinical indicators.

**Additional file 5: Supplementary Figure 1.** Amelioration of VND-induced rat vascular calcification and reduction of inflammation by rectal propionate administration. (a) A flow diagram of the rectal propionate administration experiment. (b) Macroscopic observation of arterial vascular calcification based on alizarin red staining. (c) Quantitative evaluation of calcium content in whole aortas. (d) Observation of vascular calcification of the ascending aorta through alizarin red staining of tissue sections (original magnification ×40). (e) Relative quantification of calcium content for the vessel sections of the ascending aorta. (f), (g) and (h) Concentrations of the proinflammatory cytokines TNF-α, IL-1β and IL-6 in plasma, respectively. AO: pure alcohol and peanut oil; VDN: vitamin D3 and nicotine; SP: sodium propionate. Data are presented as the mean ± standard deviation (SD). Statistical significance was determined using one-way ANOVA (Tukey post hoc test). NS for *P* > 0.05, ***P<0.001, ****P<0.0001.

**Additional file 6: Supplementary Figure 2.** Oral propionate administration attenuated VDN-induced microbial dysbiosis in rats. (a) Relative abundance of intestinal microbiota constituents at the genus level. (b) Plasma creatinine levels. (c) Ratio between the relative abundance of *Firmicutes* and *Bacteroidetes*. (d-r) Relative abundance of identified differentially abundant bacterial groups at different taxonomic levels. Data are presented as the mean ± standard deviation (SD). Statistical significance was determined using one-way ANOVA or the Kruskal–Wallis test (Tukey or Dunnett post hoc test). NS for *P* > 0.05, *P<0.05, **P<0.01, ***P<0.001, ****P<0.0001.

**Additional file 7: Supplementary Figure 3.** Attenuation of macrophage infiltration and expression of TNF-α in calcified vessel walls by oral propionate administration. (a) Immunofluorescence staining for macrophages and TNF-α in calcified vessel walls (original magnification ×100). (b) Quantitative analysis of the CD68-positive area. (c) Quantitative analysis of the TNF-α-positive area. Data are presented as the mean ± standard deviation (SD). Statistical significance was determined using one-way ANOVA (Tukey’s post hoc test). ***P< 0.001, ****P< 0.0001.

**Additional file 8: Supplementary Figure 4.** Attenuation of macrophage infiltration and TNF-α expression in calcified vessel walls by rectal propionate administration. (a) Immunofluorescence staining for macrophages and TNF-α in calcified vessel walls (original magnification ×100). (b) Quantitative analysis of the CD68-positive area. (c) Quantitative analysis of the TNF-α-positive area. Data are presented as the mean ± standard deviation (SD). Statistical significance was determined using one-way ANOVA (Tukey post hoc test). NS for *P* > 0.05, **P< 0.01.

**Additional file 9: Supplementary Figure 5.** Amelioration of intestinal microbiota imbalance in rats by rectal propionate administration. (a) Principal coordinate analysis (PCoA) diagram showing the β-diversity of the intestinal microbiota among the three groups. (b) and (c) α-diversity of the intestinal microbiota. (d) Relative abundance of intestinal microbiota constituents at the phylum level. (e) Analysis of the differences in the intestinal microbiota by LEfSe. (f) Spearman’s correlation analysis of the relationship of the intestinal microbiota with LPS and SCFAs. Negative and positive correlations are denoted in blue and red, respectively. (g), (h) and (i) Acetate, propionate and butyrate levels in plasma, respectively. (j), (k) and (l) Acetate, propionate and butyrate concentrations in faeces, respectively. (m) Plasma LPS levels. Data are presented as the mean ± standard deviation (SD). Statistical significance was determined using one-way ANOVA or the Kruskal–Wallis test (Tukey post hoc test). NS for *P* > 0.05, #*P* < 0.25, ##*P* < 0.1, *P< 0.05, ***P< 0.001, ****P< 0.0001.

**Additional file 10: Supplementary Table 5.** Effect of oral propionate administration on the gut microbiota composition.

**Additional file 11: Supplementary Table 6.** Effect of rectal propionate administration on the gut microbiota composition.

**Additional file 12: Supplementary Figure 6.** Rectal propionate administration attenuated VDN-induced microbial dysbiosis in rats. (a) Relative abundance of intestinal microbiota constituents at the genus level. (b-j) Relative abundance of identified differentially abundant bacterial groups at different taxonomic levels. Data are presented as the mean ± standard deviation (SD). Statistical significance was determined using one-way ANOVA or the Kruskal–Wallis test (Tukey or Dunnett post hoc test). NS for *P* > 0.05, *P<0.05, **P<0.01, ***P<0.001, ****P<0.0001.

**Additional file 13: Supplementary Figure 7.** Alleviation of intestinal mucosal barrier impairment in rats by rectal propionate administration. (a) Typical haematoxylin-eosin (HE) staining, periodic acid-Schiff (PAS) staining and MUC2 immunohistochemical staining for intestinal tissues (original magnification ×200). (b), (c), (d) and (e) Parameters of crypt depth, intestinal wall thickness, goblet cell count and MUC2 expression levels, respectively. (f) and (g) Plasma D-lactate and diamine oxidase contents, respectively. (h) Spearman’s correlation analysis of the relationship of SCFAs and LPS with intestinal barrier-related parameters (e.g. crypt depth, intestinal wall thickness, MUC2 level and goblet cell count). Red and blue denote positive and negative correlations, respectively. Data are presented as the mean±standard deviation (SD). Statistical significance was determined using one-way ANOVA (Tukey post hoc test). NS for *P* > 0.05, #*P* < 0.25, ##*P* < 0.1, *P< 0.05, **P< 0.01, ***P< 0.001, ****P< 0.0001.

**Additional file 14: Supplementary Figure 8.** Attenuation of macrophage infiltration and TNF-α expression in calcified vessel walls by the propionate-modulated intestinal microbiota. (a) Immunofluorescence staining for macrophages and TNF-α in calcified vessel walls (original magnification×100). (b) Quantitative analysis of the CD68-positive area. (c) Quantitative analysis of the TNF-α-positive area. Data are presented as the mean ± standard deviation (SD). Statistical significance was determined using one-way ANOVA (Tukey post hoc test). NS for *P* > 0.05, ***P< 0.001, ****P< 0.0001.

**Additional file 15: Supplementary Table 7.** Effect of propionate-modulated intestinal microbiota on the gut microbiota composition.

**Additional file 16: Supplementary Figure 9.** The propionate-mediated intestinal microbiota attenuated VDN-induced microbial dysbiosis in rats. (a) Relative abundance of intestinal microbiota constituents at the genus level. (b) Plasma LPS levels. (c) Ratio between the relative abundance of *Firmicutes* and *Bacteroidetes*. (d-v) Relative abundance of identified differentially abundant bacterial groups at different taxonomic levels. Data are presented as the mean ± standard deviation (SD). Statistical significance was determined using one-way ANOVA or the Kruskal–Wallis test (Tukey or Dunnett post hoc test). NS for *P* > 0.05, *P<0.05, **P<0.01, ***P<0.001.

**Additional file 17: Supplementary Figure 10.** Attenuation of VDN-induced microbial dysbiosis in rats by *Akkermansia*. (a) Venn diagram showing gut microbiota constituents coenriched by oral and rectal propionate administration and propionate-modulated intestinal microbiota transplantation. (b) Receiver operating characteristic (ROC) curves showing the ability of *Muribaculaceae* and *Akkermansia* abundance to predict vascular calcification. (c) Relative abundance of intestinal microbiota constituents at the genus level. (d) Plasma LPS levels. (e-r) Relative abundance of identified differentially abundant bacterial groups at different taxonomic levels. Data are presented as the mean ± standard deviation (SD). Statistical significance was determined using one-way ANOVA or the Kruskal–Wallis test (Tukey or Dunnett post hoc test). NS for *P* > 0.05, *P<0.05, **P<0.01, ***P<0.001, ****P<0.0001.

**Additional file 18: Supplementary Figure 11.** Attenuation of macrophage infiltration and TNF-α expression in calcified vessel walls by *Akkermansia*. (a) Immunofluorescence staining for macrophages and TNF-α in calcified vessel walls (original magnification×100). (b) Quantitative analysis of the CD68-positive area. (c) Quantitative analysis of the TNF-α-positive area. Data are presented as the mean ± standard deviation (SD). Statistical significance was determined using one-way ANOVA (Tukey post hoc test). NS for *P* > 0.05, ***P< 0.001, ****P< 0.0001.

**Additional file 19: Supplementary Table 8.** Effect of *Akkermansia* on the gut microbiota composition.

**Additional file 20: Supplementary Table 9.** Questionnaire for China Prime Diet Quality Score.

**Additional file 21: Supplementary Table 10.** The average sequencing depth and standard deviation for each dataset in this study.

**Additional file 22: Supplementary Table 11.** Spiked recoveries and relative standard deviations of short chain fatty acids in plasma and feces.

**Additional file 23: Supplementary Figure 12.** SCFAs for external standard curves and control environmental acetate contamination. (a), (b) and (c) External standard curves of short-chain fatty acids (acetic acid, propionate, butyrate) standards in plasma samples. (d), (e) and (f) External standard curves of short-chain fatty acids (acetic acid, propionate, butyrate) standards in faecal samples. (g) Peak time of acetonitrile. (h) Peak time of acetic acid.

**Additional file 24: Supplementary Table 12.** Univariate and multivariate regression analysis of risk factors for vascular calcification in the participants with plasma samples.

**Additional file 25: Supplementary Table 13.** Univariate and multivariate regression analysis of risk factors for vascular calcification in the participants with faecal samples.

## Data Availability

Data that support the findings detailed in this study are available in the supplementary information and this article. The 16S rRNA gene sequencing data in this study are available in the Sequence Read Archive (SRA) under project number PRJNA837553. Any other source data perceived as pertinent are available on reasonable request from the corresponding author or first author.

## Abbreviations

ACN: Acetonitrile
*AKK*: *Akkermansia*
ANOVA: One-way analysis of variance
AUC: Area under curve
BHI: Brain-heart-infusion
CTAB: Cetyltrimethyl ammonium bromide
CHD: Coronary heart disease
CMPI: 2-Chloro-1-methylpyridinium iodide
CT: Computed tomography
DAO: Diamine oxidase
DMED: N,N-Dimethylethylenediamine
EGFR: Estimated glomerular filtration rate
ELISA: Enzyme-linked immunosorbent assay
FMT: Faecal microbiota transplantation
HE: Haematoxylin and eosin
HK-AKK: Heat-killed *Akkermansia*
HU: Hounsfield units
IL-1β: Interleukin-1β
IL-6: Interleukin-6
IQR: Interquartile range
LC-MS: Liquid chromatography-mass spectrometry
LDL-C: Low-density lipoprotein cholesterol
LEfSe: Linear discriminant analysis coupled with effect size
LPS: Lipopolysaccharide
LDA: Linear discriminant analysis
MUC2: Mucus protein 2
OTUs: Operational taxonomic units
PAS: Periodic Acid Schiff
PBS: Phosphate buffer saline
Permanona: Permutational multivariate analysis of variance
pH: Potential of hydrogen
PCR: Polymerase chain reaction
PCA: Principal component analysis
RH: Relative humidity
ROC: Receiver operating characteristic
rRNA: Ribosomal ribonucleic acid
SCFA: Short-chain fatty acid
SCFAs: Short-chain fatty acids
SD: Standard deviation
SP: Sodium propionate
TAC: Thoracic aortic calcification
TEA: Triethylamine
TLR2: Toll like receptor 2
TNF-α: Tumour necrosis factor-α
VDN: Vitamin D3 and nicotine
