# Supplementary material for: Beneficial effect of the short-chain fatty acid propionate on vascular calcification through intestinal microbiota remodelling
Source: Microbiome. 2022 Nov 16;10:195. doi: 10.1186/s40168-022-01390-0 (PMC9667615; doi:10.1186/s40168-022-01390-0)
Supplement: Supplementary file 2 — Additional file 1: Supplementary Table 1. Characteristics of the participants at baseline in plasma samples. [file 40168_2022_1390_MOESM1_ESM.docx]

| Calcification | Plasma | | P-value |
| --- | --- | --- | --- |
|  | No (n=55) | Yes (n=34) |  |
| TAC Score | 0.0 (0.0-0.0) | 175.1 (53.7-491.5) | <0.001 |
| Age (years) | 39.0 (35.0-43.0) | 42.5 (40.0-44.0) | 0.005 |
| Male, n (%) | 48 (87.3) | 33 (97.1) | 0.117 |
| BMI (kg/m^2^) | 23.8 ± 3.9 | 25.2 ± 4.6 | 0.144 |
| CPDQS | 53.3 ± 8.0 | 52.0 ± 9.2 | 0.508 |
| Diabetes, n (%) | 7 (12.7) | 8 (23.5) | 0.186 |
| Hypertension, n (%) | 15 (27.3) | 9 (26.5) | 0.934 |
| CHD, n (%) | 18 (32.7) | 16 (47.1) | 0.176 |
| Smoking, n (%) | 32 (58.2) | 23 (67.6) | 0.372 |
| Drinking, n (%) | 5 (9.1) | 8 (23.5) | 0.061 |
| TC (mmol/L) | 3.99 ± 1.67 | 4.88 ± 1.95 | 0.026 |
| LDL-C (mmol/L) | 2.58 (1.77-3.55) | 2.49 (1.99-4.09) | 0.671 |
| FBG (mmol/L) | 4.90 (4.34-5.84) | 5.11 (4.66-6.85) | 0.076 |
| eGFR (ml/min/1.73m^2^) | 104.40 (96.24-110.59) | 104.24 (91.51-109.01) | 0.692 |
| BUN (mmol/L) | 4.30 (3.50-5.19) | 4.36 (3.75-5.59) | 0.379 |
| Uric acid (umol/L) | 397.4 ± 103.7 | 420.6 ± 115.4 | 0.346 |
| Acetate (umol/L) | 29.3 ± 10.3 | 26.0 ± 9.1 | 0.028 |
| Propionate (umol/L) | 9.0 (5.1-14.9) | 2.0 (1.5-4.8) | <0.001 |
| Butyrate (umol/L) | 1.1 (0.4-5.5) | 0.3 (0.1-0.4) | <0.001 |

Supplementary Table 1. Characteristics of the participants at baseline in plasma samples.

Data are presented as mean ± standard deviation (SD), median (interquartile range [IQR]) or n (%). P value < 0.05 was considered statistically significant. BMI: Body Mass Index; BUN: Blood urea nitrogen; CHD: Coronary heart disease; CPDQS: China Prime Diet Quality Score; EGFR: Estimated glomerular filtration rate; FBG: Fasting blood glucose; IQR: Interquartile range; LDL-C: low-density lipoprotein cholesterol; SD: Standard deviation; TAC: Thoracic aortic calcification; TC: Total cholesterol.
