# Supplementary material for: Beneficial effect of the short-chain fatty acid propionate on vascular calcification through intestinal microbiota remodelling
Source: Microbiome. 2022 Nov 16;10:195. doi: 10.1186/s40168-022-01390-0 (PMC9667615; doi:10.1186/s40168-022-01390-0)
Supplement: Supplementary file 3 — Additional file 2: Supplementary Table 2. Characteristics of the participants at baseline in faecal samples. [file 40168_2022_1390_MOESM2_ESM.docx]

Supplementary Table 2. Characteristics of the participants at baseline in faecal samples.

| Calcification | Feces | | P-value |
| --- | --- | --- | --- |
|  | No (n=41) | Yes (n=26) |  |
| TAC Score | 0.0 (0.0-0.0) | 195.8 (55.7-596.0) | <0.001 |
| Age (years) | 39.0 (35.0-43.0) | 43.5 (39.2-44.0) | 0.005 |
| Male, n (%) | 36 (87.8) | 24 (92.3) | 0.557 |
| BMI (kg/m^2^) | 22.5 ± 3.0 | 26.8 ± 3.1 | <0.001 |
| CPDQS | 54.5 ± 7.8 | 51.1 ± 8.9 | 0.123 |
| Diabetes, n (%) | 4 (9.8) | 7 (26.9) | 0.065 |
| Hypertension, n (%) | 13 (31.7) | 5 (19.2) | 0.262 |
| CHD, n (%) | 15 (36.6) | 14 (53.8) | 0.165 |
| Smoking, n (%) | 25 (61.0) | 17 (65.4) | 0.716 |
| Drinking, n (%) | 5 (12.2) | 6 (23.1) | 0.241 |
| TC (mmol/L) | 3.91 ± 1.76 | 5.20 ± 2.13 | 0.009 |
| LDL-C (mmol/L) | 2.48 ± 0.96 | 3.71 ± 1.78 | <0.001 |
| FBG (mmol/L) | 5.26 ± 1.43 | 6.87 ± 2.59 | 0.002 |
| eGFR (ml/min/1.73m^2^) | 105.39 (97.93-111.55) | 105.43 (96.50-110.25) | 0.893 |
| BUN (mmol/L) | 4.25 (3.53-5.19) | 4.20 (3.70-5.59) | 0.689 |
| Uric acid (umol/L) | 397.6 ± 97.6 | 409.0 ± 119.8 | 0.679 |
| Acetate (mg/kg) | 2328.6 ± 692.3 | 2006.3 ± 771.3 | 0.041 |
| Propionate (mg/kg) | 492.3 (319.6-1013.0) | 62.0 (38.8-237.9) | <0.001 |
| Butyrate (mg/kg) | 83.6 (40.4-179.6) | 41.9 (18.3-70.1) | 0.006 |

Data are presented as mean ± standard deviation (SD), median (interquartile range [IQR]) or n (%). P value < 0.05 was considered statistically significant. BMI: Body Mass Index; BUN: Blood urea nitrogen; CHD: Coronary heart disease; CPDQS: China Prime Diet Quality Score; EGFR: Estimated glomerular filtration rate; FBG: Fasting blood glucose; IQR: Interquartile range; LDL-C: low-density lipoprotein cholesterol; SD: Standard deviation; TAC: Thoracic aortic calcification; TC: Total cholesterol.
