# Supplementary material for: Beneficial effect of the short-chain fatty acid propionate on vascular calcification through intestinal microbiota remodelling
Source: Microbiome. 2022 Nov 16;10:195. doi: 10.1186/s40168-022-01390-0 (PMC9667615; doi:10.1186/s40168-022-01390-0)
Supplement: Supplementary file 4 — Additional file 3: Supplementary Table 3. Relationship between short-chain fatty acids in plasma samples and clinical indicators. [file 40168_2022_1390_MOESM3_ESM.docx]

Supplementary Table 3. Relationship between short-chain fatty acids in plasma samples and clinical indicators.

| Plasma samples | Acetate | P value | Propionate | P value | Butyrate | P value |
| --- | --- | --- | --- | --- | --- | --- |
| TC | -0.1219 | 0.2607 | -0.2363 | 0.0413 | -0.2248 | 0.0495 |
| LDL-C | -0.3278 | 0.0270 | -0.2372 | 0.0435 | -0.2153 | 0.0550 |
| FBG | -0.1348 | 0.2255 | -0.3176 | 0.0195 | -0.2757 | 0.0465 |
| BMI | -0.2774 | 0.0364 | -0.241 | 0.0544 | -0.2721 | 0.0339 |
| CPDQS | 0.1726 | 0.1664 | 0.2616 | 0.0476 | 0.26430 | 0.0598 |

The adjusted P-value was calculated with Benjamini-Hochberg false discovery rate (FDR) method to correct spearman’s correlations. BMI: Body Mass Index; CPDQS: China Prime Diet Quality Score; FBG: Fasting blood glucose; LDL-C: Low-density lipoprotein cholesterol; TC: Total cholesterol
