# Supplementary material for: Beneficial effect of the short-chain fatty acid propionate on vascular calcification through intestinal microbiota remodelling
Source: Microbiome. 2022 Nov 16;10:195. doi: 10.1186/s40168-022-01390-0 (PMC9667615; doi:10.1186/s40168-022-01390-0)
Supplement: Supplementary file 5 — Additional file 4: Supplementary Table 4. Relationship between short-chain fatty acids in faecal samples and clinical indicators. [file 40168_2022_1390_MOESM4_ESM.docx]

Supplementary Table 4. Relationship between short-chain fatty acids in faecal samples and clinical indicators.

| Faecal samples | Acetate | P value | Propionate | P value | Butyrate | P value |
| --- | --- | --- | --- | --- | --- | --- |
| TC | -0.3387 | 0.0051 | -0.3511 | 0.0036 | -0.2627 | 0.0318 |
| LDL-C | -0.2444 | 0.0463 | -0.5365 | 0 | -0.3128 | 0.01 |
| FBG | -0.2551 | 0.0372 | -0.2556 | 0.0368 | -0.1775 | 0.1508 |
| BMI | -0.3554 | 0.0039 | -0.5376 | 0 | -0.5119 | 0 |
| CPDQS | 0.3837 | 0.0025 | 0.3632 | 0.0043 | 0.4358 | 0.0005 |

The adjusted P-value was calculated with Benjamini-Hochberg false discovery rate (FDR) method to correct spearman’s correlations. BMI: Body Mass Index; CPDQS: China Prime Diet Quality Score; FBG: Fasting blood glucose; LDL-C: Low-density lipoprotein cholesterol; TC: Total cholesterol.
