# Supplementary figures and images for: Beneficial effect of the short-chain fatty acid propionate on vascular calcification through intestinal microbiota remodelling
Source: Microbiome. 2022 Nov 16;10:195. doi: 10.1186/s40168-022-01390-0 (PMC9667615; doi:10.1186/s40168-022-01390-0)

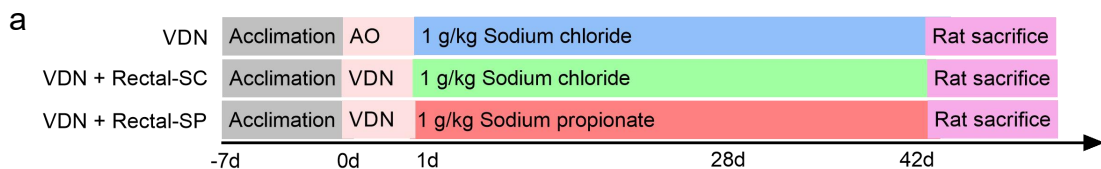

**b**      VDN      VDN + Rectal-SC      VDN + Rectal-SP

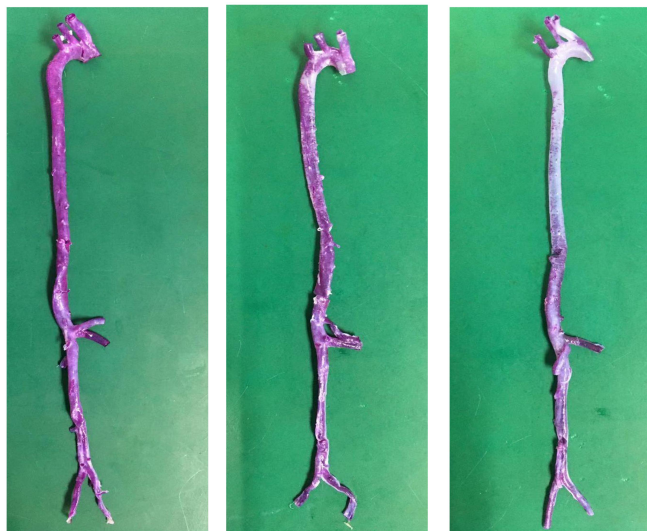

**c**

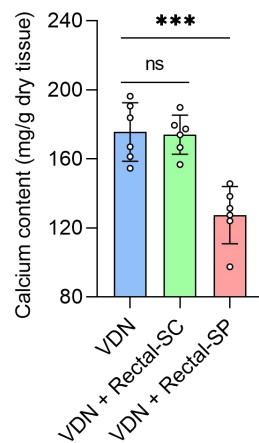

**d**      VDN      VDN + Rectal-SC      VDN + Rectal-SP

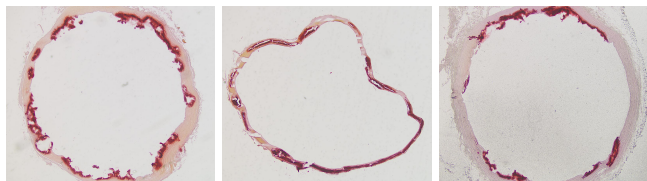

**e**

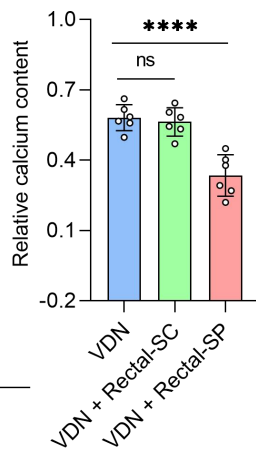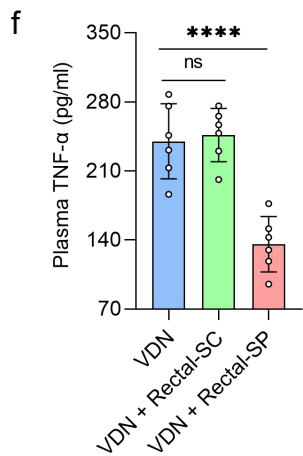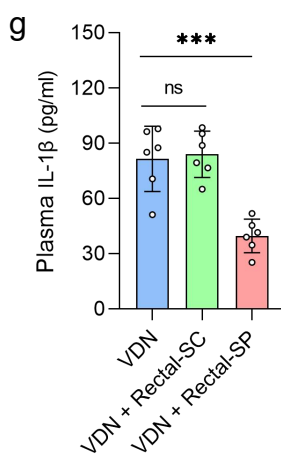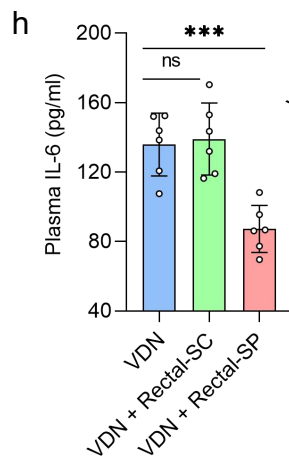

Supplement: Supplementary file 6 — Additional file 5: Supplementary Figure 1. Amelioration of VND-induced rat vascular calcification and reduction of inflammation by rectal propionate administration. (a) A flow diagram of the rectal propionate administration experiment. (b) Macroscopic observation of arterial vascular calcification based on alizarin red staining. (c) Quantitative evaluation of calcium content in whole aortas. (d) Observation of vascular calcification of the ascending aorta through alizarin red staining of tissue sections (original magnification ×40). (e) Relative quantification of calcium content for the vessel sections of the ascending aorta. (f), (g) and (h) Concentrations of the proinflammatory cytokines TNF-α, IL-1β and IL-6 in plasma, respectively. AO: pure alcohol and peanut oil; VDN: vitamin D3 and nicotine; SP: sodium propionate. Data are presented as the mean ± standard deviation (SD). Statistical significance was determined using one-way ANOVA (Tukey post hoc test). NS for P > 0.05, ***P<0.001, ****P<0.0001. [file 40168_2022_1390_MOESM5_ESM.pdf]

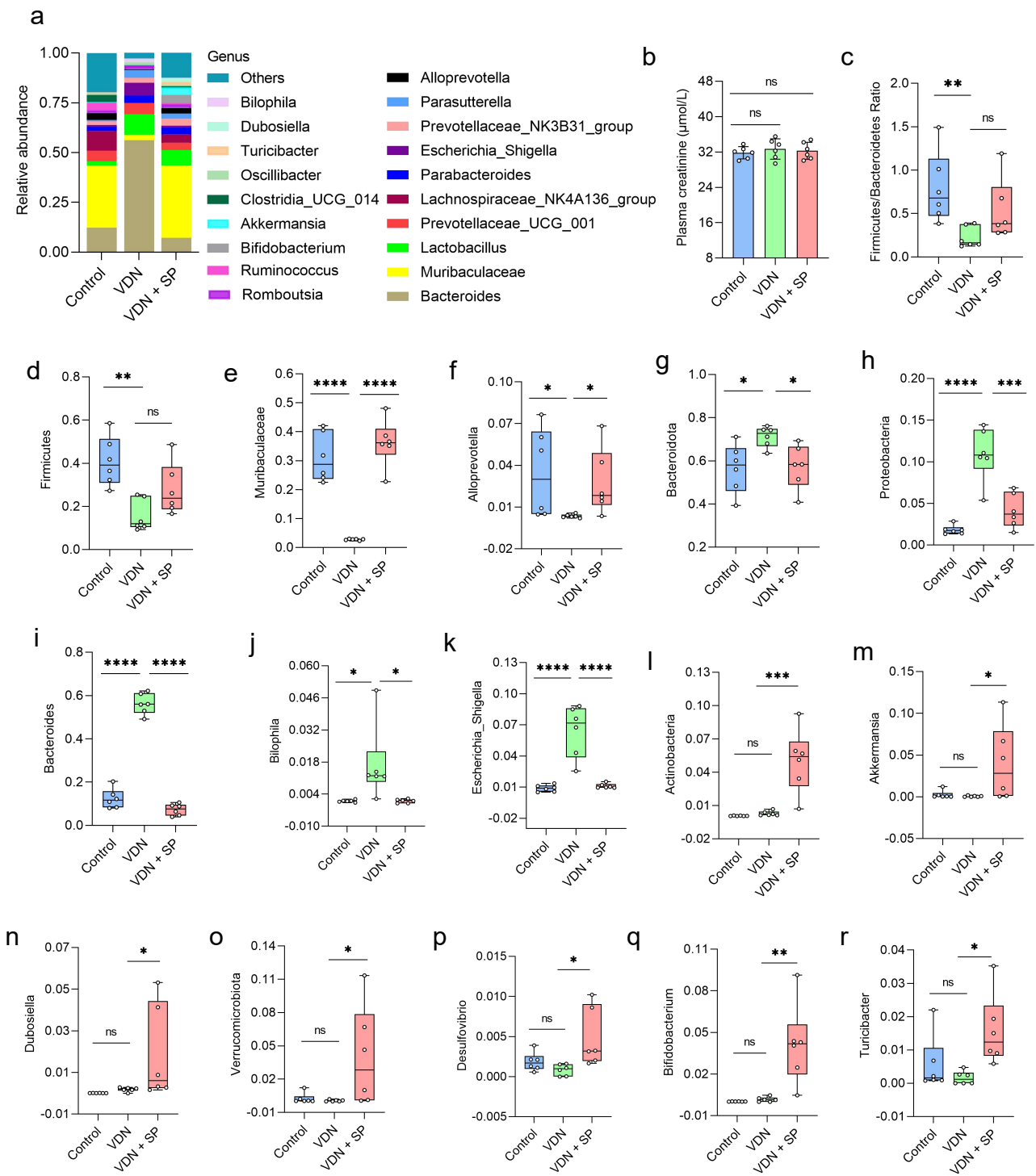

Supplement: Supplementary file 7 — Additional file 6: Supplementary Figure 2. Oral propionate administration attenuated VDN-induced microbial dysbiosis in rats. (a) Relative abundance of intestinal microbiota constituents at the genus level. (b) Plasma creatinine levels. (c) Ratio between the relative abundance of Firmicutes and Bacteroidetes. (d-r) Relative abundance of identified differentially abundant bacterial groups at different taxonomic levels. Data are presented as the mean ± standard deviation (SD). Statistical significance was determined using one-way ANOVA or the Kruskal–Wallis test (Tukey or Dunnett post hoc test). NS for P > 0.05, *P<0.05, **P<0.01, ***P<0.001, ****P<0.0001. [file 40168_2022_1390_MOESM6_ESM.pdf]

a

Control

VDN

VDN + SP

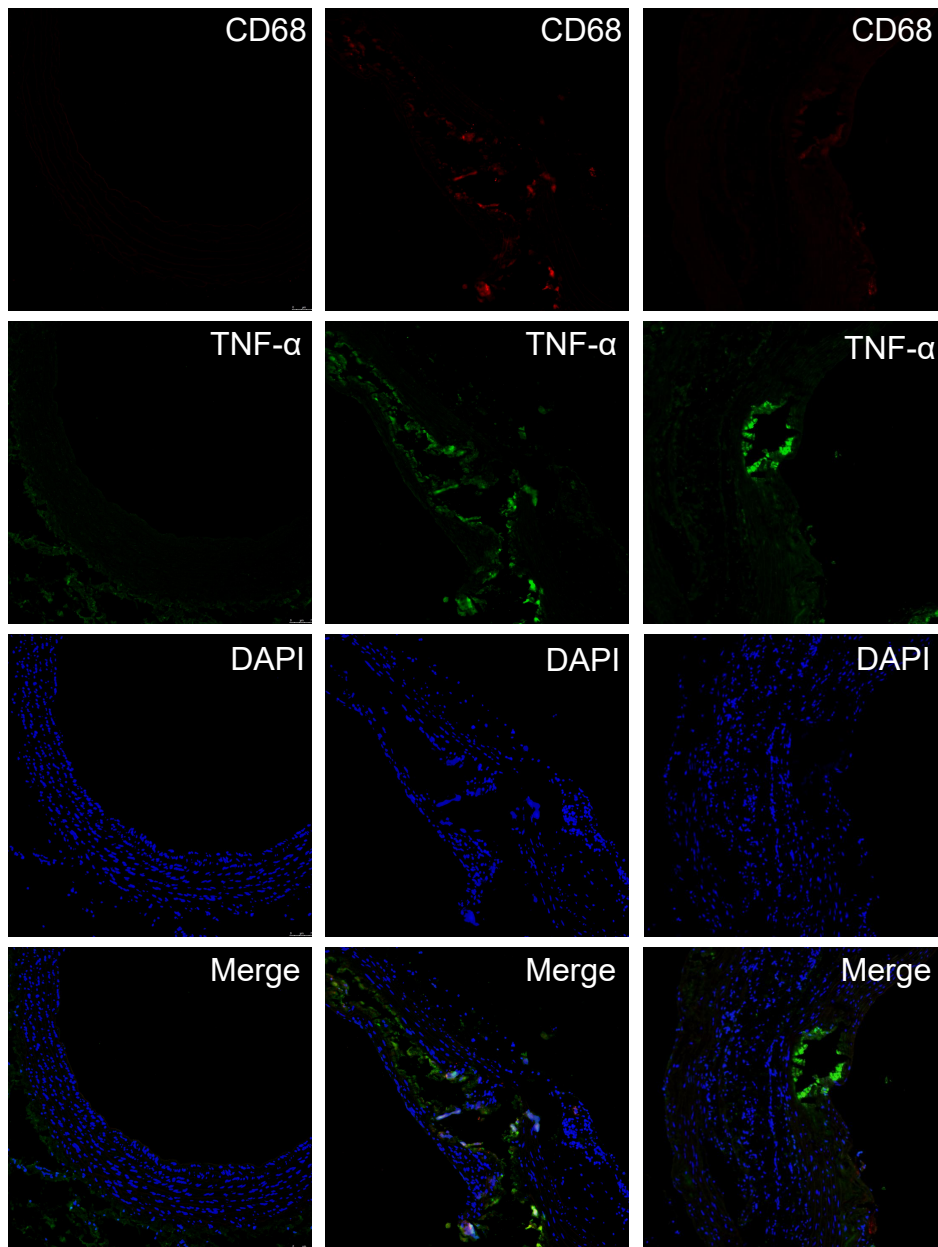

b

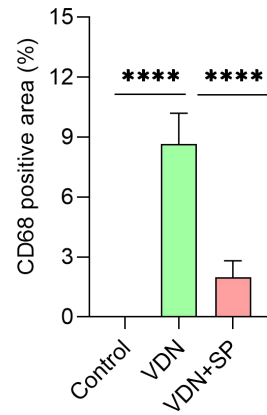

c

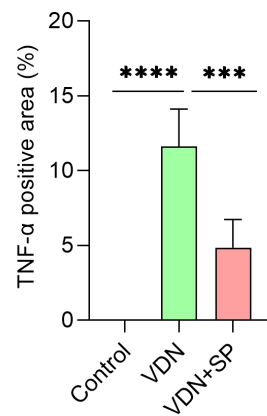

Supplement: Supplementary file 8 — Additional file 7: Supplementary Figure 3. Attenuation of macrophage infiltration and expression of TNF-α in calcified vessel walls by oral propionate administration. (a) Immunofluorescence staining for macrophages and TNF-α in calcified vessel walls (original magnification ×100). (b) Quantitative analysis of the CD68-positive area. (c) Quantitative analysis of the TNF-α-positive area. Data are presented as the mean ± standard deviation (SD). Statistical significance was determined using one-way ANOVA (Tukey’s post hoc test). ***P< 0.001, ****P< 0.0001. [file 40168_2022_1390_MOESM7_ESM.pdf]

a                      VDN                      VDN + Rectal-SC                      VDN + Rectal-SP

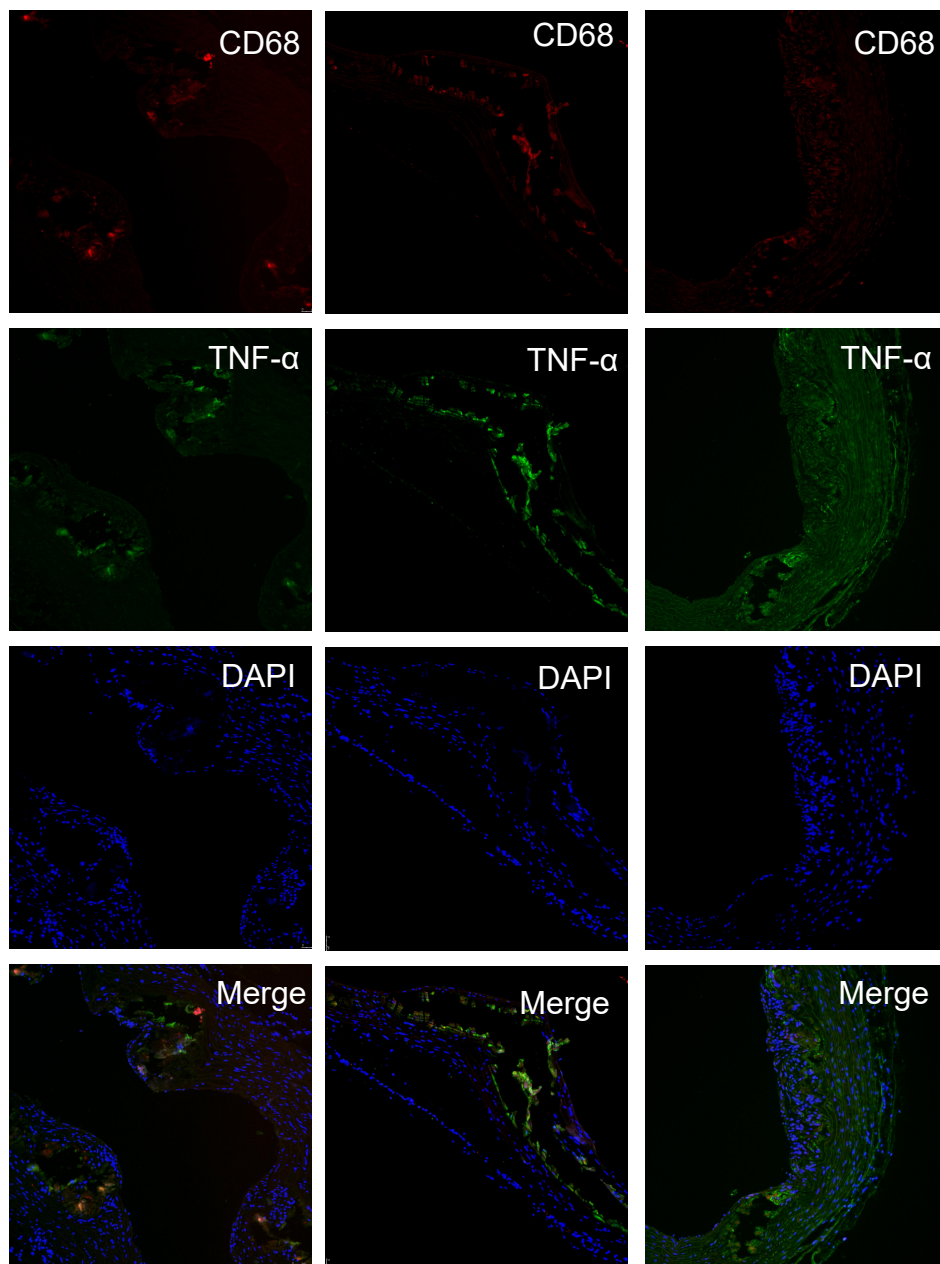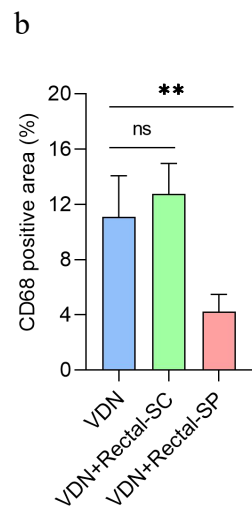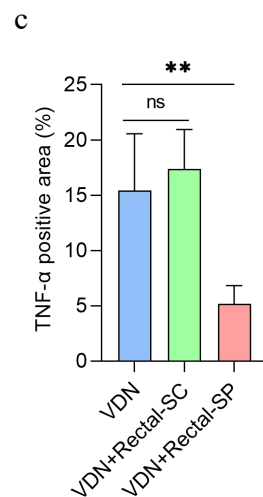

Supplement: Supplementary file 9 — Additional file 8: Supplementary Figure 4. Attenuation of macrophage infiltration and TNF-α expression in calcified vessel walls by rectal propionate administration. (a) Immunofluorescence staining for macrophages and TNF-α in calcified vessel walls (original magnification ×100). (b) Quantitative analysis of the CD68-positive area. (c) Quantitative analysis of the TNF-α-positive area. Data are presented as the mean ± standard deviation (SD). Statistical significance was determined using one-way ANOVA (Tukey post hoc test). NS for P > 0.05, **P< 0.01. [file 40168_2022_1390_MOESM8_ESM.pdf]

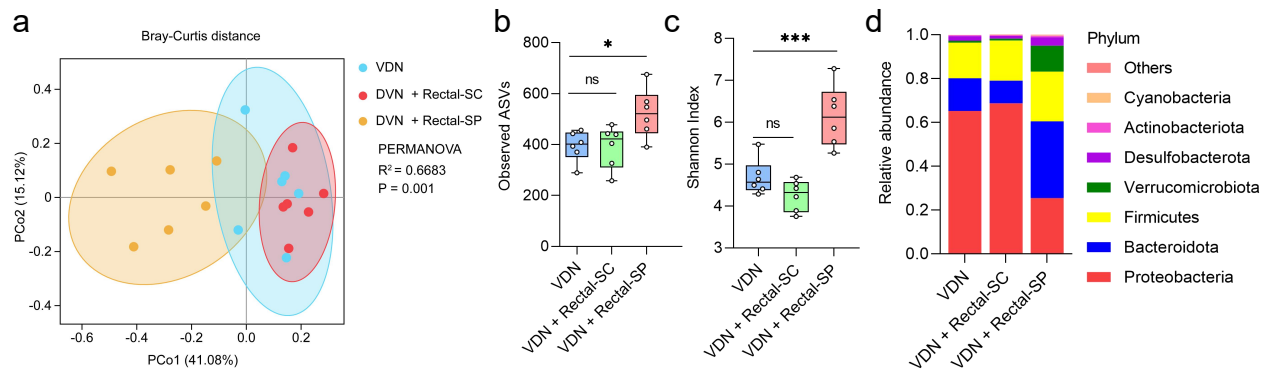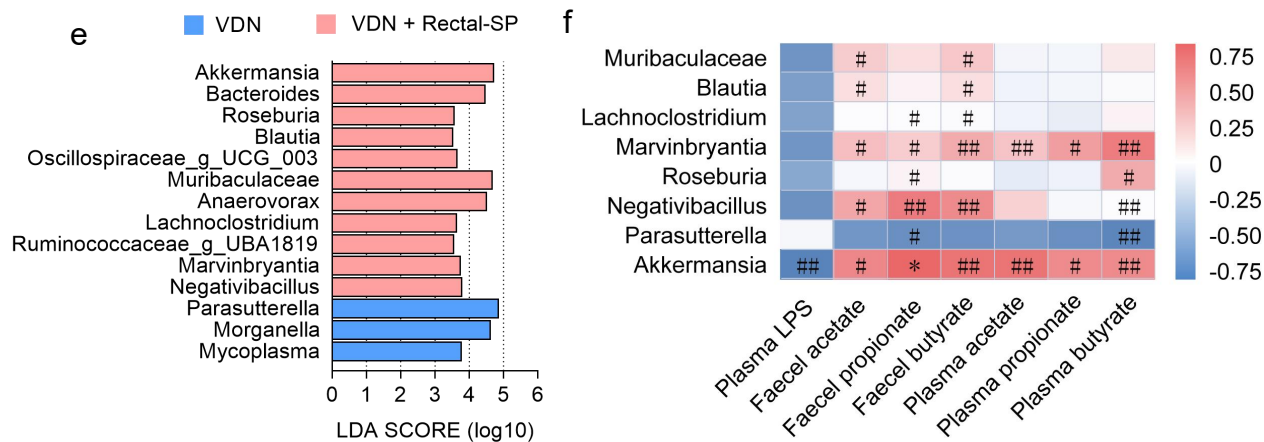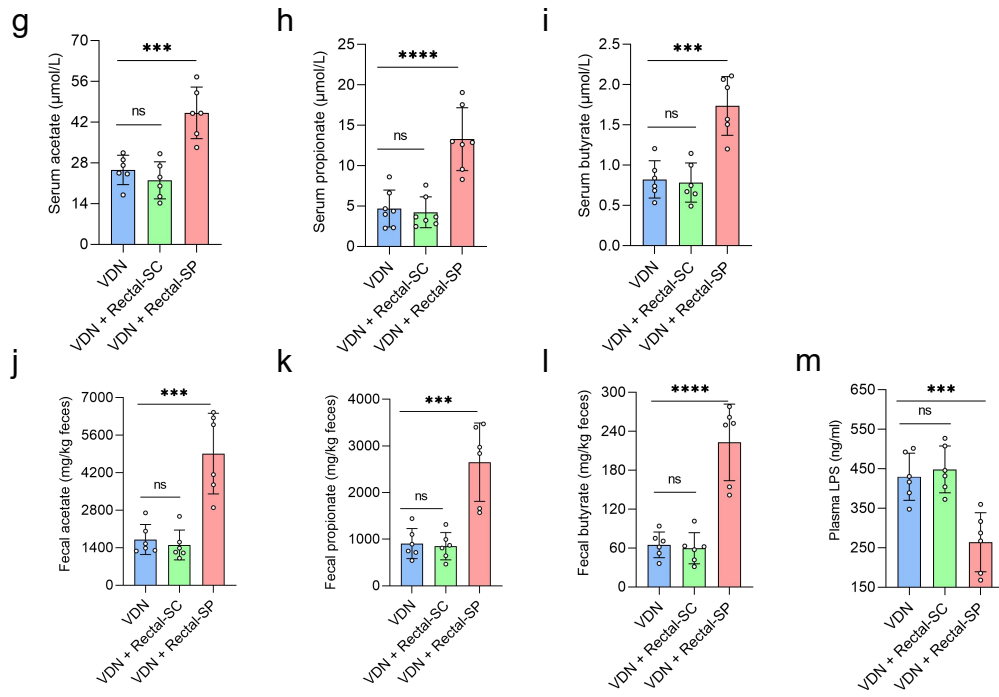

Supplement: Supplementary file 10 — Additional file 9: Supplementary Figure 5. Amelioration of intestinal microbiota imbalance in rats by rectal propionate administration. (a) Principal coordinate analysis (PCoA) diagram showing the β-diversity of the intestinal microbiota among the three groups. (b) and (c) α-diversity of the intestinal microbiota. (d) Relative abundance of intestinal microbiota constituents at the phylum level. (e) Analysis of the differences in the intestinal microbiota by LEfSe. (f) Spearman’s correlation analysis of the relationship of the intestinal microbiota with LPS and SCFAs. Negative and positive correlations are denoted in blue and red, respectively. (g), (h) and (i) Acetate, propionate and butyrate levels in plasma, respectively. (j), (k) and (l) Acetate, propionate and butyrate concentrations in faeces, respectively. (m) Plasma LPS levels. Data are presented as the mean ± standard deviation (SD). Statistical significance was determined using one-way ANOVA or the Kruskal–Wallis test (Tukey post hoc test). NS for P > 0.05, #P < 0.25, ##P < 0.1, *P< 0.05, ***P< 0.001, ****P< 0.0001. [file 40168_2022_1390_MOESM9_ESM.pdf]

**a**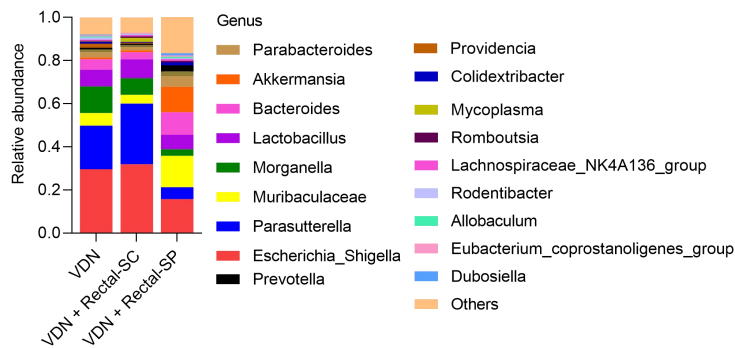**b**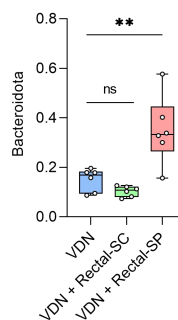**c**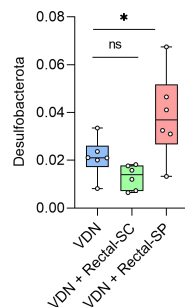**d**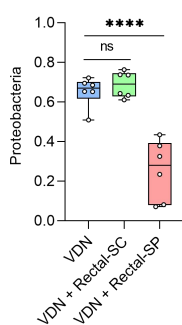**e**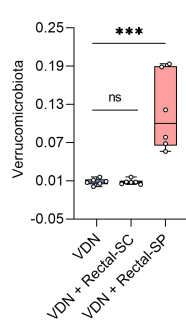**f**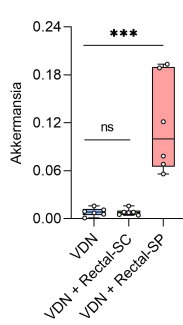**g**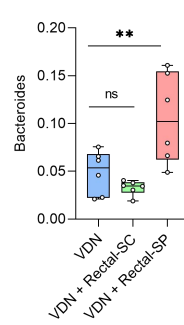**h**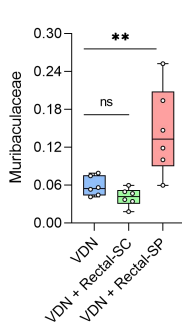**i**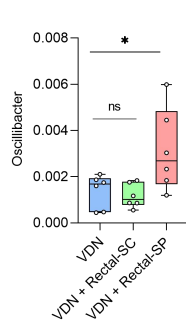**j**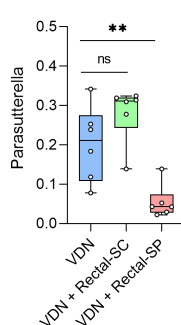

Supplement: Supplementary file 13 — Additional file 12: Supplementary Figure 6. Rectal propionate administration attenuated VDN-induced microbial dysbiosis in rats. (a) Relative abundance of intestinal microbiota constituents at the genus level. (b-j) Relative abundance of identified differentially abundant bacterial groups at different taxonomic levels. Data are presented as the mean ± standard deviation (SD). Statistical significance was determined using one-way ANOVA or the Kruskal–Wallis test (Tukey or Dunnett post hoc test). NS for P > 0.05, *P<0.05, **P<0.01, ***P<0.001, ****P<0.0001. [file 40168_2022_1390_MOESM12_ESM.pdf]

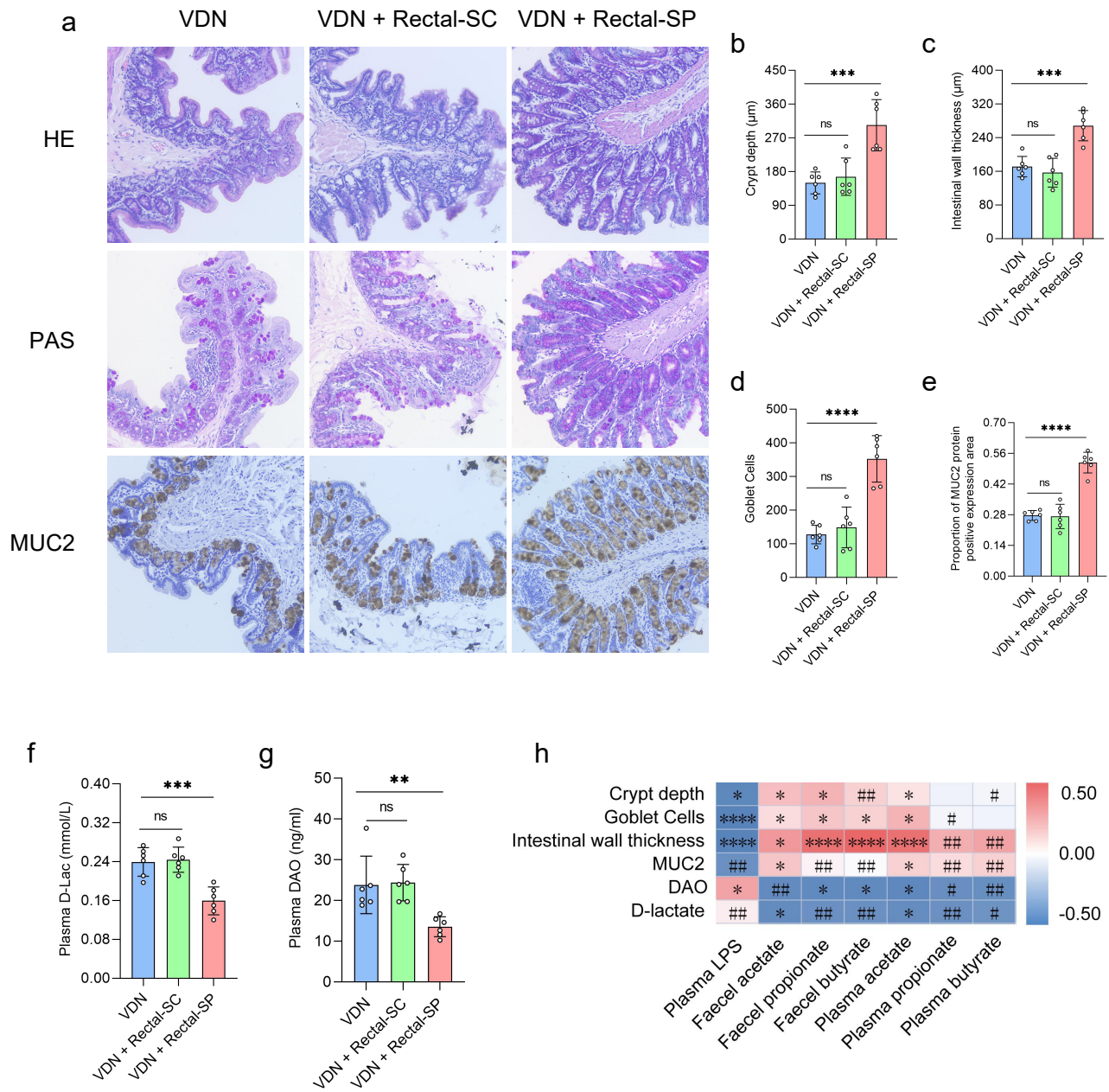

Supplement: Supplementary file 14 — Additional file 13: Supplementary Figure 7. Alleviation of intestinal mucosal barrier impairment in rats by rectal propionate administration. (a) Typical haematoxylin-eosin (HE) staining, periodic acid-Schiff (PAS) staining and MUC2 immunohistochemical staining for intestinal tissues (original magnification ×200). (b), (c), (d) and (e) Parameters of crypt depth, intestinal wall thickness, goblet cell count and MUC2 expression levels, respectively. (f) and (g) Plasma D-lactate and diamine oxidase contents, respectively. (h) Spearman’s correlation analysis of the relationship of SCFAs and LPS with intestinal barrier-related parameters (e.g. crypt depth, intestinal wall thickness, MUC2 level and goblet cell count). Red and blue denote positive and negative correlations, respectively. Data are presented as the mean±standard deviation (SD). Statistical significance was determined using one-way ANOVA (Tukey post hoc test). NS for P > 0.05, #P < 0.25, ##P < 0.1, *P< 0.05, **P< 0.01, ***P< 0.001, ****P< 0.0001. [file 40168_2022_1390_MOESM13_ESM.pdf]

a

VDN

Control→VDN

VDN + SP→VDN

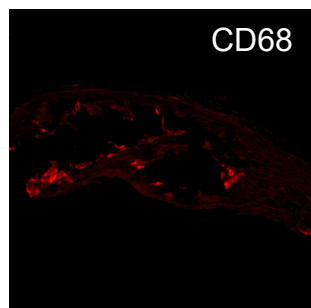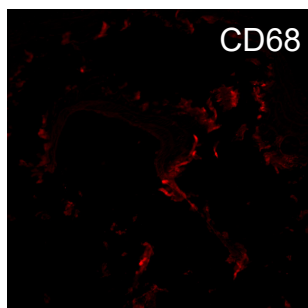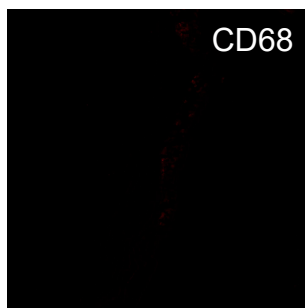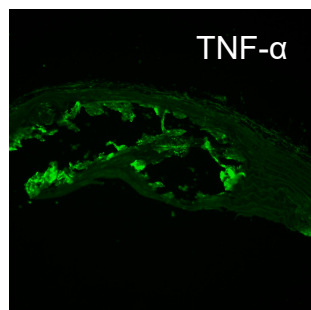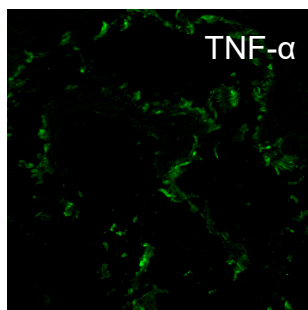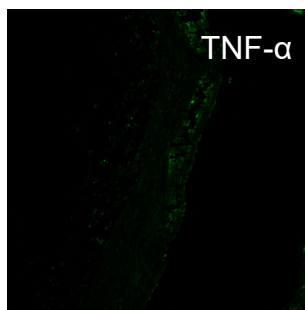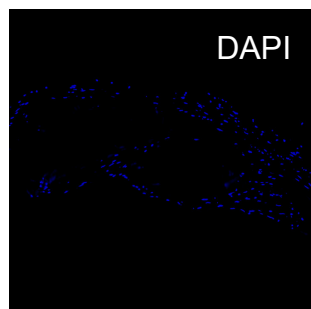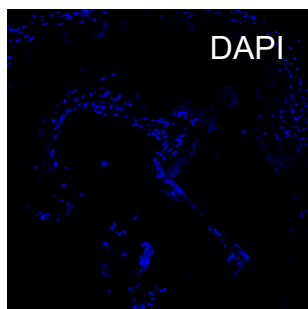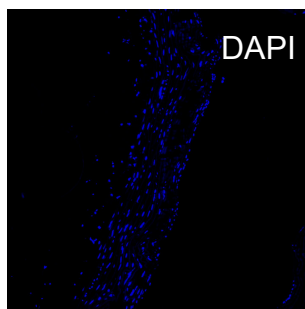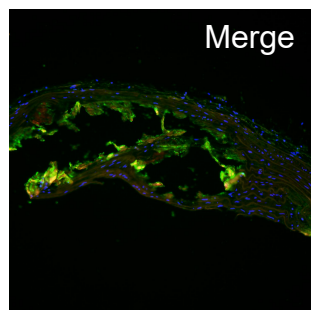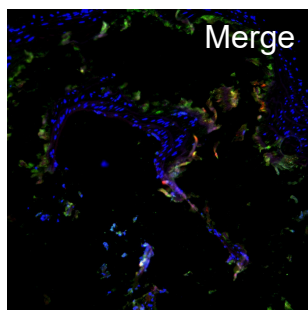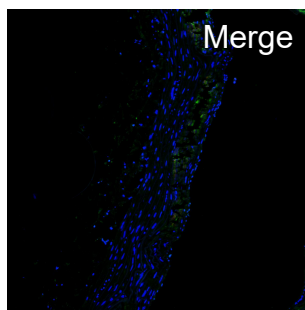

b

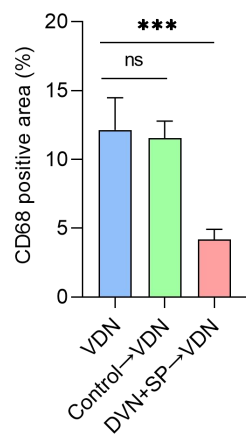

c

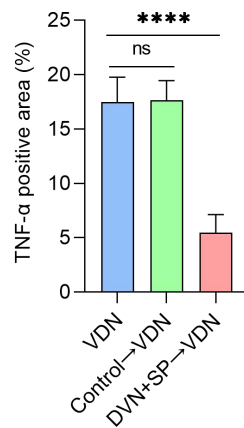

Supplement: Supplementary file 15 — Additional file 14: Supplementary Figure 8. Attenuation of macrophage infiltration and TNF-α expression in calcified vessel walls by the propionate-modulated intestinal microbiota. (a) Immunofluorescence staining for macrophages and TNF-α in calcified vessel walls (original magnification×100). (b) Quantitative analysis of the CD68-positive area. (c) Quantitative analysis of the TNF-α-positive area. Data are presented as the mean ± standard deviation (SD). Statistical significance was determined using one-way ANOVA (Tukey post hoc test). NS for P > 0.05, ***P< 0.001, ****P< 0.0001. [file 40168_2022_1390_MOESM14_ESM.pdf]

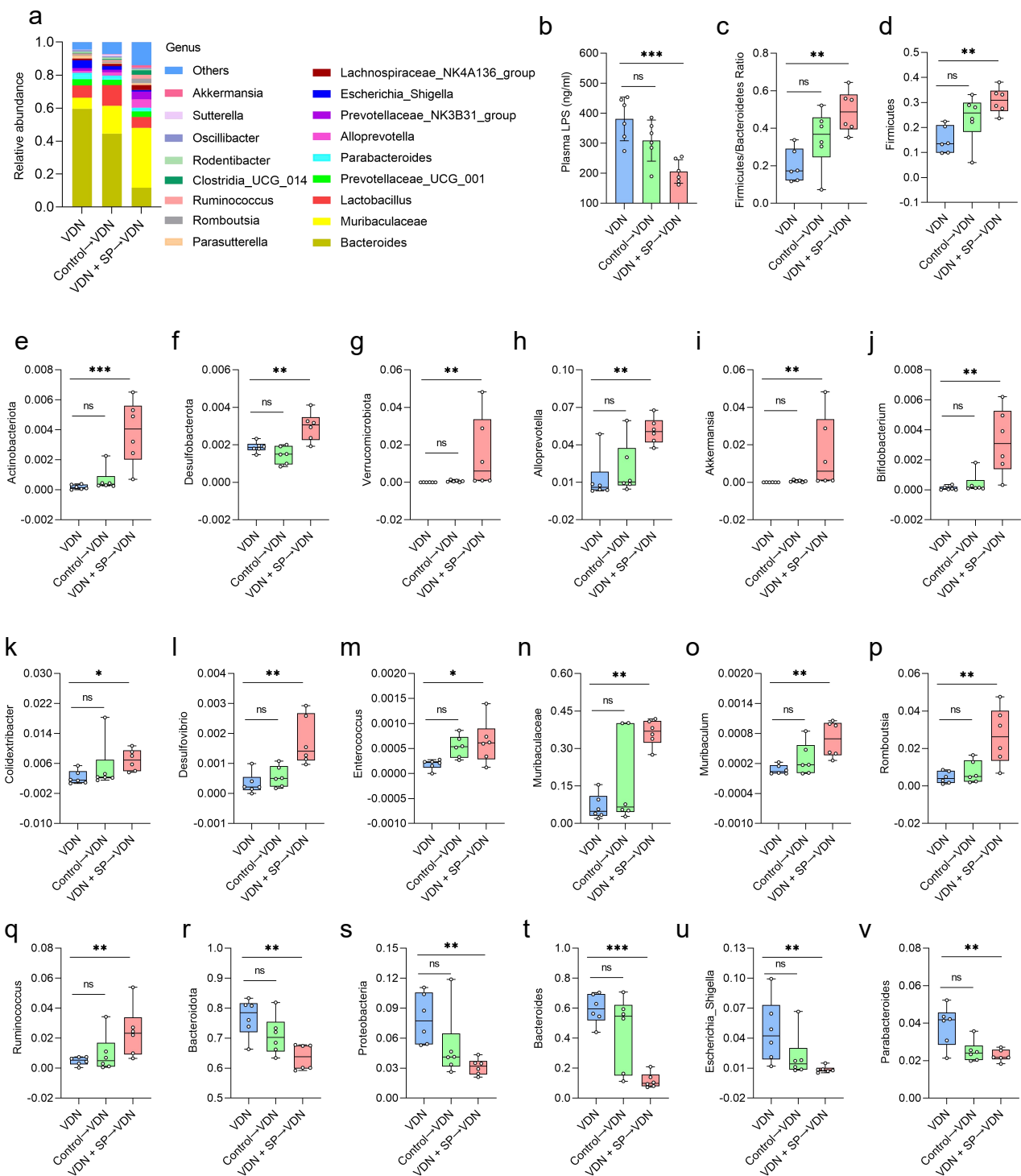

Supplement: Supplementary file 17 — Additional file 16: Supplementary Figure 9. The propionate-mediated intestinal microbiota attenuated VDN-induced microbial dysbiosis in rats. (a) Relative abundance of intestinal microbiota constituents at the genus level. (b) Plasma LPS levels. (c) Ratio between the relative abundance of Firmicutes and Bacteroidetes. (d-v) Relative abundance of identified differentially abundant bacterial groups at different taxonomic levels. Data are presented as the mean ± standard deviation (SD). Statistical significance was determined using one-way ANOVA or the Kruskal–Wallis test (Tukey or Dunnett post hoc test). NS for P > 0.05, *P<0.05, **P<0.01, ***P<0.001. [file 40168_2022_1390_MOESM16_ESM.pdf]

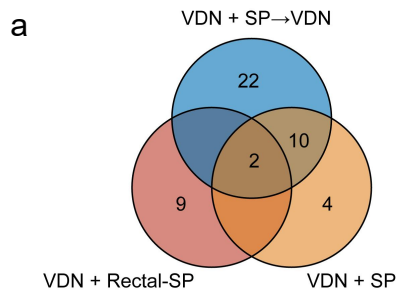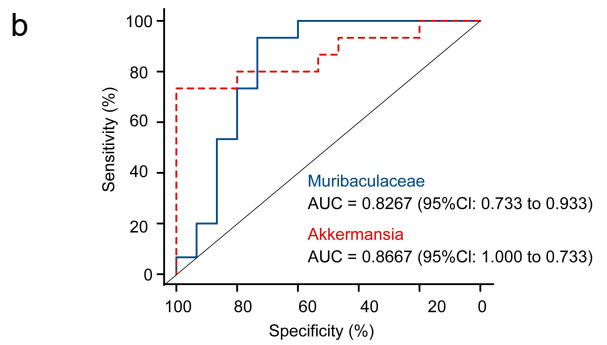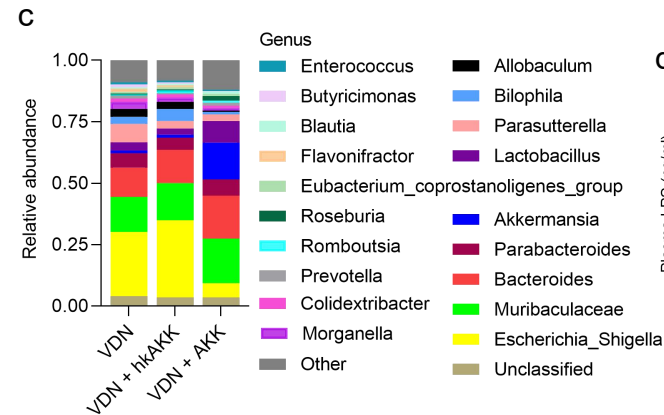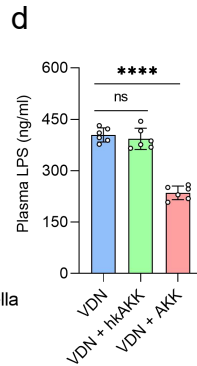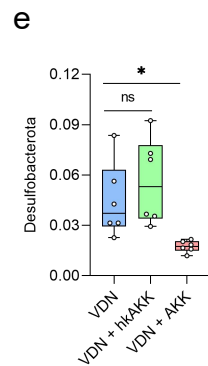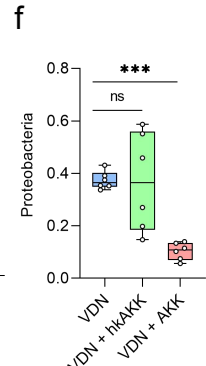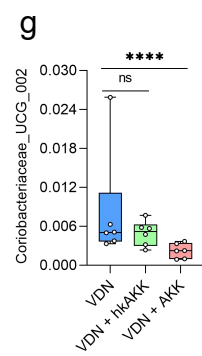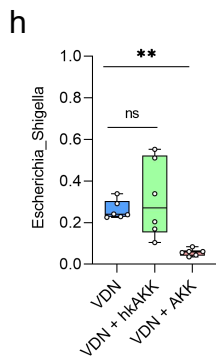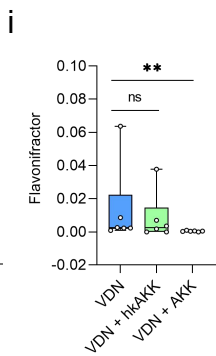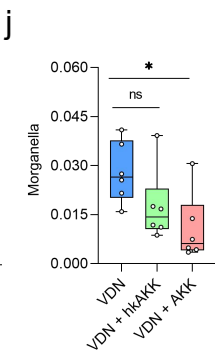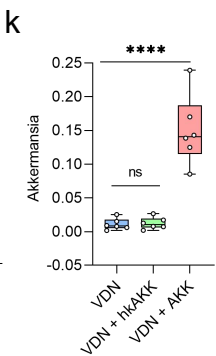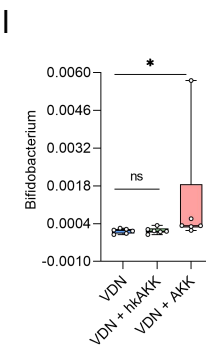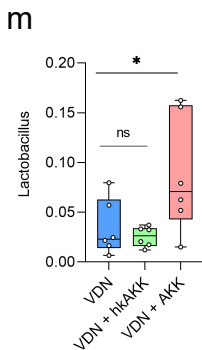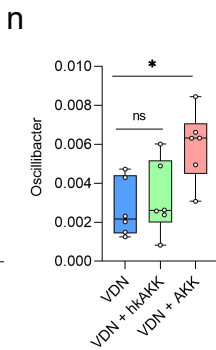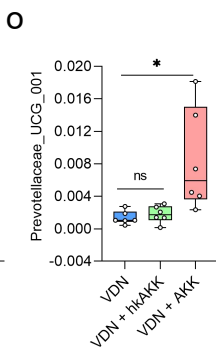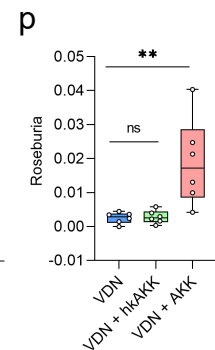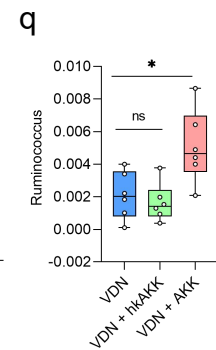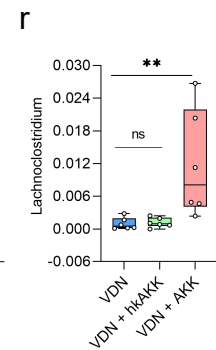

Supplement: Supplementary file 18 — Additional file 17: Supplementary Figure 10. Attenuation of VDN-induced microbial dysbiosis in rats by Akkermansia. (a) Venn diagram showing gut microbiota constituents coenriched by oral and rectal propionate administration and propionate-modulated intestinal microbiota transplantation. (b) Receiver operating characteristic (ROC) curves showing the ability of Muribaculaceae and Akkermansia abundance to predict vascular calcification. (c) Relative abundance of intestinal microbiota constituents at the genus level. (d) Plasma LPS levels. (e-r) Relative abundance of identified differentially abundant bacterial groups at different taxonomic levels. Data are presented as the mean ± standard deviation (SD). Statistical significance was determined using one-way ANOVA or the Kruskal–Wallis test (Tukey or Dunnett post hoc test). NS for P > 0.05, *P<0.05, **P<0.01, ***P<0.001, ****P<0.0001. [file 40168_2022_1390_MOESM17_ESM.pdf]

a

VDN

VDN + hkAKK

VDN + AKK

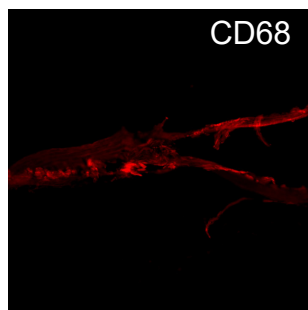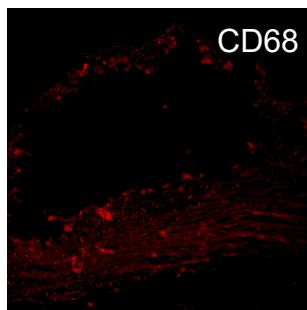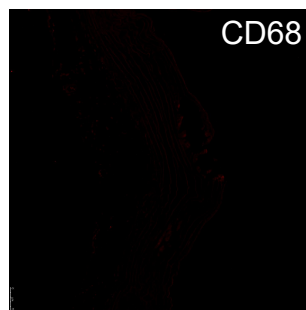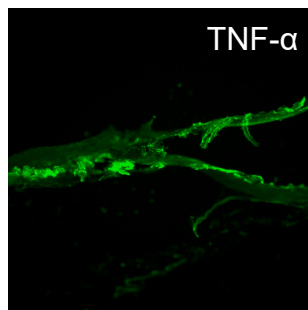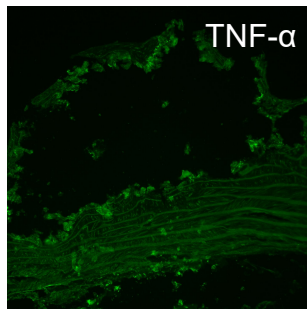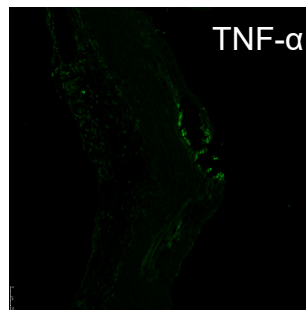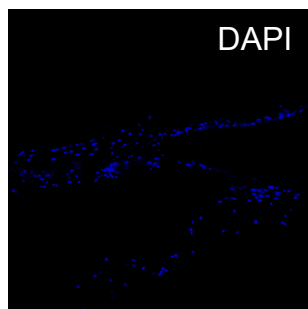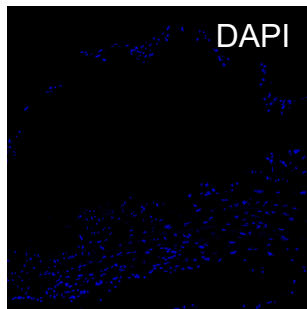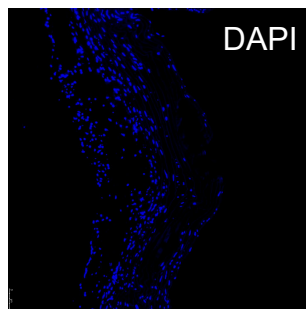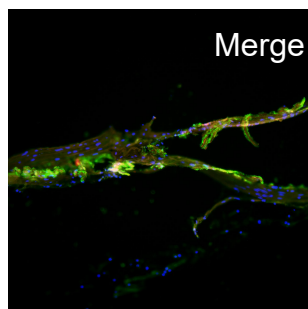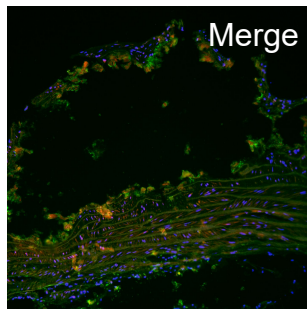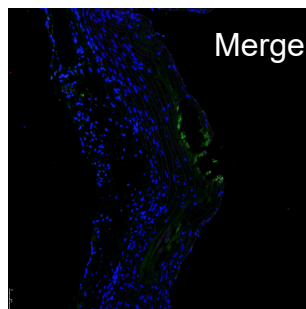

b

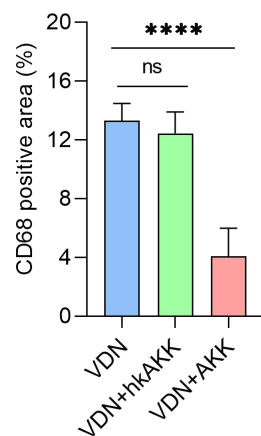

c

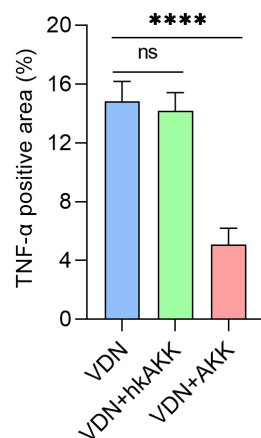

Supplement: Supplementary file 19 — Additional file 18: Supplementary Figure 11. Attenuation of macrophage infiltration and TNF-α expression in calcified vessel walls by Akkermansia. (a) Immunofluorescence staining for macrophages and TNF-α in calcified vessel walls (original magnification×100). (b) Quantitative analysis of the CD68-positive area. (c) Quantitative analysis of the TNF-α-positive area. Data are presented as the mean ± standard deviation (SD). Statistical significance was determined using one-way ANOVA (Tukey post hoc test). NS for P > 0.05, ***P< 0.001, ****P< 0.0001. [file 40168_2022_1390_MOESM18_ESM.pdf]

**a**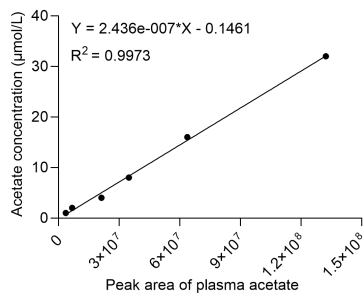**b**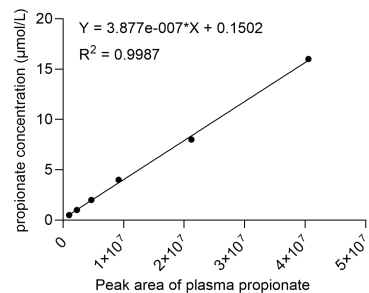**c**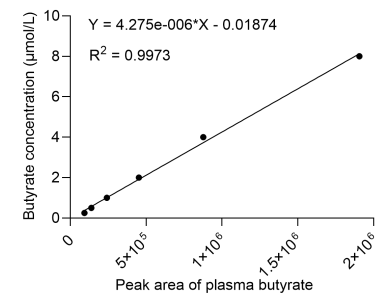**d**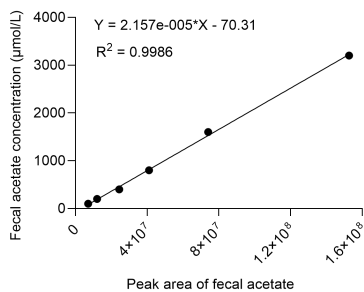**e**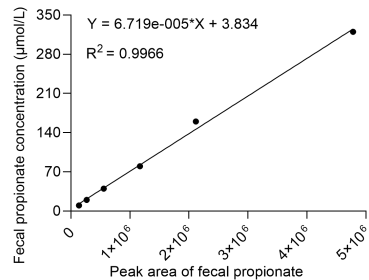**f**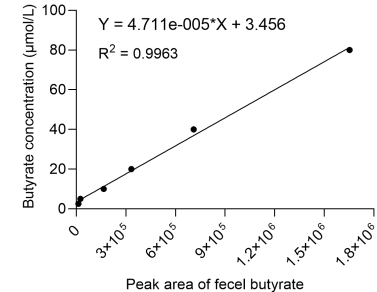**g**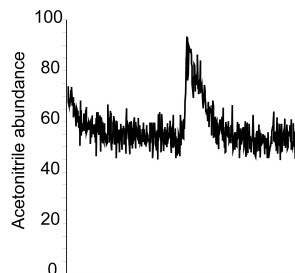**h**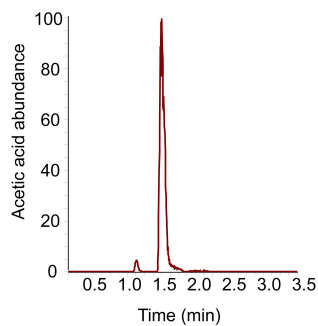

Supplement: Supplementary file 24 — Additional file 23: Supplementary Figure 12. SCFAs for external standard curves and control environmental acetate contamination. (a), (b) and (c) External standard curves of short-chain fatty acids (acetic acid, propionate, butyrate) standards in plasma samples. (d), (e) and (f) External standard curves of short-chain fatty acids (acetic acid, propionate, butyrate) standards in faecal samples. (g) Peak time of acetonitrile. (h) Peak time of acetic acid. [file 40168_2022_1390_MOESM23_ESM.pdf]
