# Supplementary material for: Beneficial effect of the short-chain fatty acid propionate on vascular calcification through intestinal microbiota remodelling
Source: Microbiome. 2022 Nov 16;10:195. doi: 10.1186/s40168-022-01390-0 (PMC9667615; doi:10.1186/s40168-022-01390-0)
Supplement: Supplementary file 11 — Additional file 10: Supplementary Table 5. Effect of oral propionate administration on the gut microbiota composition. [file 40168_2022_1390_MOESM10_ESM.docx]

Supplementary Table 5. Effect of oral propionate administration on the gut microbiota composition.

| Phylum | group | R^2^ | P value |
| --- | --- | --- | --- |
| Actinobacteriota | All | 0.7309 | 0.001 |
|  | Control vs. VDN + SP | 0.7658 | 0.004 |
|  | Control vs. VDN | 0.5506 | 0.003 |
|  | VDN + SP vs. VDN | 0.6184 | 0.001 |
| Bacteroidota | All | 0.3419 | 0.031 |
|  | Control vs. VDN + SP | 0.0026 | 0.883 |
|  | Control vs. VDN | 0.4176 | 0.017 |
|  | VDN + SP vs. VDN | 0.4312 | 0.013 |
| Desulfobacterota | All | 0.4264 | 0.006 |
|  | Control vs. VDN + SP | 0.4091 | 0.002 |
|  | Control vs. VDN | 0.5644 | 0.005 |
|  | VDN + SP vs. VDN | 0.0522 | 0.562 |
| Firmicutes | All | 0.5386 | 0.001 |
|  | Control vs. VDN + SP | 0.2936 | 0.069 |
|  | Control vs. VDN | 0.6419 | 0.003 |
|  | VDN + SP vs. VDN | 0.3247 | 0.057 |
| Proteobacteria | All | 0.7254 | 0.001 |
|  | Control vs. VDN + SP | 0.4136 | 0.026 |
|  | Control vs. VDN | 0.8581 | 0.003 |
|  | VDN + SP vs. VDN | 0.5330 | 0.011 |
| Verrucomicrobiota | All | 0.2721 | 0.092 |
|  | Control vs. VDN + SP | 0.1953 | 0.103 |
|  | Control vs. VDN | 0.1585 | 0.264 |
|  | VDN + SP vs. VDN | 0.2757 | 0.143 |

Statistical signifcance was determined using PERMANOVA test. P value < 0.05 was considered statistically significant. SP: sodium propionate; VDN: Vitamin D3 and nicotine.
