# Supplementary material for: Beneficial effect of the short-chain fatty acid propionate on vascular calcification through intestinal microbiota remodelling
Source: Microbiome. 2022 Nov 16;10:195. doi: 10.1186/s40168-022-01390-0 (PMC9667615; doi:10.1186/s40168-022-01390-0)
Supplement: Supplementary file 12 — Additional file 11: Supplementary Table 6. Effect of rectal propionate administration on the gut microbiota composition. [file 40168_2022_1390_MOESM11_ESM.docx]

Supplementary Table 6. Effect of rectal propionate administration on the gut microbiota composition.

| Phylum | Group | R^2^ | P value |
| --- | --- | --- | --- |
| Actinobacteriota | All | 0.2331 | 0.121 |
|  | VDN + Rectal-SP vs. VDN | 0.1303 | 0.219 |
|  | VDN + Rectal-SP vs. VDN + Rectal-SC | 0.2485 | 0.077 |
|  | VDN vs. VDN + Rectal-SC | 0.1406 | 0.21 |
| Bacteroidota | All | 0.6642 | 0.001 |
|  | VDN + Rectal-SP vs. VDN | 0.5256 | 0.01 |
|  | VDN + Rectal-SP vs. VDN + Rectal-SC | 0.7400 | 0.007 |
|  | VDN vs. VDN + Rectal-SC | 0.2796 | 0.066 |
| Desulfobacterota | All | 0.4245 | 0.008 |
|  | VDN + Rectal-SP vs. VDN | 0.2367 | 0.073 |
|  | VDN + Rectal-SP vs. VDN + Rectal-SC | 0.5032 | 0.014 |
|  | VDN vs. VDN + Rectal-SC | 0.2342 | 0.094 |
| Firmicutes | All | 0.0729 | 0.601 |
|  | VDN + Rectal-SP vs. VDN | 0.0852 | 0.346 |
|  | VDN + Rectal-SP vs. VDN + Rectal-SC | 0.0315 | 0.616 |
|  | VDN vs. VDN + Rectal-SC | 0.0439 | 0.513 |
| Proteobacteria | All | 0.5676 | 0.001 |
|  | VDN + Rectal-SP vs. VDN | 0.4897 | 0.004 |
|  | VDN + Rectal-SP vs. VDN + Rectal-SC | 0.5141 | 0.002 |
|  | VDN vs. VDN + Rectal-SC | 0.0648 | 0.454 |
| Verrucomicrobiota | All | 0.6712 | 0.001 |
|  | VDN + Rectal-SP vs. VDN | 0.6378 | 0.003 |
|  | VDN + Rectal-SP vs. VDN + Rectal-SC | 0.7839 | 0.004 |
|  | VDN vs. VDN + Rectal-SC | 0.0406 | 0.871 |

Statistical signifcance was determined using PERMANOVA test. P value < 0.05 was considered statistically significant. SP: sodium propionate; VDN: Vitamin D3 and nicotine.
