# Supplementary material for: Beneficial effect of the short-chain fatty acid propionate on vascular calcification through intestinal microbiota remodelling
Source: Microbiome. 2022 Nov 16;10:195. doi: 10.1186/s40168-022-01390-0 (PMC9667615; doi:10.1186/s40168-022-01390-0)
Supplement: Supplementary file 16 — Additional file 15: Supplementary Table 7. Effect of propionate-modulated intestinal microbiota on the gut microbiota composition. [file 40168_2022_1390_MOESM15_ESM.docx]

Supplementary Table 7. Effect of propionate-modulated intestinal microbiota on the gut microbiota composition.

| Phylum | Group | R^2^ | P value |
| --- | --- | --- | --- |
| Actinobacteriota | All | 0.4551 | 0.002 |
|  | VDN + SP→VDN vs. Control→VDN | 0.5079 | 0.003 |
|  | VDN + SP→VDN vs. VDN | 0.4537 | 0.003 |
|  | Control→VDN vs. VDN | 0.1480 | 0.124 |
| Bacteroidota | All | 0.5171 | 0.009 |
|  | VDN + SP→VDN vs. Control→VDN | 0.3452 | 0.042 |
|  | VDN + SP→VDN vs. VDN | 0.6442 | 0.011 |
|  | Control→VDN vs. VDN | 0.2092 | 0.127 |
| Desulfobacterota | All | 0.5701 | 0.001 |
|  | VDN + SP→VDN vs. Control→VDN | 0.5830 | 0.002 |
|  | VDN + SP→VDN vs. VDN | 0.5423 | 0.005 |
|  | Control→VDN vs. VDN | 0.2477 | 0.095 |
| Firmicutes | All | 0.4069 | 0.012 |
|  | VDN + SP→VDN vs. Control→VDN | 0.1415 | 0.124 |
|  | VDN + SP→VDN vs. VDN | 0.6700 | 0.004 |
|  | Control→VDN vs. VDN | 0.2022 | 0.131 |
| Proteobacteria | All | 0.5092 | 0.003 |
|  | VDN + SP→VDN vs. Control→VDN | 0.1817 | 0.114 |
|  | VDN + SP→VDN vs. VDN | 0.7030 | 0.002 |
|  | Control→VDN vs. VDN | 0.3098 | 0.052 |
| Verrucomicrobiota | All | 0.3823 | 0.007 |
|  | VDN + SP→VDN vs. Control→VDN | 0.1770 | 0.105 |
|  | VDN + SP→VDN vs. VDN | 0.4396 | 0.019 |
|  | Control→VDN vs. VDN | 0.3885 | 0.055 |
| at the phylum level of all microbiota | All | 0.5102 | 0.007 |
|  | VDN + SP→VDN vs. Control→VDN | 0.2698 | 0.044 |
|  | VDN + SP→VDN vs. VDN | 0.6965 | 0.004 |
|  | Control→VDN vs. VDN | 0.2285 | 0.118 |

Statistical signifcance was determined using PERMANOVA test. P value < 0.05 was considered statistically significant. SP: sodium propionate; VDN: Vitamin D3 and nicotine.
