# Supplementary material for: Beneficial effect of the short-chain fatty acid propionate on vascular calcification through intestinal microbiota remodelling
Source: Microbiome. 2022 Nov 16;10:195. doi: 10.1186/s40168-022-01390-0 (PMC9667615; doi:10.1186/s40168-022-01390-0)
Supplement: Supplementary file 20 — Additional file 19: Supplementary Table 8. Effect of Akkermansia on the gut microbiota composition. [file 40168_2022_1390_MOESM19_ESM.docx]

Supplementary Table 8. Effect of *Akkermansia* on the gut microbiota composition.

| Phylum | Group | R^2^ | P value |
| --- | --- | --- | --- |
| Actinobacteriota | All | 0.2878 | 0.029 |
|  | VDN + AKK vs. VDN | 0.2527 | 0.053 |
|  | VDN + AKK vs. VDN + hkAKK | 0.3323 | 0.054 |
|  | VDN vs. VDN + hkAKK | 0.0500 | 0.809 |
| Bacteroidota | All | 0.2098 | 0.151 |
|  | VDN + AKK vs. VDN | 0.4528 | 0.017 |
|  | VDN + AKK vs. VDN + hkAKK | 0.2050 | 0.155 |
|  | VDN vs. VDN + hkAKK | 0.0363 | 0.601 |
| Campilobacterota | All | 0.1932 | 0.12 |
|  | VDN + AKK vs. VDN | 0.1221 | 0.179 |
|  | VDN + AKK vs. VDN + hkAKK | 0.2595 | 0.099 |
|  | VDN vs. VDN + hkAKK | 0.1214 | 0.231 |
| Cyanobacteria | All | 0.1541 | 0.231 |
|  | VDN + AKK vs. VDN | 0.1379 | 0.142 |
|  | VDN + AKK vs. VDN + hkAKK | 0.1826 | 0.117 |
|  | VDN vs. VDN + hkAKK | 0.0347 | 0.981 |
| Desulfobacterota | All | 0.5684 | 0.003 |
|  | VDN + AKK vs. VDN | 0.5835 | 0.002 |
|  | VDN + AKK vs. VDN + hkAKK | 0.6751 | 0.006 |
|  | VDN vs. VDN + hkAKK | 0.0581 | 0.469 |
| Firmicutes | All | 0.1905 | 0.209 |
|  | VDN + AKK vs. VDN | 0.1856 | 0.145 |
|  | VDN + AKK vs. VDN + hkAKK | 0.1960 | 0.145 |
|  | VDN vs. VDN + hkAKK | 0.0095 | 0.84 |
| Proteobacteria | All | 0.6847 | 0.001 |
|  | VDN + AKK vs. VDN | 0.8585 | 0.006 |
|  | VDN + AKK vs. VDN + hkAKK | 0.5740 | 0.002 |
|  | VDN vs. VDN + hkAKK | 0.0770 | 0.395 |
| Verrucomicrobiota | All | 0.6276 | 0.001 |
|  | VDN + AKK vs. VDN | 0.7051 | 0.005 |
|  | VDN + AKK vs. VDN + hkAKK | 0.6901 | 0.005 |
|  | VDN vs. VDN + hkAKK | 0.0058 | 0.873 |

Statistical signifcance was determined using PERMANOVA test. P value < 0.05 was considered statistically significant. AKK: Akkermansia; hkAKK: Heat-killed Akkermansia; VDN: Vitamin D3 and nicotine.
