# Supplementary material for: Beneficial effect of the short-chain fatty acid propionate on vascular calcification through intestinal microbiota remodelling
Source: Microbiome. 2022 Nov 16;10:195. doi: 10.1186/s40168-022-01390-0 (PMC9667615; doi:10.1186/s40168-022-01390-0)
Supplement: Supplementary file 21 — Additional file 20: Supplementary Table 9. Questionnaire for China Prime Diet Quality Score. [file 40168_2022_1390_MOESM20_ESM.docx]

Supplementary Table 9. Questionnaire for China Prime Diet Quality Score.

| Healthy food | Average daily intake (g/d) | | | | | |
| --- | --- | --- | --- | --- | --- | --- |
|  | 0 | 1 | 2 | 3 | 4 | Score |
| Dark red /orange vegetables | 0 | 1-19 | 20-39 | 40-59 | ≥60 |  |
| Other vegetables | 0 | 1-39 | 40-79 | 80-119 | ≥120 |  |
| Dark yellow fruits | 0 | 1-19 | 20-39 | 40-59 | ≥60 |  |
| Citrus fruits | 0 | 1-19 | 20-39 | 40-59 | ≥60 |  |
| Other fruits | 0 | 1-19 | 20-39 | 40-59 | ≥60 |  |
| Nuts | 0 | 1-3 | 4-7 | 8-11 | ≥12 |  |
| Poultry | 0 | 1-9 | 10-19 | 20-29 | ≥30 |  |
| Milk | 0 | 1-59 | 60-119 | 120-179 | ≥180 |  |
| Eggs | 0 | 1-9 | 10-19 | 20-29 | ≥30 |  |
| Healthy food | Average daily intake (g/d) | | | | | |
|  | 0 | 2 | 4 | 6 | 8 | Score |
| Dark green vegetables | 0 | 1-19 | 20-39 | 40-59 | ≥60 |  |
| Soybean | 0 | 1-3 | 4-7 | 8-11 | ≥12 |  |
| Fish and shrimp | 0 | 1-9 | 10-19 | 20-29 | ≥30 |  |
| Whole grains/legumes | 0 | 1-9 | 10-19 | 20-29 | ≥30 |  |
| Healthy food | Average daily intake (g/d) | | | | | |
|  | 0 | 0.5 | 1 | 1.5 | 2 | Score |
| Sweet potatoes | 0 | 1-19 | 20-39 | 40-59 | ≥60 |  |
| Other potatoes | 0 | 1-19 | 20-39 | 40-59 | ≥60 |  |
| Limited food | Average daily intake (g/d) | | | | | |
|  | 4 | 3 | 2 | 1 | 0 | Score |
| Red meat | ≤50 | 51-100 | 101-150 | 151-200 | ＞200 |  |
| Fried food | ≤50 | 51-100 | 101-150 | 151-200 | ＞200 |  |
| Refined grains | 1-150 | 151-300 | 301-450 | 451-600 | ＞600 |  |
| Sugar sweetered Beverages | ≤100 | 101-200 | 201-300 | 301-400 | ＞400 |  |
| Cooking oil | ≤25 | 26-50 | 51-75 | 76-100 | ＞100 |  |
| Salt | ≤5 | 6-10 | 11-15 | 16-20 | ＞20 |  |
| Alcohol | ≤15 | 16-30 | 31-45 | 46-60 | ＞60 |  |
| Total |  | | | | |  |

Note: (1) Soybeans and their products are measured by the weight of dry beans; (2) Drinks containing alcohol are converted to the net amount of alcohol; (3) The intake of various foods is calculated according to the raw weight of the edible portion (Unit: g/d).
