# Supplementary material for: Beneficial effect of the short-chain fatty acid propionate on vascular calcification through intestinal microbiota remodelling
Source: Microbiome. 2022 Nov 16;10:195. doi: 10.1186/s40168-022-01390-0 (PMC9667615; doi:10.1186/s40168-022-01390-0)
Supplement: Supplementary file 22 — Additional file 21: Supplementary Table 10. The average sequencing depth and standard deviation for each dataset in this study. [file 40168_2022_1390_MOESM21_ESM.docx]

Supplementary Table 10. The average sequencing depth and standard deviation for each dataset in this study.

| Group | Control | VDN | VDN + SP |
| --- | --- | --- | --- |
| Mean | 84632 | 26116 | 31385 |
| Standard deviation | 3520 | 3175 | 4011 |
|  |  |  |  |
| Group | VDN | Control→VDN | VDN+SP→VDN |
| Mean | 80813 | 81907 | 86549 |
| Standard deviation | 4842 | 4686 | 5024 |
|  |  |  |  |
| Group | VDN | VDN + hkAKK | VDN + AKK |
| Mean | 68660 | 67670 | 69076 |
| Standard deviation | 3204 | 2241 | 3059 |
|  |  |  |  |
| Group | VDN | VDN + Rectal-SC | VDN + Rectal-SP |
| Mean | 68050 | 70317 | 71899 |
| Standard deviation | 4376 | 4206 | 4119 |
