# Supplementary material for: Beneficial effect of the short-chain fatty acid propionate on vascular calcification through intestinal microbiota remodelling
Source: Microbiome. 2022 Nov 16;10:195. doi: 10.1186/s40168-022-01390-0 (PMC9667615; doi:10.1186/s40168-022-01390-0)
Supplement: Supplementary file 23 — Additional file 22: Supplementary Table 11. Spiked recoveries and relative standard deviations of short chain fatty acids in plasma and feces. [file 40168_2022_1390_MOESM22_ESM.docx]

Supplementary Table 11. Spiked recoveries and relative standard deviations of short chain fatty acids in plasma and feces.

|  | Faecal acetate | | | Faecal propionate | | | Faecal butyrate | | |
| --- | --- | --- | --- | --- | --- | --- | --- | --- | --- |
| Concentration gradient (mM) | 100 | 30 | 10 | 50 | 15 | 5 | 5 | 1.5 | 0.5 |
| Spiked recoveries (%) | 105.07 | 90.59 | 95.32 | 96.71 | 91.98 | 93.46 | 96.40 | 99.20 | 93.19 |
| Relative standard deviations (%) | 5.76 | 7.95 | 8.60 | 6.88 | 6.83 | 5.33 | 7.76 | 8.93 | 6.06 |
|  | Plasma acetate | | | Plasma propionate | | | Plasma butyrate | | |
| Concentration gradient (μM) | 100 | 30 | 10 | 50 | 15 | 5 | 5 | 1.5 | 0.5 |
| Spiked recoveries (%) | 93.39 | 95.19 | 98.71 | 90.77 | 95.81 | 99.24 | 103.29 | 98.68 | 94.92 |
| Relative standard deviations (%) | 4.55 | 8.49 | 6.57 | 6.49 | 8.13 | 7.83 | 9.65 | 8.38 | 9.13 |
