# Supplementary material for: Beneficial effect of the short-chain fatty acid propionate on vascular calcification through intestinal microbiota remodelling
Source: Microbiome. 2022 Nov 16;10:195. doi: 10.1186/s40168-022-01390-0 (PMC9667615; doi:10.1186/s40168-022-01390-0)
Supplement: Supplementary file 25 — Additional file 24: Supplementary Table 12. Univariate and multivariate regression analysis of risk factors for vascular calcification in the participants with plasma samples. [file 40168_2022_1390_MOESM24_ESM.docx]

Supplementary Table 12. Univariate and multivariate regression analysis of risk factors for vascular calcification in the participants with plasma samples.

| Exposure | Univariate regression analysis | | Multivariate regression analysis | |
| --- | --- | --- | --- | --- |
|  | OR (95% CI) | P value | OR (95% CI) | P value |
| Age | 1.090 (0.999, 1.190) | 0.05308 | 1.178 (1.010, 1.373) | 0.0364 |
| Male | 4.812 (0.565, 40.972) | 0.15045 | 7.900 (0.401, 155.802) | 0.17428 |
| DM | 2.110 (0.688, 6.473) | 0.19177 | 1.289 (0.206, 8.082) | 0.78636 |
| Smoking | 1.503 (0.613, 3.683) | 0.37303 | 2.069 (0.550, 7.784) | 0.28228 |
| Drinking | 3.077 (0.914, 10.357) | 0.06952 | 4.522 (0.065, 316.758) | 0.48641 |
| TC | 1.318 (1.025, 1.695) | 0.03126 | 2.898 (0.813, 10.331) | 0.10087 |
| LDL-C | 1.089 (0.814, 1.458) | 0.56524 | 1.137 (0.525, 2.463) | 0.74388 |
| FBG | 1.146 (0.966, 1.360) | 0.11753 | 1.677 (0.598, 4.703) | 0.32544 |
| Uric acid | 1.002 (0.998, 1.006) | 0.34299 | 1.001 (0.991, 1.011) | 0.86159 |
| eGFR | 0.994 (0.966, 1.022) | 0.65194 | 1.128 (0.920, 1.383) | 0.24792 |
| BUN | 1.224 (0.912, 1.644) | 0.17859 | 5.317 (0.584, 48.440) | 0.13824 |
| BMI | 1.082 (0.972, 1.204) | 0.15072 | 1.201 (1.015, 1.422) | 0.0332 |
| CPDQS | 0.981 (0.927, 1.038) | 0.50252 | 0.846 (0.740, 0.968) | 0.01485 |
| log2 (acetate) | 0.651 (0.299, 1.417) | 0.27936 | 0.818 (0.259, 2.586) | 0.7326 |
| log2 (propionate) | 0.344 (0.219, 0.540) | <0.00001 | 0.158 (0.047, 0.533) | 0.00296 |
| log2 (butyrate) | 0.575 (0.440, 0.752) | 0.00005 | 0.389 (0.221, 0.685) | 0.00106 |

P value < 0.05 was considered statistically significant. BMI: Body Mass Index; BUN: Blood urea nitrogen; CPDQS: China Prime Diet Quality Score; MD: Diabetes mellitus; EGFR: Estimated glomerular filtration rate; FBG: Fasting blood glucose; LDL-C: low-density lipoprotein cholesterol; TC: Total cholesterol.
