# Supplementary material for: Beneficial effect of the short-chain fatty acid propionate on vascular calcification through intestinal microbiota remodelling
Source: Microbiome. 2022 Nov 16;10:195. doi: 10.1186/s40168-022-01390-0 (PMC9667615; doi:10.1186/s40168-022-01390-0)
Supplement: Supplementary file 26 — Additional file 25: Supplementary Table 13. Univariate and multivariate regression analysis of risk factors for vascular calcification in the participants with faecal samples. [file 40168_2022_1390_MOESM25_ESM.docx]

Supplementary Table 13. Univariate and multivariate regression analysis of risk factors for vascular calcification in the participants with faecal samples.

| Exposure | Univariate regression analysis | | Multivariate regression analysis | |
| --- | --- | --- | --- | --- |
|  | OR (95% CI) | P value | OR (95% CI) | P value |
| Age | 1.086 (0.987, 1.195) | 0.08965 | 2.015 (1.162, 3.494) | 0.01263 |
| Male | 1.667 (0.299, 9.301) | 0.56033 | 9.443 (0.001, 89091.348) | 0.63063 |
| DM | 3.408 (0.886, 13.110) | 0.07448 | 5.083 (0.282, 91.687) | 0.27054 |
| Smoking | 1.209 (0.435, 3.363) | 0.71627 | 1.368 (0.280, 6.686) | 0.69848 |
| Drinking | 2.160 (0.585, 7.979) | 0.24802 | 3.690 (0.625, 21.798) | 0.14972 |
| TC | 1.415 (1.076, 1.862) | 0.01305 | 1.362 (0.693, 2.678) | 0.37028 |
| LDL-C | 1.984 (1.272, 3.097) | 0.00254 | 2.142 (1.064, 4.313) | 0.03282 |
| FBG | 1.508 (1.125, 2.020) | 0.00592 | 1.384 (0.714, 2.682) | 0.33648 |
| Uric acid | 1.001 (0.996, 1.006) | 0.67396 | 1.011 (0.995, 1.026) | 0.17892 |
| eGFR | 0.995 (0.964, 1.028) | 0.77358 | 0.965 (0.852, 1.094) | 0.58047 |
| BUN | 1.165 (0.829, 1.637) | 0.37856 | 1.178 (0.348, 3.981) | 0.79219 |
| BMI | 1.759 (1.318, 2.348) | 0.00013 | 9.184 (0.811, 103.958) | 0.07329 |
| CPDQS | 0.948 (0.886, 1.015) | 0.1245 | 0.994 (0.878, 1.124) | 0.91823 |
| log2 (acetate) | 0.316 (0.098, 1.022) | 0.05434 | 0.696 (0.047, 10.363) | 0.7924 |
| log2 (propionate) | 0.400 (0.260, 0.616) | 0.00003 | 0.168 (0.038, 0.744) | 0.0188 |
| log2 (butyrate) | 0.593 (0.404, 0.871) | 0.00777 | 0.482 (0.243, 0.959) | 0.03748 |

P value < 0.05 was considered statistically significant. BMI: Body Mass Index; BUN: Blood urea nitrogen; CPDQS: China Prime Diet Quality Score; MD: Diabetes mellitus; EGFR: Estimated glomerular filtration rate; FBG: Fasting blood glucose; LDL-C: low-density lipoprotein cholesterol; TC: Total cholesterol.
